# Steric hindrance-engineered porous fluorescent films for ultrafast and ultrasensitive detection of nerve agent simulants

**DOI:** 10.1039/d5sc05184c

**Published:** 2025-08-18

**Authors:** Yuxuan Liu, Min Qiao, Jiali Liu, Gege Wang, Siyue Wang, Ruijuan Wen, Yaxin Zhai, Liping Ding, Xiaolin Zhu, Yu Fang

**Affiliations:** a Shaanxi Key Laboratory of New Concept Sensors and Molecular Materials, Key Laboratory of Applied Surface and Colloid Chemistry (Ministry of Education), School of Chemistry and Chemical Engineering, Shaanxi Normal University Xi'an 710119 P.R. China xiaolinchem@snnu.edu.cn dinglp33@snnu.edu.cn; b Key Laboratory of Low-Dimensional Quantum Structures and Quantum Control of Ministry of Education, Department of Physics, Hunan Normal University Changsha 410081 P.R. China yzhai@hunnu.edu.cn

## Abstract

Addressing the critical challenge of simultaneous high solid-state photoluminescence and efficient analyte mass transport for vapor sensors, a steric hindrance-engineered boron difluoride complex film, BODIQU-*t*BuCZ, is reported for ultrasensitive detection of nerve agent simulant DCP. Strategic incorporation of bulky *tert*-butyl groups suppresses detrimental π–π stacking and creates a porous 3D network (38.76% free volume) for rapid mass transport. Comprehensive structural and spectroscopic analyses validate this design principle and its impact on photophysics and sensing kinetics. The resulting BODIQU-*t*BuCZ sensor achieves exceptional performance: an ultra-low LOD of 0.001 ppt, ultra-fast ∼3-second response, and remarkable stability over >50 cycles. Quantitative comparison confirms its superiority over state-of-the-art sensors. This work presents a generalizable steric engineering strategy for high-performance fluorescent films, offering a promising platform for real-time neurotoxic threat monitoring.

## Abstract 2

A steric-hindrance strategy overcomes the core challenge of solid-state fluorescence quenching and slow analyte diffusion, enabling a boron complex film to detect DCP at 0.001 ppt in ∼3 seconds.

## Graphical abstract

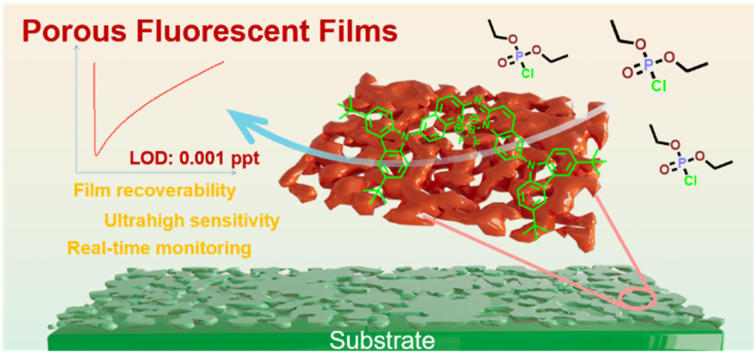

## Introduction

Neurotoxic agents pose severe and ongoing threats to human health and environmental safety. Despite long-standing international bans on chemical weapons, the continuous emergence of novel neurotoxins and the potential for their misuse mean that these chemicals remain a significant risk to public safety. Their capability to cause catastrophic harm and disrupt ecological balance globally highlights the critical importance of rapid and sensitive detection for effective early warning and response.^1,2^ As such, the detection and neutralization of these agents have become critical topics in national defence and security research.

Sarin, a prototypical organophosphorus nerve agent, exhibits extreme toxicity due to its irreversible inhibition of acetylcholinesterase, an essential enzyme responsible for terminating neural signals *via* acetylcholine hydrolysis.^3–5^ This inhibition triggers a cascade of physiological dysfunctions, including paralysis, respiratory failure, and ultimately death.^6,7^ Rapid and reliable detection of nerve agents at trace levels in real-time is therefore vital for timely medical intervention and environmental decontamination.^8^ Therefore, diethyl chlorophosphate (DCP), a structural analogue with reduced toxicity, is commonly used as a simulant for sarin and related agents in laboratory studies.^9,10^

A variety of techniques have been explored for nerve agent detection, including colorimetric assays,^11^ surface acoustic wave (SAW) sensors,^12,13^ photoacoustic spectroscopy,^14,15^ enzyme-based assays,^16,17^ electrochemical sensors,^18^ and gas chromatography-mass spectrometry (GC-MS).^19,20^ While offering valuable analytical capabilities, these methods often present significant limitations for practical field deployment, such as insufficient sensitivity, slow response times, low selectivity, and complex instrumentation, that hinder their practical deployment in the field.^21^ Consequently, there is an urgent need for alternative approaches that can overcome these challenges. Fluorescence-based sensors have emerged as a promising alternative, offering advantages such as ultrahigh sensitivity, fast response kinetics, low detection limits, and compatibility with portable and miniaturized devices.^22–24^

Fluorescence sensing strategies for nerve agents generally follow two mechanistic approaches: (i) direct phosphorylation of the probe by the agent or simulant^25^ and (ii) protonation of functional groups (*e.g.*, hydroxyl, amino, or pyridine) by hydrolysis products.^26–28^ Despite their inherent advantages, the practical utility of many fluorescent sensors is still restricted by several key challenges: (1) inadequate sensitivity for real-time detection at ultra-trace levels,^29^ (2) vulnerability to environmental interferents such as acidic vapors, structural analogues, and background fluorescence,^30^ and (3) intrinsic limitations of current recognition mechanisms.^31^ Therefore, the development of advanced fluorescent sensing materials with enhanced sensitivity, environmental tolerance, and rapid response is paramount for effective early warning systems against neurotoxic threats.

Boron-dipyrromethene (BODIPY) fluorophores have recently attracted considerable attention in the scientific community owing to their outstanding photophysical characteristics. As a representative class of organoboron complexes, these compounds demonstrate remarkable molar absorptivity, high fluorescence quantum yields, and exceptional chemical stability.^32–34^ These properties make them highly promising scaffolds for fluorescence sensing. Among various BODIPY derivatives, aza-BODIQU fluorophores are particularly attractive for nerve agent detection due to their superior photophysical performance.^35–37^ and, of special significance, aza-BODIQU derivatives exhibit selective reactivity with nerve agents *via* Lewis base-mediated protonation mechanisms.^38,39^ The structural flexibility of these compounds, afforded by multiple modifiable sites, enables precise customization of their sensing properties.^40^ However, a major challenge hindering the practical application of conventional aza-BODIQU derivatives, and many other fluorescent dyes, in vapor-phase thin-film sensors, is the severe solid-state fluorescence quenching. Molecular packing plays a crucial role in the emission performance of materials, particularly in the aggregated state. The relatively planar molecular geometry of these dyes often leads to strong intermolecular π–π stacking interactions in the condensed phase, promoting aggregation-caused quenching (ACQ) and thus severely limiting their practical applications.^41,42^ Furthermore, achieving simultaneously high sensitivity (often requiring a thicker film for analyte absorption) and rapid response speed (favored by a thin film for rapid diffusion) in such thin-film sensors represents a long-standing trade-off.

Herein, we report a novel strategy based on steric hindrance engineering to overcome the aforementioned limitations and achieve high-performance vapor-phase fluorescence sensing. Specifically, we incorporated bulky substituents into the aza-BODIQU core. This molecular design achieves three critical functions simultaneously: (i) suppression of intermolecular π–π interactions *via* steric repulsion, thereby mitigating solid-state fluorescence quenching; (ii) formation of a porous nanostructure that facilitates analyte diffusion throughout the film; and (iii) retention of strong fluorescence emission in the solid, collectively leading to enhanced sensitivity and ultra-fast response speed, thereby effectively addressing the long-standing trade-off between sensitivity and response speed in fluorescent film sensors.

**Scheme 1:**
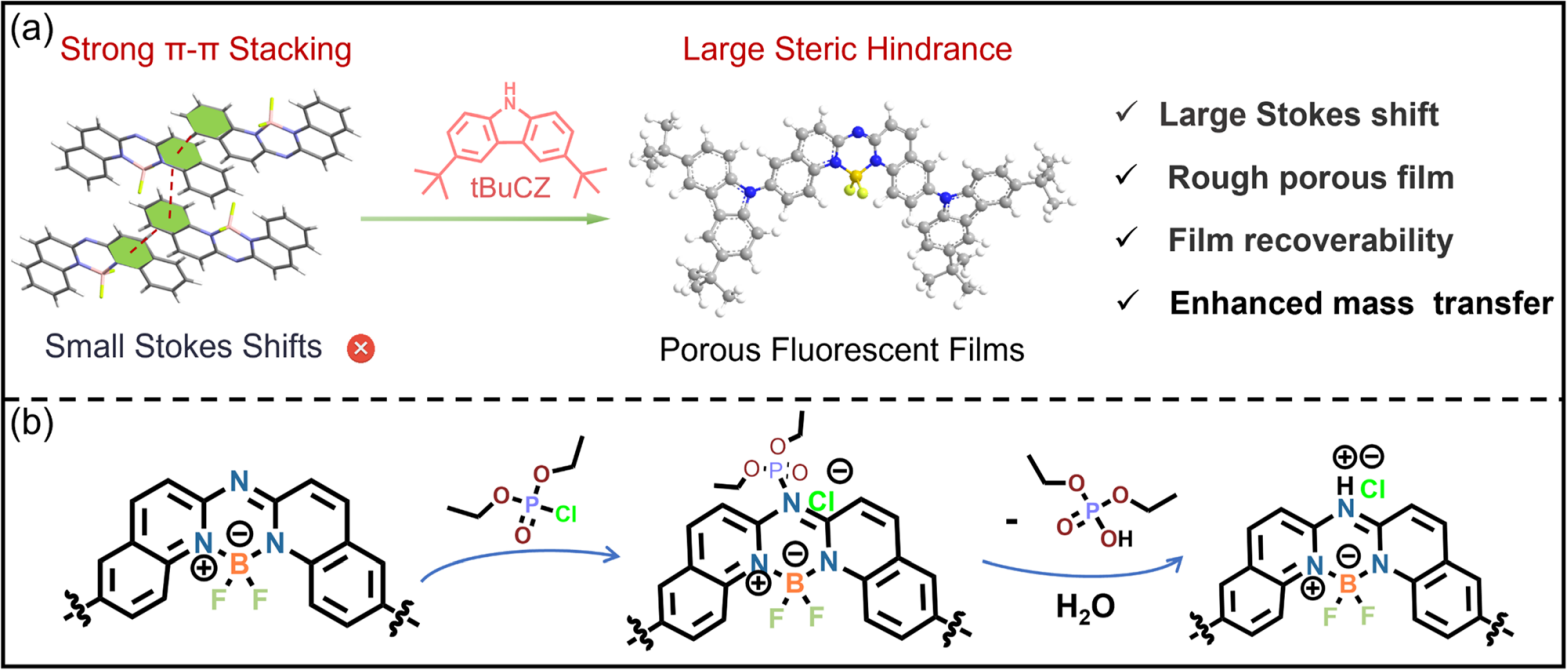
(a) Design strategy for achieving efficient analyte transport and high-sensitivity, real-time vapor sensing via steric hindrance-engineered fluorescent films, integrated into a homemade sensing platform. (b) Sensing mechanism of DCP by BIDQIQU core unit.

Building upon this strategy, in this study, we designed and synthesized a sterically engineered fluorescent material by coupling aza-BODIQU with 3,6-di-*tert*-butylcarbazole *via* Suzuki–Miyaura cross-coupling (Schemes 1 and S1). The incorporation of *tert*-butyl groups induces intramolecular twisting, disrupts dense molecular packing, and generates a porous film (BODIQU-*t*BuCZ) with a free volume of 38.76%. As hypothesized, this porous architecture significantly enhances vapor-phase analyte diffusion kinetics and simultaneously suppresses fluorescence quenching. Consequently, the resulting film sensor exhibits unprecedented performance for DCP vapor detection, demonstrating a detection limit of 0.001 ppt, an ultra-rapid 3-second response time, high selectivity against common interferents, and outstanding recyclability over 50 cycles. These results represent a significant advancement in the field of vapor-phase fluorescence sensing, offering a solution to the long-standing challenges of sensitivity and speed trade-off and solid-state quenching. Control experiments with unmodified carbazole and benzimidazole analogues confirmed the critical role of the steric bulk in achieving superior sensing properties. Our findings highlight a generalizable strategy for constructing high-performance porous fluorescent films *via* steric hindrance engineering, offering new opportunities for real-time monitoring of hazardous neurotoxic threats.

## Results and discussion

### Crystal structure and porosity of BODIQU-*t*BuCZ

**Fig. 1:**
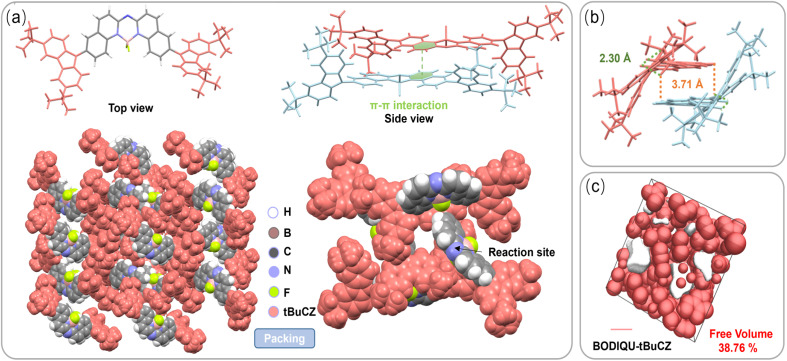
(a) Crystal structure of BODIQU-*t*BuCZ from the front and side views and spatial molecular arrangement and the packing mode of compound BODIQU-*t*BuCZ in the crystal. (b) Molecular stacking in the crystal for BODIQU-*t*BuCZ: green dotted lines: intramolecular C–H⋯F interaction (2.30–2.54 Å); orange dotted lines: intermolecular C–H⋯F interaction (3.71–3.73 Å). (c) Calculated free volume of BODIQU-*t*BuCZ in the crystalline state. The pink and white parts represent pores and compound molecules in the crystal cell.

To elucidate the structure–property relationships underpinning the optical and sensing performance of BODIQU-*t*BuCZ, single-crystal X-ray diffraction (XRD) analysis was performed (Fig. S1 and Table S1). As shown in Fig. 1, the crystal structure confirms that the bulky *tert*-butylcarbazole groups create a porous solid-state structure, which promotes high solid-state fluorescence. Recognizing that drop-cast thin films may not perfectly replicate the single-crystal arrangement, we first used Powder X-ray Diffraction (PXRD) to assess the powder structure. PXRD revealed higher crystallinity for BODIQU-*t*BuCZ powder compared to films of BODIQU-CZ and BODIQU-BZI (Fig. S2). This suggests that the bulky substituents hinder dense packing, consistent with the single-crystal structure. This looser packing and increased free volume in BODIQU-*t*BuCZ are crucial for efficient vapor transport and interaction with the sensing material in the resultant films, ultimately enhancing sensor performance. The observed molecular channels within the crystal lattice (Fig. 1c) suggest the potential for efficient mass transfer. To investigate the actual packing arrangement in the thin films, we employed Grazing Incidence Wide-Angle X-ray Scattering (GIWAXS) (Fig. S3). The GIWAXS patterns revealed distinct differences in molecular ordering and π–π stacking among the films. BODIQU-*t*BuCZ films exhibited well-defined Bragg diffraction peaks or arcs, indicative of a high degree of molecular order. Importantly, they lacked the characteristic diffraction peak associated with close π–π stacking. In contrast, BODIQU-BZI films displayed both Bragg diffraction features and a pronounced π–π stacking peak, while BODIQU-CZ films predominantly exhibited strong π–π stacking peaks, suggesting a higher degree of aggregation. These results confirm that the *tert*-butyl groups in BODIQU-*t*BuCZ effectively suppress π–π stacking during film formation, promoting an ordered yet non-aggregated molecular arrangement. This balance of order and reduced π–π interaction is crucial for achieving high free volume and enhanced guest molecule accessibility within the sensor film. This suppression of π–π stacking is evident in the single-crystal structure as well (Fig. 1a). The two *N-tert*-butylcarbazole rings are sterically forced to twist significantly out of the plane of the aza-BODIQU core, creating a non-coplanar structure that prevents tight π–π interactions in the solid state.

Fig. 1b shows the intramolecular C–H⋯F hydrogen bond distances ranging from 2.30 to 2.54 Å, while the weak intermolecular interactions between adjacent molecules fall within the range of 3.71 to 3.73 Å. These weak intermolecular interactions, combined with the twisted intramolecular geometry, result in the compound exhibiting strong fluorescence in the solid state by significantly reducing π–π stacking induced quenching. The formation of these hydrogen bonds may also contribute to stabilizing the twisted conformation and influencing the specific packing arrangement, potentially guiding the directional transport of the sensing molecules within the pores, thereby enhancing the mass transfer efficiency. Additionally, the dihedral angles between the *tert*-butyl carbazole groups and the core are 52.3° and 53.2°, respectively. These bulky groups profoundly disrupt the spatial arrangement of the BODIQU structure to some extent, making the overall structure more flexible. Importantly, the free volume within the material's lattice reaches a remarkable 38.76% (Fig. 1c), which strongly supports the beneficial effect of the *N-tert*-butylcarbazole group in enhancing the porous structure. The larger free volume provides more space for molecular movement and higher diffusivity, thereby accelerating the mass transfer process. Overall, the crystal structure compellingly demonstrates that the *tert*-butyl-induced twisted conformation is the key factor responsible for suppressing detrimental π–π stacking and generating a high free volume porous structure. This sophisticated structural design, robustly validated by the XRD, PXRD, and GIWAXS results, effectively mitigates unfavorable π–π stacking during thin film fabrication and promotes an ordered, yet non-aggregated, molecular arrangement. Such an architecture fundamentally establishes the foundation for achieving both high solid-state fluorescence quantum yield and rapid analyte diffusion, which are prerequisites for a high-performance vapor-phase fluorescent sensor.

### Mechanism of response for BODIQU-*t*BuCZ to DCP detection

To fully elucidate the molecular-level mechanism underlying the highly sensitive and rapid detection of nerve agent simulants using BODIQU-*t*BuCZ, we performed comprehensive spectroscopic and structural investigations comparing BODIQU-*t*BuCZ with its structural analogs, BODIQU-CZ and BODIQU-BZI. These studies aimed to track the interaction between BODIQU-*t*BuCZ and diethyl chlorophosphate (DCP) at the molecular level and understand how this interaction translates into a fluorescence response.

In solution, BODIQU-*t*BuCZ exhibited distinct photophysical behavior characterized by broad UV-vis absorption (438–505 nm, *λ*_max_ = 468 nm) and a pronounced redshift compared to BODIQU-BZI (391–443 nm, *λ*_max_ = 441 nm). This redshift is attributed to the strong electron-donating ability of the 3,6-di-*tert*-butylcarbazole moiety, which enhances intramolecular charge transfer (ICT) characteristics (Fig. S4). In addition, BODIQU-*t*BuCZ also demonstrated significantly larger Stokes shifts (82 nm) and green-region fluorescence emission (*λ*_em_ = 525 nm), in contrast to its blue-emitting counterpart BODIQU-BZI with a small Stokes shift (28 nm). The high molar extinction coefficient (44 000 L mol^−1^ cm^−1^) further indicated the enhanced light-harvesting capabilities enabled by molecular engineering (Fig. S5–S7). Solvent-dependent fluorescence lifetime studies (Fig. S8–S10 and Table S2) further support its ICT nature; the fluorescence lifetime of BODIQU-*t*BuCZ was markedly reduced in polar solvents, while it remained stable in nonpolar media such as toluene, suggesting that solvent polarity influences the charge separated state.

**Fig. 2:**
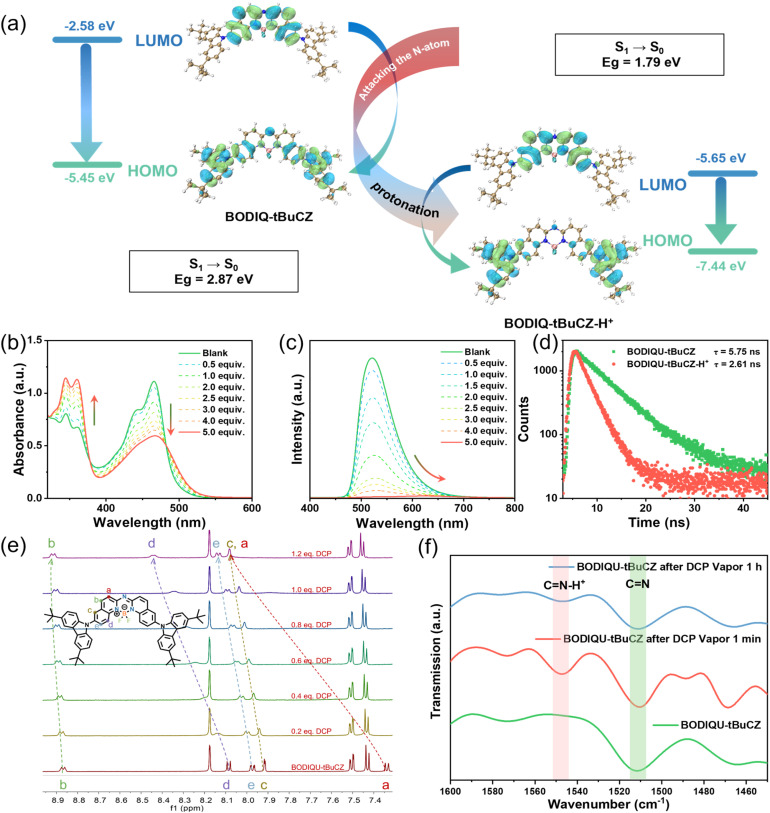
(a) Sensing mechanism of the BIDQIQU core unit and fluorescence response of BODIQU-tBuCZ solution to DCP, with the solution colour changing from green to orange. (b) UV-vis absorption spectra and (c) fluorescence emission spectra of BODIQU-*t*BuCZ titrated with DCP in DCM. (d) Lifetime spectra of BODIQU-*t*BuCZ before and after DCP treatment. (e) ^1^H NMR spectra of the compound BODIQU-*t*BuCZ with different equivalence ratios of DCP addition. (f) FT-IR spectra of BODIQU-*t*BuCZ before and after DCP treatment.

Given that the aza-BODIQU core in BODIQU-*t*BuCZ possesses inherent Lewis basicity, it is expected to be highly responsive to electrophilic or acidic species like DCP. Coupled with its high film-state fluorescence quantum yield (Table S3), BODIQU-*t*BuCZ is an ideal candidate for a turn-off type fluorescence sensor platform. To evaluate the protonation tendency and investigate the electronic structure changes of the BODIQU-*t*BuCZ molecule upon interaction with electrophilic species, density functional theory (DFT) calculations were performed at the B3LYP/6-311G* level (Fig. S11). This basis set incorporates polarization functions for heavy atoms, striking a good balance between computational accuracy and efficiency. Calculations were conducted for both the neutral BODIQU-*t*BuCZ and its protonated form, BODIQU-*t*BuCZ-H^+^. The optimized structures and frontier molecular orbital (FMO) distributions are presented in Fig. 2a. Analysis of the calculated electronic structure for the neutral BODIQU-*t*BuCZ revealed that the highest occupied molecular orbital (HOMO) electron density is primarily distributed on the electron-donating *t*-butyl carbazole moieties and the nitrogen atoms of the aza-BODIQU core. This spatial distribution of the HOMO indicates that these nitrogen sites are the most nucleophilic centers within the molecule and thus the most probable sites for electrophilic attack and protonation. The calculated HOMO energy for the neutral molecule was −5.45 eV, and the lowest unoccupied molecular orbital (LUMO) energy was −2.58 eV. Upon protonation at the aza-nitrogen atom of the BODIQU core, significant changes in the electronic structure were calculated. The calculated HOMO energy of the protonated species, BODIQU-*t*BuCZ-H^+^, decreased substantially to −7.44 eV, indicating a significant stabilization of the highest occupied levels. Concurrently, the LUMO energy shifted significantly to −5.65 eV.

This calculated pronounced reorganization of the frontier molecular orbital energies upon protonation provides strong theoretical support for the proposed sensing mechanism. The dramatic decrease in HOMO energy and shift in LUMO energy fundamentally alter the electronic structure and are consistent with the observed disruption of the intramolecular charge transfer (ICT) process that dominates the photophysics of neutral BODIQU-*t*BuCZ. Both BODIQU-*t*BuCZ and its protonated form (BODIQU-*t*BuCZ-H^+^) exhibit charge transfer (CT) character in their HOMO–LUMO transitions. However, protonation induces significant LUMO localization within the BODIQU core (Fig. 2a), where the electron density becomes concentrated near the protonation site. This localization, along with a sharp decrease in both HOMO and LUMO energies (narrowing the S_1_ → S_0_ gap from 2.87 eV to 1.79 eV), alters the excited-state relaxation pathways. The resulting enhanced non-radiative decay leads to the observed fluorescence quenching. These theoretical calculations confirm that protonation at the aza-nitrogen induces substantial electronic structural changes, directly impacting the photoluminescence properties and explaining the experimentally observed quenching.

Upon DCP exposure, a series of photophysical changes were observed (Fig. S12 and Table S4). In diluted dichloromethane, the UV-vis spectra showed the disappearance of the 465 nm absorption band, accompanied by increased intensity at 345 and 360 nm and the emergence of two well-defined isosbestic points (Fig. 2b), suggesting a well-defined molecular transformation rather than simple non-specific quenching.

Simultaneously, the fluorescence spectra revealed progressive quenching of the 525 nm emission peak with an increasing DCP concentration, accompanied by the appearance of a weak, red-shifted emission at 626 nm (Fig. 2c). These spectral changes closely resemble the response of BODIQU-*t*BuCZ upon protonation with *p*-toluenesulfonic acid (Fig. S13 and S14), strongly suggesting a protonation-induced sensing mechanism. Time-resolved photoluminescence (PL) measurements further confirmed this (Fig. S15). The excited-state lifetime of BODIQU-*t*BuCZ in solution significantly reduced from 5.75 ns to 2.61 ns upon DCP treatment (Fig. 2d), indicative of enhanced nonradiative decay pathways in the protonated state.

To provide direct molecular-level evidence for the proposed protonation, ^1^H NMR titration experiments were carried out by gradually adding DCP to BODIQU-*t*BuCZ in CDCl_3_. A continuous downfield shift of the aromatic proton signals (*e.g.*, from 8.05 ppm and 7.95 ppm) was observed upon DCP addition (Fig. 2e and S16). This spectral evolution is consistent with a stepwise electrophilic attack by DCP leading to the formation of a protonated species, BODIQU-*t*BuCZ-H^+^, where the positive charge is delocalized onto the aromatic system, causing the observed downfield shifts. This provides direct evidence of a strong electronic perturbation upon interaction with DCP.

Furthermore, Fourier-Transform Infrared (FT-IR) spectroscopy of BODIQU-*t*BuCZ thin films before and after exposure to DCP vapor revealed the emergence of a new band at 1550 cm^−1^ (Fig. 2f and S17). This band is characteristic of the C=N–H^+^ stretching vibration in protonated imine or aza systems.^43^ Its appearance upon DCP exposure firmly indicates that the nucleophilic nitrogen atom within the aza-BODIQU core serves as the primary site for electrophilic attack and subsequent protonation by DCP. This mechanism is further corroborated by XPS analysis, which revealed a characteristic binding energy shift of the 1s orbital of nitrogen (Fig. S18 and S19). Notably, when the BODIQU-*t*BuCZ film was left in air for one hour after DCP exposure, the intensity of the 1550 cm^−1^ peak significantly decreased, suggesting reversible protonation of the protonated species under ambient conditions. Further investigation revealed that this reversibility is not solely due to ambient exposure but is strongly influenced by environmental humidity and the presence of trace weakly basic components in the air that facilitate neutralization and removal of the protonated species. Regarding the role of counteranions (*e.g.*, Cl^−^ from DCP hydrolysis), their direct impact on the photophysical properties of the BODIQU core appears minimal. The observed fluorescence quenching is primarily attributed to the electronic structural changes arising from nitrogen protonation. However, counteranions may indirectly influence the sensing process. For instance, variations in anion size and hydrophilicity could affect their diffusion within the film—smaller, more hydrophilic anions like Cl^−^ are likely to diffuse more readily than bulkier, hydrophobic ones. Additionally, the charge density and geometry of the counteranion may influence its interaction with the protonated BODIQU-H^+^ core, potentially altering the local dielectric environment and subtly shifting the protonation/deprotonation equilibrium. These indirect effects could manifest as variations in fluorescence quenching efficiency or recovery kinetics. Although this study focuses on the response to HCl, a systematic investigation of counteranion-dependent effects on sensor performance warrants future exploration.

Collectively, these results firmly provide compelling evidence for a protonation-induced fluorescence quenching mechanism. Upon exposure to DCP, the lone pair on the central nitrogen atom of the BODIQU unit undergoes electrophilic attack, leading to the formation of a protonated species. This structural transformation fundamentally alters the electronic structure of the BODIQU core, effectively disrupting the original ICT process. The resulting protonated state enables efficient fluorescence quenching by enhancing non-radiative decay pathways. Crucially, the inherent porous solid-state structure of BODIQU-*t*BuCZ, as jointly supported and demonstrated by single-crystal XRD, PXRD and GIWAXS measurements (Fig. S2 and S3), facilitates rapid diffusion of volatile DCP molecules throughout the thin film, allowing for efficient interaction with the sensing material. Furthermore, the steric hindrance engineering suppresses detrimental intermolecular π–π stacking, ensuring high initial solid-state fluorescence quantum yield that is critical for sensitive turn-off detection. Therefore, the combination of the intrinsic protonation reactivity of the aza-BODIQU core and the uniquely engineered porous solid-state architecture is key to achieving the observed ultra-sensitive and rapid vapor-phase sensing performance. These results collectively demonstrate the successful tuning of the optoelectronic structure and solid-state packing of BODIQU-*t*BuCZ, establishing a strong basis for high-performance solid-state sensing applications.

### Solid-state sensing performance of BODIQU-*t*BuCZ films

Building upon the solution-phase titration and spectroscopic analyses, it is evident that BODIQU-*t*BuCZ undergoes a protonation reaction upon exposure to DCP, leading to the formation of the electron-deficient species BODIQU-*t*BuCZ-H^+^ and a corresponding color change from green to yellow (Fig. S20). This chemical transformation, accompanied by fluorescence quenching and absorption peak modulation, provides strong evidence for a specific chemical sensing mechanism rather than nonspecific physical adsorption. The distinct interaction pattern underscores the selectivity and reliability of the sensor platform. Furthermore, the observed rapid kinetics and high sensitivity of BODIQU-*t*BuCZ in the liquid-phase established a robust foundation for its application in gas-phase detection of nerve agent simulants.

Given the high volatility of nerve agents such as sarin, developing sensitive and durable thin-film-based gas sensors is of critical importance. Compared to solution-based systems, solid-state films offer improved analyte accessibility and are more readily integrated into device architectures. In this study, a custom-designed sensing platform was employed to assess the gas-phase detection performance of BODIQU-*t*BuCZ films. The sensor architecture adopts a laminated structure wherein the fluorescence emission at 490 nm, triggered by 365 nm LED excitation, is continuously monitored using a photodiode. DCP vapor was introduced into the sensing chamber *via* a micro-pump system from a pre-filled sampling bag, allowing precise control over exposure time and concentration (Fig. S21).

**Fig. 3:**
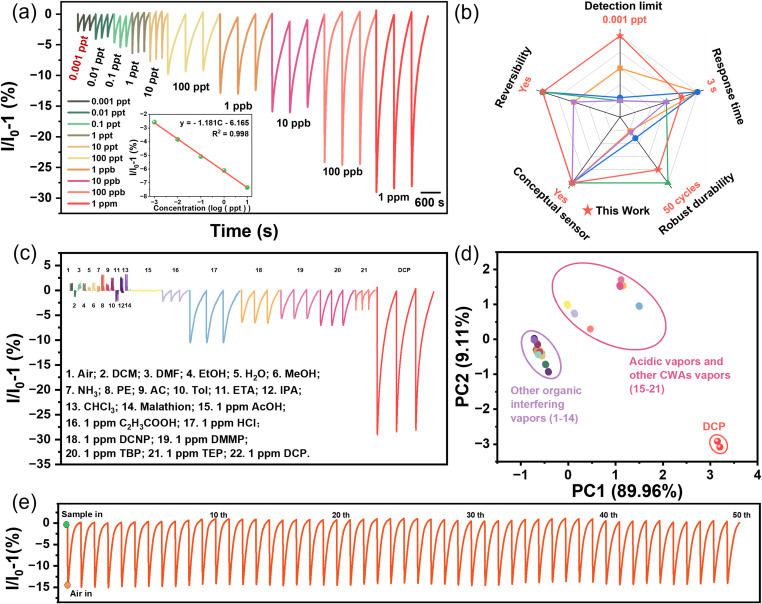
(a) Response intensity of the BODIQU-*t*BuCZ film to DCP vapor of different concentrations (0.001 ppt–1 ppm). (b) The comparison of DCP sensing performance of representative methods reported in the literature. (c) Response intensity of the sensor to the presence of DCP and other vapors. (d) Two-dimensional PCA score plot based on fluorescence response intensity, recovery time, and response time for discrimination of chemical analytes (*n* = 6 replicates per analyte). (e) Results from continuous recyclability tests using the film-based sensor in the presence of the vapors of 10 ppb DCP (50 cycles).

Sensing films were fabricated by drop-casting a diluted DCM solution of BODIQU-*t*BuCZ onto quartz glasses. The resulting films demonstrated excellent photostability, with negligible fluorescence degradation (<5%) observed after 12 hours of continuous UV irradiation. In stark contrast, control films made from BODIQU-CZ and BODIQU-BZI showed fluorescence quenching of approximately 20% and 40% (Fig. S22), respectively, under identical conditions, highlighting the superior intrinsic stability of the sterically hindered BODIQU-*t*BuCZ. Fluorescence intensity measurements were conducted over a wide range of DCP vapor concentrations (0.001 ppt to 1 ppm). As shown in Fig. 3a, the BODIQU-*t*BuCZ film exhibited a concentration-dependent quenching response, with a discernible signal reduction observed even at ultra-trace levels (0.001 ppt), corresponding to a quenching ratio of 2.6%. The response to DCP vapor was rapid, typically within a few seconds, and full recovery of fluorescence was observed upon removal of the DCP vapor followed by air purging, attesting to the reversible nature and excellent durability of the film during single exposure-recovery cycles. The experimentally determined detection limit (LOD) was found to be a remarkable 0.001 ppt, calculated based on the 3*σ*/*S* method as recommended by IUPAC, with details provided in the SI. According to the data from the U.S. Department of Energy (DOE) in 2024, this ultra-low LOD is significantly below the PAC (Protective Action Criteria) threshold of DCP (0.73 mg m^−3^, 1 ppm),^44^ affirming its applicability for ultra-trace detection and highlighting its potential for effective early warning systems. To benchmark the performance of the BODIQU-*t*BuCZ sensor, its key sensing parameters (LOD, response time, stability, *etc.*) were quantitatively compared with those of previously reported DCP-sensitive materials (Table S5). As summarized in Table S5 and illustrated in Fig. 3b, the BODIQU-*t*BuCZ sensor demonstrates superior overall performance, particularly in achieving both an ultra-low detection limit and ultra-rapid response speed simultaneously, a combination rarely reported in the literature.^41,45–47^ To rigorously evaluate the sensor selectivity and its ability to distinguish diethyl chlorophosphate (DCP) from a diverse range of analytes, including common acidic vapors (HCl and acetic acid), other nerve agent simulants (diethyl cyano phosphate (DCNP), dimethyl methylphosphonate (DMMP), tributyl phosphate (TBP), and triethyl phosphate (TEP)), and the organophosphorus pesticide malathion, we conducted a comprehensive assessment (Fig. 3c and S23). The results demonstrated a clear and highly selective response of the sensor towards DCP. This discrimination was further verified by a two-dimensional principal component analysis (PCA) score plot (Fig. 3d), based on fluorescence response intensity, recovery time, and response time, clearly differentiating DCP from these other analytes. This high selectivity stems from the protonation-based sensing mechanism and the varying acid strengths of the hydrolysis products. DCP hydrolysis yields hydrochloric acid (HCl), a strong acid that efficiently and rapidly protonates the BODIQU core. In contrast, the other tested organophosphorus compounds hydrolyze to form weaker acids, resulting in substantially less fluorophore protonation and consequently diminished fluorescence quenching. These findings highlight the sensor ability to selectively detect DCP with high sensitivity while effectively differentiating it from structurally similar compounds and common acidic interferents, emphasizing its strong potential for practical nerve agent simulant detection.

To investigate the humidity dependence of the sensor, we conducted gradient humidity (RH) tests ranging from 0 to 100% (Fig. S24). The results clearly demonstrate that humidity critically influences both the response and recovery behaviors. Under a dry nitrogen atmosphere, the fluorescence quenching induced by DCP was minimal. In contrast, exposure to DCP in humid air triggered a rapid decrease in fluorescence intensity, with the strongest response observed at around 75% RH. At higher humidity levels, a slight fluorescence recovery was noted, potentially due to changes in DCP partitioning behavior or altered hydrolysis kinetics. The recovery process also exhibited a strong humidity dependence. After exposure to DCP, purging with dry N_2_ resulted in minimal fluorescence recovery. However, switching to humid air led to rapid and complete fluorescence recovery, underscoring the essential role of water vapor in promoting deprotonation and removing adsorbed acidic products (*e.g.*, HCl) from the sensing film. Notably, introducing trace amounts (∼1%) of weakly basic species such as NH_3_ into a 50% RH environment further enhanced the recovery rate. Nevertheless, humidity alone was sufficient to achieve effective sensor regeneration. These findings elucidate the deprotonation mechanism and provide a basis for optimizing the sensor's operating conditions.

Our investigation into the relationship between the protonation mechanism and sensor reversibility reveals a critical concentration-dependent behavior. At low DCP concentrations (<1 ppm), the sensor exhibits complete fluorescence recovery within approximately 600 seconds (Fig. S25). This suggests a dynamic equilibrium where the interaction between the HCl produced by DCP hydrolysis and the BODIQU fluorophore is relatively weak, possibly involving hydrogen bonding or loose ion pairing. These weaker interactions can be readily reversed upon purging with nitrogen or dry air, leading to complete fluorescence recovery. However, at DCP concentrations above 1 ppm, the response becomes increasingly irreversible. This may be attributed to several factors: (1) a higher concentration of HCl could lead to stronger protonation, forming more stable ion pairs that are resistant to deprotonation; (2) secondary reactions, such as irreversible chemical modification of the BODIQU fluorophore, may become more prominent at higher DCP concentrations; or (3) the higher concentration of adsorbed HCl could lead to increased film acidity, further stabilizing the protonated state. Long-term operational stability was evaluated over 50 repeated exposure/recovery cycles at 10 ppb DCP (Fig. 3e), revealing no observable loss in sensing performance, highlighting excellent robustness and recyclability of the sensor film. In contrast to the superior performance of BODIQU-*t*BuCZ, control films fabricated from BODIQU-CZ and BODIQU-BZI exhibited significantly inferior sensitivity and higher detection limits (Fig. S26), conclusively demonstrating the critical role of the bulky *tert*-butyl functionalization in preventing detrimental aggregation-caused quenching, promoting the formation of a porous structure, and thereby enabling the observed ultra-sensitive and rapid solid-state sensing performance.

### Morphological features of BODIQU-*t*BuCZ films

**Fig. 4:**
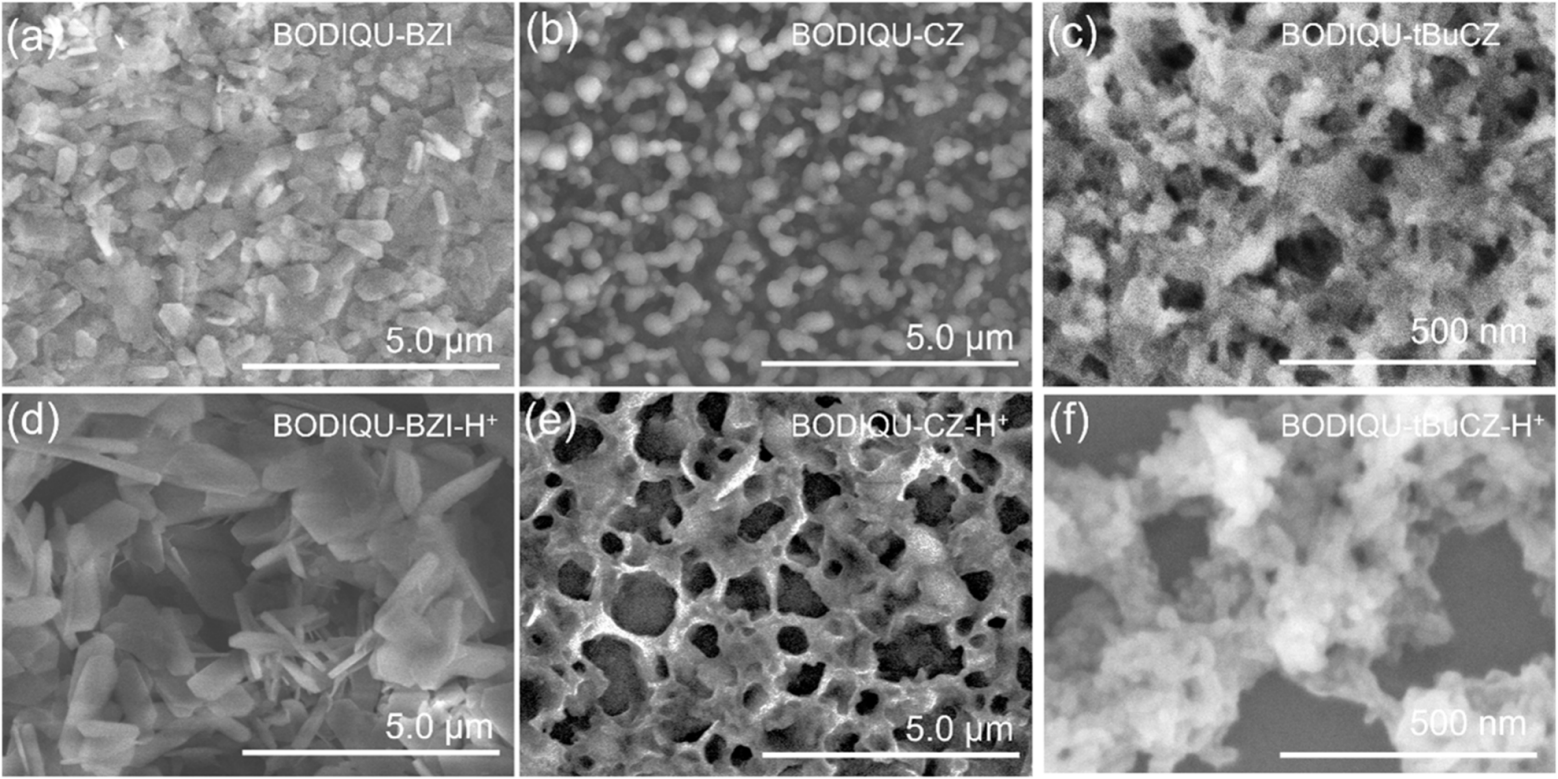
SEM of the films (a) BODIQU-BZI, (b) BODIQU-CZ, (c) BODIQU-*t*BuCZ, (d) BODIQU-BZI-H^+^, (e) BODIQU-CZ-H^+^ and (f) BODIQU-*t*BuCZ-H^+^.

To further rationalize the superior gas-phase sensing performance of BODIQU-*t*BuCZ films, scanning electron microscopy (SEM) and atomic force microscopy (AFM) analyses were employed to investigate the morphological features of BODIQUs (Fig. 4 and S27–S32). In their pristine state, as shown in Fig. 4a–c, the BODIQU-BZI film exhibited large, smooth hexagonal prism-like crystals with minimal porosity, impeding efficient analyte diffusion. The BODIQU-CZ film, while presenting smaller crystals and a rougher surface, remained relatively compact and similarly limited in porosity. In striking contrast, the BODIQU-*t*BuCZ film displayed a three-dimensional network of ellipsoidal cocoon-like nanostructures, characterized by significantly increased porosity. This highly interconnected morphology affords a larger surface area and numerous diffusion pathways, promoting rapid analyte transport and improving the availability of active interaction sites within the sensing film. The hierarchical porous framework thus facilitates both mass transfer and analyte–matrix interactions, aligning with the observed rapid and sensitive gas-phase response. AFM characterization (Fig. S30–S32) revealed distinct morphological features correlating with the observed sensing performance. The BODIQU-*t*BuCZ films exhibited an ultra-thin morphology, with an average thickness of ∼2 nm and a low RMS surface roughness of 0.42 nm. This minimal thinness offers significant advantages by shortening the analyte diffusion path and increasing the effective sensing area-to-volume ratio. This facilitates rapid analyte transport and enhances access to active sensing sites, mitigating diffusion limitations that can hinder sensor response in thicker films. In contrast, control films showed markedly greater thickness and roughness: BODIQU-BZI films had an average thickness of ∼50 nm and an RMS roughness of ∼13 nm, while BODIQU-CZ films averaged ∼100 nm in thickness with an RMS roughness of ∼16 nm. These morphological differences likely contribute to the inferior sensing performance observed for the BODIQU-BZI and BODIQU-CZ films.

To assess structural stability upon exposure to DCP vapor, the three types of films were subjected to 1 ppm DCP under ambient conditions for one hour and subsequently analyzed by SEM (Fig. 4d–f). Pronounced morphological changes were observed in the control materials: the crystalline framework of BODIQU-BZI underwent complete collapse, transforming into dense, lamellar aggregates, while the low-density ellipsoidal structures initially present in BODIQU-CZ disintegrated into ill-defined, continuous 2D mesh-like networks. In sharp contrast, the BODIQU-*t*BuCZ film retained its distinct cocoon-like morphology with minimal distortion, demonstrating remarkable structural resilience under chemical stress.

The formation of this distinct porous microscopic morphology in BODIQU-*t*BuCZ is hypothesized to be closely linked to the strategic incorporation of bulky *tert*-butyl groups. The significant steric hindrance introduced by *tert*-butyl moieties generates strong spatial repulsion effects, which effectively prevent dense molecular packing and notably increase intermolecular distances, thereby promoting pore formation within the film matrix. Moreover, the presence of *tert*-butyl groups may influence molecular alignment during self-assembly processes, particularly during solvent evaporation, likely favoring the development of well-organized network-like architectures. Upon exposure to DCP gas, the expanded molecular spacing and inherent rigidity conferred by the network structure are proposed to effectively alleviate stress concentration, suppress plastic deformation, and act as barriers against excessive adsorption of polar DCP molecules, thus preventing swelling-induced structural degradation observed in the control films.

Collectively, these observations underscore the critical influence of *tert*-butyl functionalization in dictating the hierarchical microstructure and structural resilience of the sensing film. The unique porous and resilient morphology of BODIQU-*t*BuCZ plays a pivotal role in ensuring fast mass transport, high analyte accessibility, and mechanical robustness under operating conditions, features that are conspicuously absent in its non-*t*Bu-substituted counterparts.

### Femtosecond transient absorption (fs-TA) for BODIQU-*t*BuCZ response to DCP

**Fig. 5:**
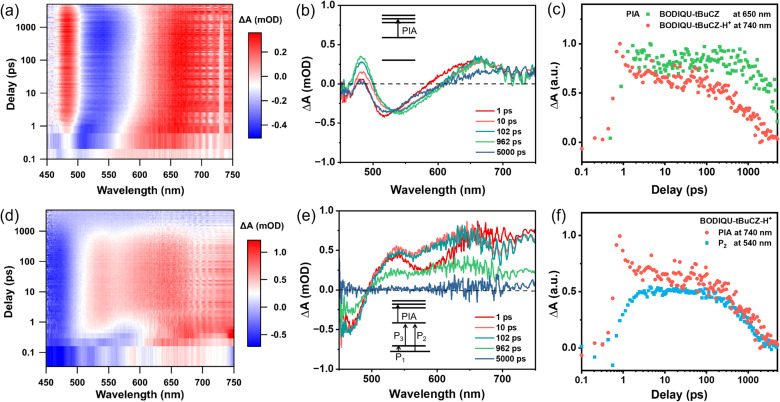
Transient absorption (TA) spectra of BODIQU-*t*BuCZ solution before and after protonation. (a) Pseudo-color TA spectra of BODIQU-*t*BuCZ. (b) TA spectra at different time delays for BODIQU-*t*BuCZ, with insets showing energy levels and optical transitions. (c) Comparison of photo-induced absorption (PIA) peak decay dynamics before and after protonation. (d) Pseudo-color TA spectra of BODIQU-*t*BuCZ-H^+^. (e) TA spectra at different time delays for BODIQU-*t*BuCZ-H^+^, with insets illustrating energy levels and optical transitions in the proposed polaron model. (f) Comparison of P2 and PIA peak decay dynamics in BODIQU-*t*BuCZ-H^+^.

To elucidate the influence of protonation on charge separation dynamics in porous small-molecule systems, femtosecond transient absorption (fs-TA) spectroscopy was conducted on BODIQU-*t*BuCZ, BODIQU-CZ, and BODIQU-BZI, both in their pristine state and after protonation (Fig. 5, S33 and S34). Fig. 5 presents a comparative analysis of time-resolved spectra of BODIQU-*t*BuCZ and its protonated analog, BODIQU-*t*BuCZ-H^+^, within the visible region.

Prior to protonation, photoexcitation of BODIQU-*t*BuCZ in solution resulted in a strong negative photobleaching (PB) signal centered at 530 nm and a broad positive photoinduced absorption (PIA) band at around 660 nm (Fig. 5a and b). The PIA feature arises from the transition of photoexcited electrons to higher unoccupied states, as schematically illustrated in the inset of Fig. 5b. Notably, both the PL and PIA lifetimes of BODIQU-*t*BuCZ are significantly shorter than those of its protonated form, indicating more efficient charge separation in BODIQU-*t*BuCZ-H^+^ (Fig. 5b and c).

Upon protonation, the fs-TA spectra exhibit distinct alterations, characterized by multiple positive features at 520 nm, 650 nm, and 750 nm (Fig. 5d and e). These spectral changes reflect the introduction of additional holes, which disrupt the electrical neutrality of the system and facilitate strong hole-lattice coupling, leading to the formation of hole polarons (inset, Fig. 5e). While the polaron model is traditionally applied to polymers or crystalline semiconductors, it is also applicable to large π-conjugated molecular systems under certain conditions. In the BODIQU-*t*BuCZ molecule, protonation at the central nitrogen induces structural reorganization (Fig. 2f) and lattice interaction, supporting a polaronic picture even in a molecular film. The formation of polarons accounts for the emergence of a dual-peak pattern in the linear absorption spectrum (Fig. S12), the redshifted and quenched PL emission, and the multi-peak signatures observed in the TA profiles of BODIQU-*t*BuCZ-H^+^.

As illustrated in Fig. 5e, the hole polaron enables three optically allowed transitions—P_1_, P_2_, and P_3_. While P_1_ likely lies in the mid-infrared region beyond the spectral range of the current instrumentation, transitions P_2_ and P_3_ occur in the visible region and correspond to the twin absorption peaks observed in the steady-state absorption spectrum (Fig. S12). Following photoexcitation, P_3_ is suppressed due to electron depletion at the HOMO, limiting available electrons for the P_3_ transition. Meanwhile, a redshifted PIA feature appears at 740 nm immediately after excitation, consistent with the initial excitation of the polaronic system. Subsequently, as electron depletion progresses at the LUMO, the P_2_ transition is enhanced. This temporal evolution is clearly captured in Fig. 5f, where P_2_ intensifies gradually while the PIA signal rapidly decays within the first 5 ps. Such kinetic behavior is inconsistent with conventional excited-state absorption of neutral or protonated fluorophores. Instead, it aligns with dynamic polaronic transitions involving HOMO and LUMO depletion, ruling out a general excited-state absorption explanation.

Comparisons of the fs-TA data with those of BODIQU-CZ and BODIQU-BZI further support the unique properties of BODIQU-*t*BuCZ. While BODIQU-CZ and BODIQU-BZI also exhibit changes in TA spectra upon protonation, their features and dynamics differ from those of BODIQU-*t*BuCZ. Furthermore, and significantly, the pristine solid films of BODIQU-CZ and BODIQU-BZI show evidence of faster excited-state decay kinetics compared to BODIQU-*t*BuCZ films, consistent with the greater solid-state fluorescence quenching observed in these control materials (Fig. S22) due to aggregation. This contrasts with the slower decay of neutral BODIQU-*t*BuCZ films, which is attributed to the effective suppression of aggregation by the *tert*-butyl groups.

In summary, fs-TA spectroscopy provides compelling ultrafast dynamic evidence supporting the proposed protonation-induced fluorescence quenching mechanism. Protonation leads to a fundamental reorganization of the electronic structure, resulting in the formation of transient absorbing species (likely polaronic) that open up highly efficient, rapid non-radiative decay pathways on the picosecond timescale. This fast deactivation process directly explains the observed fluorescence quenching upon DCP exposure. Moreover, the fs-TA comparison with control compounds highlights how the molecular design in BODIQU-*t*BuCZ influences both initial solid-state photophysics (suppressing aggregation-induced quenching) and the dynamics of the protonated state, contributing to the overall high sensitivity and rapid response speed of the sensor. In addition, the observed strong PL quenching upon protonation is consistent with exciton–polaron interaction—a well-known non-radiative decay pathway in molecular and polymeric optoelectronic systems.^48^

## Conclusions

A high-performance fluorescent sensor based on a boron difluoride complex (aza-BODIQU) functionalized with a 3,6-di-*tert*-butylcarbazole moiety was successfully constructed for neurotoxic agent detection. The resulting BODIQU-*t*BuCZ film exhibited a highly porous structure with a remarkable 38.76% free volume. This porosity, stemming from the steric hindrance induced by the *tert*-butyl groups, effectively suppresses detrimental π–π stacking and facilitates rapid analyte mass transfer, addressing a fundamental challenge in solid-state vapor sensing. This unique porous architecture, coupled with the molecule's intrinsic reactivity towards electrophiles, enabled ultra-trace detection of DCP vapor with exceptional performance metrics: a detection limit of 0.001 ppt, a rapid 3 s response time, and excellent recyclability over >50 exposure/recovery cycles. Quantitative performance comparisons revealed its superiority over previously reported sensors. In contrast, control materials lacking the *tert*-butyl functionalization exhibited significantly inferior performance, attributed to their denser, less porous morphologies and more pronounced aggregation. Furthermore, the BODIQU-*t*BuCZ sensor demonstrated high selectivity against common acidic interferents and excellent photostability. Collectively, these findings establish a robust steric design strategy for constructing porous fluorescent sensors that combine high solid-state efficiency with real-world applicability. This approach offers a promising platform for the future development of versatile, scalable, and field-ready sensing technologies targeting a broader range of hazardous analytes.

## Author contributions

Y. Liu, M. Qiao and J. Liu contributed equally to this work. X. Zhu led the project conceptualization. Y. Liu, X. Zhu and G. Wang performed the synthetic work. Y. Liu and M. Qiao conducted steady-state photophysical measurements, while J. Liu and Y. Zhai carried out femtosecond transient absorption spectroscopy (fs-TAS) experiments. R. Wen designed the home-made sensing platform. Y. Liu and X. Zhu drafted the initial manuscript. S. Wang co-designed the graphical content, including reaction schematics and the graphical abstract. X. Zhu, Y. Zhai, L. Ding, and Y. Fang critically revised the manuscript and supervised the project. All authors participated in editing, reviewing, and approving the final version of the manuscript.

## Conflicts of interest

There are no conflicts to declare.

## Supplementary Material

SC-OLF-D5SC05184C-s001

## Data Availability

The data supporting this article have been included as part of the SI. See DOI: https://doi.org/10.1039/d5sc05184c.

## References

[cit1] Stone R. (2018). How to defeat a nerve agent. Science.

[cit2] Sydnes L. K. (2020). Nerve agents: from discovery to deterrence. Nature.

[cit3] Yao M., Zhou R., Yuan M., Wang H., Wang L., Sun H., Fu Y., Xiao R., Wang H., Wang G., Zhu M. (2023). Multifunctional semiconducting fibers for visual detection of sarin gas. Adv. Fiber Mater..

[cit4] Kumar V. (2021). Chromo-fluorogenic sensors for chemical warfare agents in real-time analysis: journey towards accurate detection and differentiation. Chem. Commun..

[cit5] Steindl D., Boehmerle W., Körner R., Praeger D., Haug M., Nee J., Schreiber A., Scheibe F., Demin K., Jacoby P., Tauber R., Hartwig S., Endres M., Eckardt K. U., Jacoby P. (2021). Novichok nerve agent poisoning. Lancet.

[cit6] Stone R. (2018). U.K. attack puts nerve agent in the spotlight. Science.

[cit7] Chen Q., Liu J., Liu S., Zhang J., He L., Liu R., Jiang H., Han X., Zhang K. (2023). Visual and rapid detection of nerve agent mimics in gas and solution phase by a simple fluorescent probe. Anal. Chem..

[cit8] Stone R. (2019). Obscure cold war nerve agents set to be banned. Science.

[cit9] Lyu C., Zhao C., Wang M., Li J., Cai Z., Dou X., Zu B. (2025). Exactly restricting the phenyl ring rotation in metal-organic framework for ultra-sensitive and specific ratiometric fluorescent sensing of sarin. Aggregate.

[cit10] Abbas Z., Yadav U., Butcher R. J., Patra A. (2021). Luminescent heteroleptic Eu(III) probes for the selective detection of diethyl chlorophosphate as a G-series nerve agent mimic in the vapor phase using solid-state films. J. Mater. Chem. C.

[cit11] Rakow N. A., Suslick K. S. (2000). A colorimetric sensor array for odour visualization. Nature.

[cit12] Pan Y., Qin M., Wang P., Yang L., Zhang L., Yan C., Zhang C., Wang W. (2022). Interface and sensitive characteristics of the viscoelastic film used in a surface acoustic wave gas sensor. ACS Sens..

[cit13] Nguyen V. P., Qian W., Li Y., Liu B., Aaberg M., Henry J., Zhang W., Wang X., Paulus Y. M. (2021). Chain-like gold nanoparticle clusters for multimodal photoacoustic microscopy and optical coherence tomography enhanced molecular imaging. Nat. Commun..

[cit14] Rodrigues J., Amin A., Chandra S., Mulla N. J., Nayak G. S., Rai S., Ray S., Mahato K. K. (2024). Machine learning enabled photoacoustic spectroscopy for noninvasive assessment of breast tumor progression in vivo: a preclinical study. ACS Sens..

[cit15] Raghushaker C. R., Rodrigues J., Nayak S. G., Ray S., Urala A. S., Satyamoorthy K., Mahato K. K. (2021). Fluorescence and photoacoustic spectroscopy-based assessment of mitochondrial dysfunction in oral cancer together with machine learning: a pilot study. Anal. Chem..

[cit16] Nguyen P. Q., Soenksen L. R., Donghia N. M., Angenent-Mari N. M., de Puig H., Huang A., Lee R., Slomovic S., Galbersanini T., Lansberry G. (2021). Wearable materials with embedded synthetic biology sensors for biomolecule detection. Nat. Biotechnol..

[cit17] Gupta R. D., Goldsmith M., Ashani Y., Simo Y., Mullokandov G., Bar H., Ben-David M., Leader H., Margalit R., Silman I., Sussman J. L., Tawfik D. S. (2011). Directed evolution of hydrolases for prevention of G-type nerve agent intoxication. Nat. Chem. Biol..

[cit18] Khan M. A. K., Long Y. T., Schatte G., Kraatz H. B. (2007). Surface studies of aminoferrocene derivatives on gold: electrochemical sensors for chemical warfare agents. Anal. Chem..

[cit19] Williams D., Pappas G. (2015). Rapid identification of nerve agents Sarin (GB) and Soman (GD) with the use of a field-portable GC/SAW vapor detector and liquid desorption front-end device. Field Anal. Chem. Technol..

[cit20] Hill H. H., Martin S. J. (2002). Conventional analytical methods for chemical warfare agents. Pure Appl. Chem..

[cit21] Zang R., Liu Y., Wang Y., Feng L., Ge Y., Qin M., Du Y., Ning J., Ma X., Dou X. (2025). Defect engineering Zr-MOF-endowed activity-dimension dual-sieving strategy for anti-acid recognition of real phosphoryl fluoride nerve agents. Adv. Funct. Mater..

[cit22] Huang R., Liu T., Peng H., Liu J., Liu X., Ding L., Fang Y. (2024). Molecular design and architectonics towards film-based fluorescent sensing. Chem. Soc. Rev..

[cit23] Ding N., Liu T., Peng H., Liu J., Ding L., Fang Y. (2023). Film-based fluorescent sensors: from sensing materials to hardware structures. Sci. Bull..

[cit24] Peng H., Ding L., Fang Y. (2024). Recent advances in construction strategies for fluorescence sensing films. J. Phys. Chem. Lett..

[cit25] Dagnaw F. W., Feng W., Song Q.-H. (2020). Selective and rapid detection of nerve agent simulants by polymer fibers with a fluorescent chemosensor in gas phase. Sens. Actuators, B.

[cit26] Dale T. J., Rebek Jr J. J. A. C. (2009). Hydroxy oximes as organophosphorus nerve agent sensors. Angew. Chem., Int. Ed..

[cit27] Zhou X., Zeng Y. Y., Chen L. Y., Wu X., Yoon Y. (2016). A fluorescent sensor for dual-channel discrimination between phosgene and a nerve-gas mimic. Angew. Chem., Int. Ed..

[cit28] Ji X., Tian W. G., Jin K. F., Diao H. L., Huang X., Song G. J., Zhang J. (2022). Anionic polymerization of nonaromatic maleimide to achieve full-color nonconventional luminescence. Nat. Commun..

[cit29] Liu L., Li S., Luo W., Yao J., Liu T., Qin M., Huang Z., Ding L., Fang Y. (2024). Compact device prototype for turn-on fluorescence detection of sarin based on reactive 4,4-diaryloxy-BODIPY derivatives. Sens. Diagn..

[cit30] Cai Y., Li C., Song Q. (2017). Fluorescent chemosensors with varying degrees of intramolecular charge transfer for detection of a nerve agent mimic in solutions and in vapor. ACS Sens..

[cit31] Ding N., Liu T., Peng H., Liu J., Ding L., Fang Y. (2023). Film-based fluorescent sensors: from sensing materials to hardware structures. Sci. Bull..

[cit32] Ren L. Q., Zhan B., Zhao J., Guo Y., Zu B., Li Y., He C. (2025). Modular enantioselective assembly of multi-substituted boron-stereogenic BODIPYs. Nat. Chem..

[cit33] Turksoy A., Yildiz D., Akkaya E. U. (2019). Photosensitization and controlled photosensitization with BODIPY dyes. Coord. Chem. Rev..

[cit34] Poddar M., Misra R. (2020). Recent advances of BODIPY based derivatives for optoelectronic applications. Coord. Chem. Rev..

[cit35] Zhu X., Liu R., Li Y., Huang H., Wang Q., Wang D., Zhu X., Liu S., Zhu H. (2014). An AIE-active boron-difluoride complex: multi-stimuli-responsive fluorescence and application in data security protection. Chem. Commun..

[cit36] Shao Y., Jia W., Qin G., Jiang X., Xu Z. (2025). Carboxyl-functionalized BODIPY and Aza-BODIPY dyes: Synthetic strategies and emerging applications. Coord. Chem. Rev..

[cit37] Zhu X., Huang H., Liu R., Jin X., Li Y., Wang D., Wang Q., Zhu H. (2015). Aza-boron-diquinomethene complexes bearing N-aryl chromophores: synthesis, crystal structures, tunable photophysics, the protonation effect and their application as pH sensors. J. Mater. Chem. C.

[cit38] Wang D., Wang X., Zhou S., Gu P., Zhu X., Wang C., Zhang Q. (2023). Evolution of BODIPY as triplet photosensitizers from homogeneous to heterogeneous: the strategies of functionalization to various forms and their recent applications. Coord. Chem. Rev..

[cit39] Tsyshevsky R., McEntee M., Durke E. M., Karwacki C., Kuklja M. M. (2020). Degradation of fatal toxic nerve agents on dry TiO_2_. ACS Appl. Mater. Interfaces.

[cit40] Fan S., Loch A. S., Vongsanga K., Dennison G. H., Burn P. L., Gentle I. R., Shaw P. E. (2024). Differentiating between V- and G-series nerve agent and simulant vapours using fluorescent film responses. Small Methods.

[cit41] Zhou Z., Zhang L., Peng L., Li Y., Zhu X., Wu Y., Qiu Z., He G., Qin M., Peng H., Fang Y. (2024). Dynamic response and discrimination of gaseous sarin using a boron-difluoride complex film-based fluorescence sensor. Aggregate.

[cit42] Fang M., Yang J., Li Z. (2022). Light emission of organic luminogens: Generation, mechanism and application. Prog. Mater. Sci..

[cit43] Qiu Z., Xiao Y., Zhang L., Miao Y., Zhang B., Zhu X., Ding L., Peng H., Fang Y. (2024). Highly sensitive and selective detection of DCP vapors using pyridine-based fluorescent nanofilms. Chem. Commun..

[cit44] U.S. Department of Energy (DOE) , Chemical Safety Standards, [Online], 2023, available at: https://www.doe.gov/documents/chemical-safety-standards, accessed: 15 July 2024

[cit45] Zhu R., Azzarelli J. M., Swager T. M. (2016). Wireless hazard badges to detect nerve-agent simulants. Angew. Chem., Int. Ed..

[cit46] Liu K., Qin M., Shi Q., Wang G., Zhang J., Ding N., Xi H., Liu T., Kong J., Fang Y. (2022). Fast and selective detection of trace chemical warfare agents enabled by an ESIPT-based fluorescent film sensor. Anal. Chem..

[cit47] Mo W., Zhu Z., Kong F., Li X., Chen Y., Liu H., Cheng Z., Ma H., Li B. (2022). Controllable synthesis of conjugated
microporous polymer films for ultrasensitive detection of chemical warfare agents. Nat. Commun..

[cit48] van Reenen S., Vitorino M. V., Meskers S. C. J., Janssen R. A. J., Kemerink M. (2014). Photoluminescence quenching in films of conjugated polymers by electrochemical doping. Phys. Rev. B: Condens. Matter Mater. Phys..

