# Supplementary material for: Steric hindrance-engineered porous fluorescent films for ultrafast and ultrasensitive detection of nerve agent simulants
Source: Chem Sci. 2025 Aug 18;16(36):16924–35. doi: 10.1039/d5sc05184c (PMC12378549; doi:10.1039/d5sc05184c)
Supplement: SC-016-D5SC05184C-s001 [file SC-016-D5SC05184C-s001.pdf]

Supplementary Information (SI) for Chemical Science.
This journal is © The Royal Society of Chemistry 2025

Supporting Information

Steric hindrance-engineered porous boron difluoride complex films for ultrasensitive fluorescent detection of neurotoxic agents

Yuxuan Liu,†[a] Min Qiao,†[a] Jiali Liu,†[b] Gege Wang,[a] Siyue Wang,[a] Ruijuan Wen,[a] Yaxin Zhai,*[b] Liping Ding,*[a] Xiaolin Zhu,*[a] Yu Fang[a]

a. Shaanxi Key Laboratory of New Concept Sensors and Molecular Materials, Key Laboratory of Applied Surface and Colloid Chemistry (Ministry of Education), School of Chemistry and Chemical Engineering, Shaanxi Normal University, Xi'an 710119, P.R. China. E-mail: xiaolinchem@snnu.edu.cn; dinglp33@snnu.edu.cn

b. Key Laboratory of Low-Dimensional Quantum Structures and Quantum Control of Ministry of Education, Department of Physics, Hunan Normal University, Changsha 410081, P.R. China. E-mail: yzhai@hunnu.edu.cn

† These authors contributed equally to this work.

E-mail: xiaolinchem@snnu.edu.cn (Prof. X. Zhu); dinglp33@snnu.edu.cn (Prof. L. Ding); yzhai@hunnu.edu.cn (Prof. Y. Zhai)

Contents

1. Synthetic Procedures

2. Instruments

3. Crystallographic Characterization

4. Energy Level

5. Photophysical Properties

6. Response for BODIQU Derivatives to DCP Detection

7. Limit of Detection (LOD) Calculation

8. Sensing Performance of BODIQU Film

9. Morphology Data

10. TA Data

11. Temperature-Dependent Current-Voltage (I-V) Characterization

12. NMR and MS Characterization Data

13. References

1. Synthetic Procedures

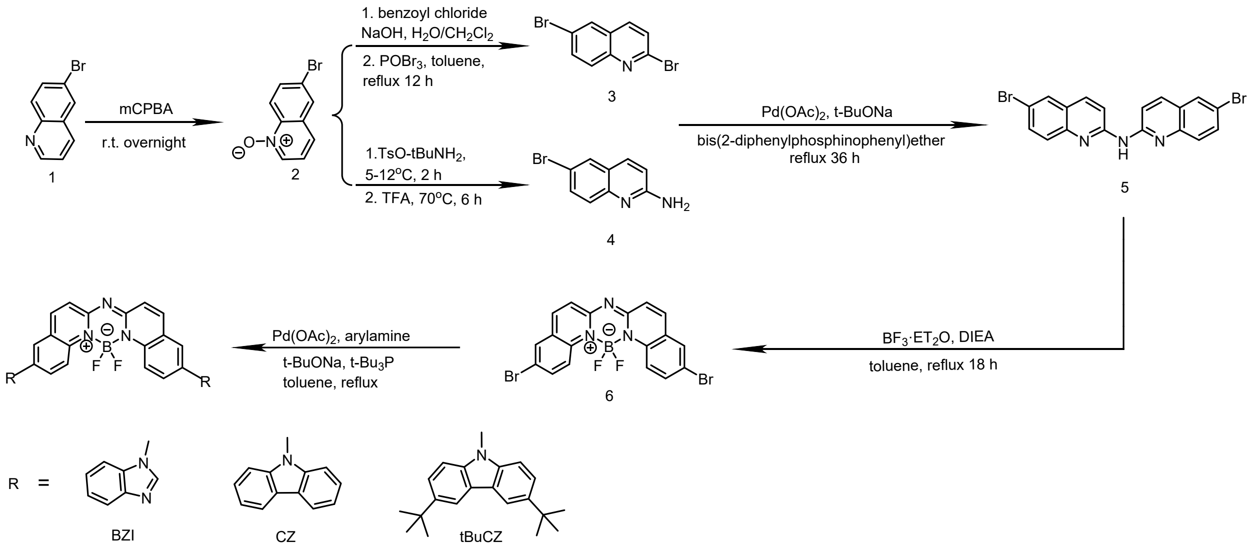

Scheme S1. Synthesis Routes of Difluoro-boron Complex.

BrQNBF (difluoro-boron complex of bis(6-bromoquinolin-2-yl)amine) was synthesized according to our previous report[1].

Synthesis of 6-bromoquinoline N-oxide (2)

M-chloroperoxybenzoic acid (m-CPBA) (5.07 g, 29.4 mmol) was added slowly to a CH_2Cl_2 solution of 1 (6.00 g, 26.7 mmol) at r.t., then stirred overnight. The reaction was filtered and the filtrate was treated with saturated $NaHCO_3$ solution until no CO_2 gas yield. Then, adjusted pH = 10 with 3 M NaOH aq. solution and extracted with CH_2Cl_2 20 mL three times. The solvent was removed under reduced pressure. The crude product was then purified by silica gel column chromatography (2% methanol/CH_2Cl_2). Pale yellow solid was afforded (4.45 g, 87% yield).

Synthesis of 2,6-dibromoquinoline (3)

To a round bottom flask was charged 6-bromoquinoline N-oxide (**2**) (1.27 g, 5.7 mmol) and sodium hydroxide (0.44 g, 11.0 mmol) in water (12.0 mL) and CH_2Cl_2 (6.0 mL), benzoyl chloride (8.5 mmol, 1.0 mL) was added slowly to the vigorously stirred mixture. A reflux was observed when the addition was nearly completed. Then the flask was then cooled to 5 °C with ice-bath, and addition was resumed. After 1 h stirring, the precipitate was filtered off, rinsed well with water (25 mL) and CH_2Cl_2 (25 mL) respectively, then dried in the air, white product was obtained.

To a re-sealable Schlenk tube was charged the white product, $POBr_3$ and dry toluene as the solvent under N_2, heated to reflux overnight. After cooling to r.t., the mixture was poured on ice,

washed with saturated $NaHCO_3$ and extracted with CH_2Cl_2 several times. Remove the solvent under reduced pressure. Off-white solid was afforded (1.1 g, 70% yield).

Synthesis of 6-bromoquinolin-2-amine (4)

To a round bottom flask was charged **2** (2.24 g, 10 mmol, 1.0 equiv), 20 mL trifluorotoluene and 10 mL chloroform under N_2. After compound **2** was dissolved, the mixture was cooled to 0°C with an ice-bath. T-butylamine (5.6 mL, 50.0 mmol) was added followed by Ts_2O (6.5 g, 20.0 mmol) while the temperature was kept at 5 °C. The reaction was then treated with TFA 25 mL at 70 °C for 24 h. Most of the solvents could be removed under vacuum. The oil residue was diluted with CH_2Cl_2 and quenched with 50% aq. solution NaOH to pH = 10. The aqueous layer was extracted with CH_2Cl_2 (50 mL) three times. The combined organic layers were dried with $MgSO_4$. The solvent was removed by reduced pressure and the crude product was then purified by silica gel column chromatography (2% MeOH/CH_2Cl_2) to give the desired 4 as a gray solid (1.6 g, 71.4% yield).

Synthesis of bis(6-bromoquinolin-2-yl)amine (5)

To a re-sealable Schlenk tube was charged with bis(2-diphenylphosphinophenyl) ether (88 mg, 0.16 mmol, 4% mmol), **4** (892 mg, 4 mmol), 2,6-diboromoquinoline **3** (1140 mg, 4.0 mmol), t-BuONa (536 mg, 5.6 mmol), and degassed dry toluene (30 mL). The Schlenk tube was capped and carefully subjected to three cycles of evacuation-backfilling with N_2. Finally, $Pd(OAc)_2$ (36 mg, 0.16 mmol, 4% mmol) was added. It was then sealed and immersed into a 110 °C oil bath. After 5 h reflux, the mixture was cooled to r.t., diluted with THF and ethyl ether, filtered, concentrated, and purified by silica gel column chromatography (2% MeOH/CH_2Cl_2) to afford the product as white solid. (1.05 g, 69% yield)

Synthesis of difluoro-boron complex of bis(6-bromoquinolin-2-yl)amine (6)

To a three-necked round bottom flask was charged 5 (1.40 g, 3.3 mmol) and dry toluene under N_2. DIEA (1.6 mL, 9.9 mmol) was slowly injected to the toluene solution. After 10 min stirring, $BF_3{\cdot}Et_2O$ (3.5 mL,13.2 mmol) was injected dropwise to the solution. The reaction was then refluxed overnight. After cooling to room temperature, the precipitate was filtered off and dried in air for 2 h. The precipitate was added into 100 mL water and stirred for 30 min, adjusted pH to 7 with saturated $NaHCO_3$. Filtrated and dried in the air for 2 h, washed with diethyl ether and

isopropyl ether respectively to give the crude product. It was then purified by silica column chromatography (CH_2Cl_2) to afford 7 as a yellow solid (1.27 g, 80.6% yield)

Synthesis of BODIQU-BZI, BODIQU-CZ, BODIQU-tBuCZ

To a flask was charged BODIQU-Br (200 mg, 0.4 mmol), arylamines (1.2 mmol), t-BuONa (100 mg, 1.2 mmol), $Pd(OAc)_2$ (12 mg, 0.05 mmol, 5% mol), t-Bu_3P (0.05 mmol) and 6 mL toluene. The reaction mixture was refluxed under N_2 for 12 h. After cooling to r.t., it was diluted with brine (20 mL) and extracted with CH_2Cl_2. The organic layer was then dried with Na_2SO_4 and the solvent was removed under reduced pressure. The product was purified by silica gel column chromatography (50% PE/CH_2Cl_2).

Difluoro-boron complex of BODIQ-BZI

60 mg of yellow solid was obtained, yield 37%. ^{1}H NMR (600 MHz, Chloroform-*d*) δ 8.48 (d, J = 9.6 Hz, 4H), 7.93(d, J = 9.2 Hz, 4H), 7.84 (d, J = 2 Hz, 4H), 7.80 (d, J = 2.4 Hz, 2H),7.78 (d, J = 2.4 Hz, 2H), 7.21 (d, J = 8.8Hz, 4H). ^{13}C NMR (151 MHz, Chloroform-*d*) δ 146.72, 145.67, 137.55, 131.82, 127.29, 124.21, 122.73, 117.27, 115.38, 114.63, 113.49, 111.61.

Difluoro-boron complex of BODIQ-CZ

80 mg of yellow solid was obtained, yield 38%. ^{1}H NMR (600 MHz, Chloroform-*d*) δ 8.91–8.88 (m, 2H), 8.18 (d, J = 7.7 Hz, 4H), 8.09 (d, J = 9.1 Hz, 2H), 7.97 (dd, J = 9.2, 2.4 Hz, 2H), 7.92 (d, J = 2.3 Hz, 2H), 7.52–7.40 (m, 8H), 7.40-7.29 (m, 6H). ^{13}C NMR (151 MHz, Chloroform-*d*) δ 146.42, 145.61, 137.47, 135.77, 131.66, 127.12, 126.58, 124.01, 122.64, 120.76, 117.07, 115.18, 114.18, 113.29, 111.41. HRMS (ESI) calcd. for $C_{42}H_{27}BF_2N_5^+$ $[M+H]^+$ = 650.2329, found: 650.2330.

Difluoro-boron complex of BODIQ-tBuCZ

80 mg of yellow solid was obtained, yield 40%. ^{1}H NMR (600 MHz, Chloroform-*d*) δ 8.87 (d, J = 9.2 Hz, 1H), 8.18 (d, J = 1.9 Hz, 2H), 8.09 (d, J = 9.1 Hz, 1H), 7.97 (dd, J = 9.2, 2.5 Hz, 1H), 7.92 (d, J = 2.5 Hz, 1H), 7.50 (dd, J = 8.6, 1.9 Hz, 2H), 7.43 (d, J = 8.7 Hz, 2H), 7.34 (d, J = 9.1 Hz, 1H), 1.49 (s, 18H). ^{13}C NMR (151 MHz, Chloroform-*d*) δ 143.50, 139.01, 125.05, 123.83, 123.66, 116.42, 108.91, 34.75, 31.95. HRMS (ESI) calcd. for $C_{58}H_{58}BF_2N_5^+$ $[M+H]^+$ = 874.4836, found: 874.4837.

2. Instruments

Unless otherwise noted, all reagents were purchased from commercial suppliers. Reactions were performed under a dry N_2 atmosphere using a standard vacuum line technique. All solvents used in the reactions were dehydrated and distilled in advance. The compounds were fully characterized by ^{1}H NMR. ^{1}H NMR spectra were obtained on Bruker AV 600 NMR spectrometer. The mass spectrometry data were measured on Bruker maxis UHR-TOF mass spectrometer in ESI positive mode. UV-vis absorption spectra were obtained on a U-3900 (Hitachi) spectrophotometer. The fluorescence measurements were performed on a time-correlated single-photon counting Edinburgh FLS980 fluorescence spectrometer with a xenon lamp as the light source at 298 K. The quantum yields of films were obtained by quantum efficiency measurement system (Japan Hamamatsu, C9920-02G). Powder X-ray diffraction (PXRD) measurements were performed on a Bruker D8 Advance diffractometer. Grazing-incidence wide-angle X-ray scattering (GIWAXS) measurements were conducted using an Anton Paar SAXSpoint 2.0 system. The temperature-dependent current-voltage (I-V) characteristics were measured using a micro-probe station (HX-002).

3. Crystallographic Characterization

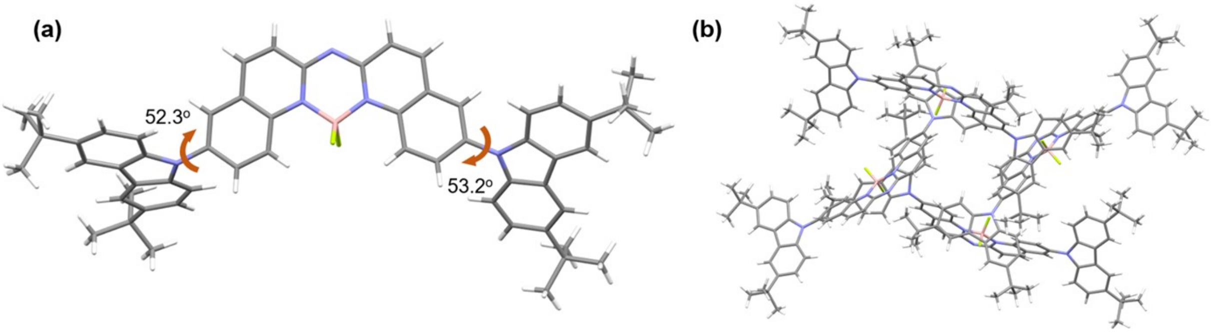

Figure S1. (a) Single crystal structure of **BODIQU-tBuCZ**, (b) molecular packing of **BODIQU-tBuCZ** (CCDC 1021584).

Table S1. Crystal data and structure refinement for **BODIQU-tBuCZ**

Compound	**BODIQU-tBuCZ**
CCDC number	1021584
Empirical formula	$C_{58}H_{58}BF_2N_5$
Formula weight	873.90
Temperature/K	273
Crystal system	Monoclinic
Space group	$P2_{1/c}$
a/Å	22.4457(17)
b/Å	11.3035(7)
c/Å	20.0181(14)
α/°	90.00
β/°	96.623(5)
γ/°	90.00
Volume/Å^3	5073.6(6)
Z	4
ρ_{calc}/g/cm^3	1.144
μ/mm^{-1}	0.564
F(000)	1856.0
Crystal size/mm^3	0.28 × 0.26 × 0.11
Radiation	CuK\a (λ=1.54178)
2θ range for data collection/°	3.94 to 66.09
Index ranges	-26 ≤ h ≤ 26, -11 ≤ k ≤ 13, -23 ≤ l ≤ 22
Reflections collected	8338
Independent reflections	5352 [R_{int} = 0.0535, R_{sigma} = 0.0542]
Goodness-of-fit on F^2	1.191
Final R indexes [I>=2σ(I)]	R_1=0.1316, wR_2=0.2674
Final R indexes [all data]	R_1=0.1875 wR_2=0.2800
Largest diff. peak/hole / e Å^{-3}	0.23/-0.25

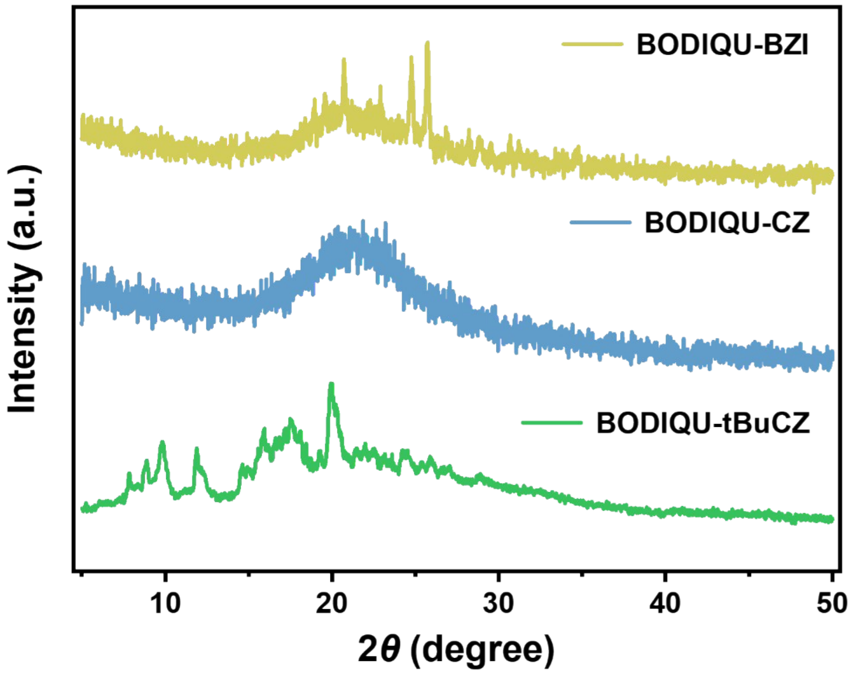

Figure S2. PXRD of the **BODIQU-BZI**, **BODIQU-CZ**, **BODIQU-tBuCZ**.

Notes: The PXRD samples were prepared by grinding the compounds into fine powders.

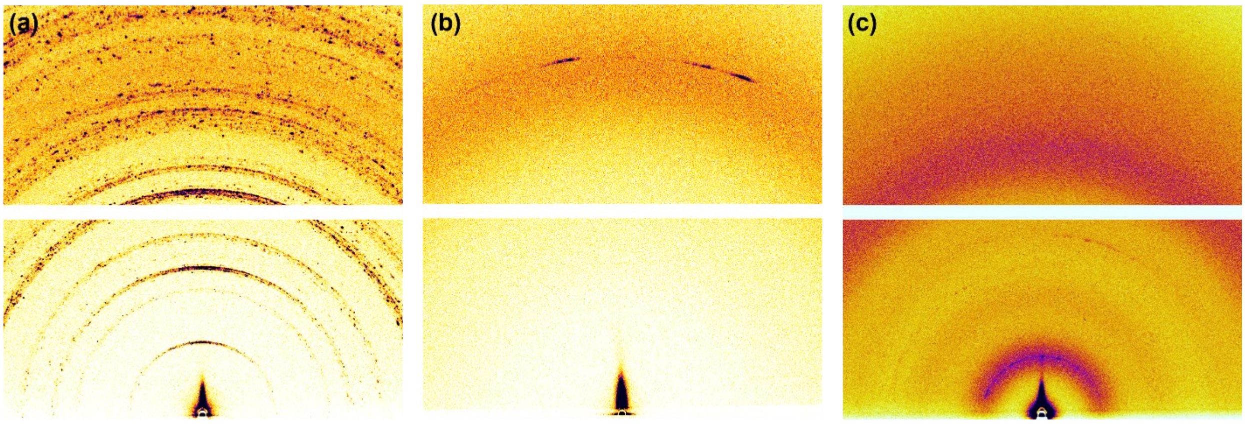

Figure S3. GIWAXS of the **BODIQU-BZI** (a), **BODIQU-CZ** (b), **BODIQU-tBuCZ** (c) films.

Notes: The samples for experimental measurements were prepared by dissolving the 20 mg compounds in 1 mL dichloromethane and drop-casting 20 μL of the solution onto 1×1 cm quartz substrates.

4. Photophysical Properties

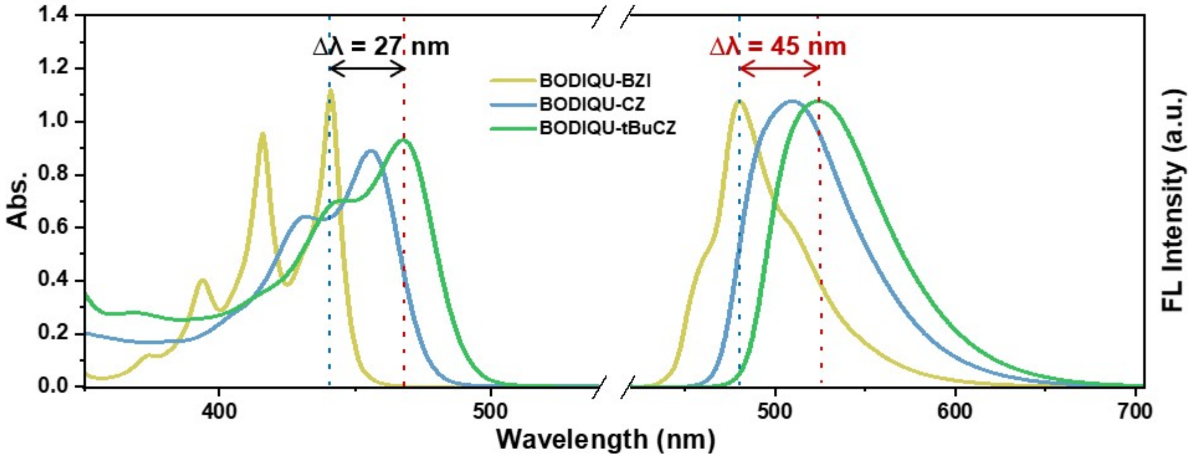

Figure S4. The UV-vis absorption and fluorescence emission spectra of **BODIQU-BZI**, **BODIQU-CZ**, **BODIQU-tBuCZ** in DCM (5×10^{-5} M).

Figure S4 demonstrates the Stokes shifts exhibit a gradual enhancement trend across the three compounds: **BODIQU-BZI**, **BODIQU-CZ**, and **BODIQU-tBuCZ**.

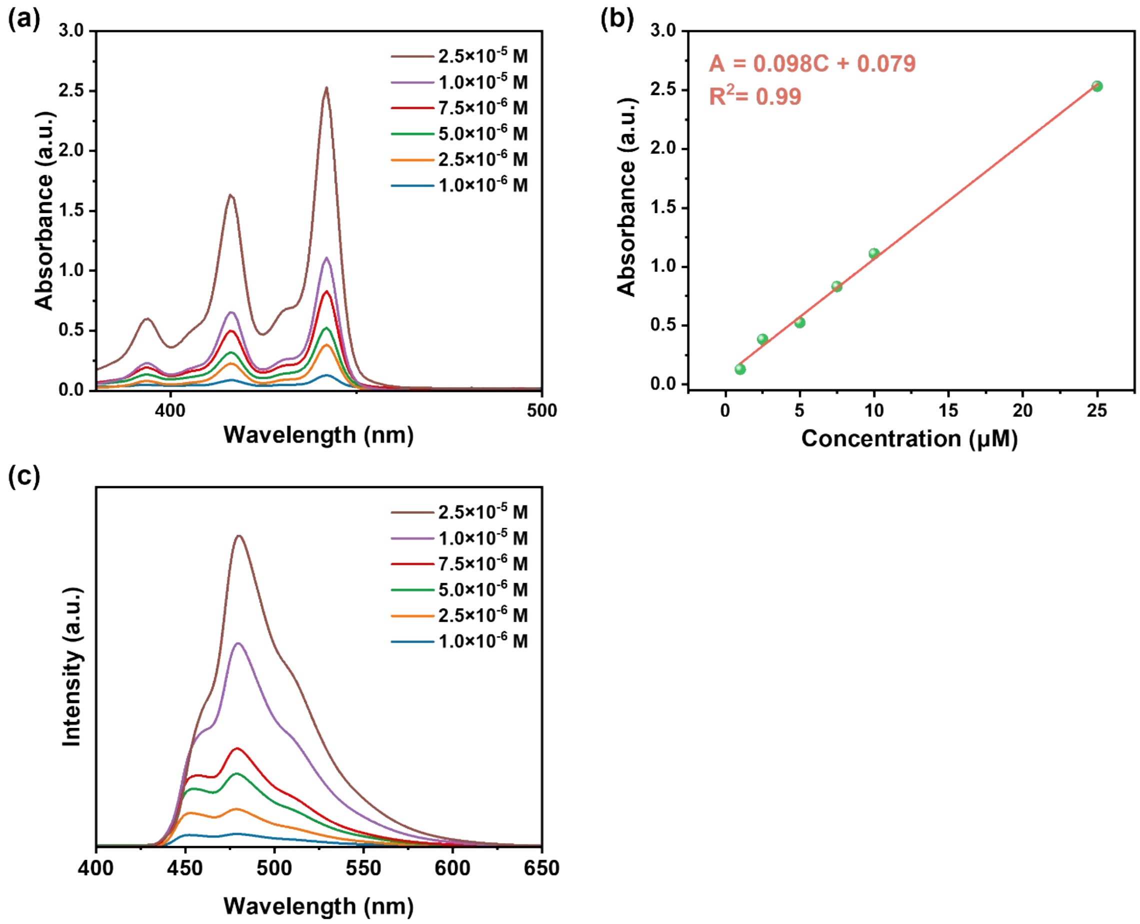

Figure S5. The UV-vis absorption spectra (a) of **BODIQU-BZI** in DCM with different concentrations varied from 1 μM to 25 μM. (b) Plots of the absorbance (A) recorded at 460 nm, against the compound concentration (*c*) (ε = 98000 L·mol^{-1}·cm^{-1}). The fluorescence emission spectra (c) of **BODIQU-BZI** in DCM at room temperature with different concentrations. The excitation wavelength was 380 nm.

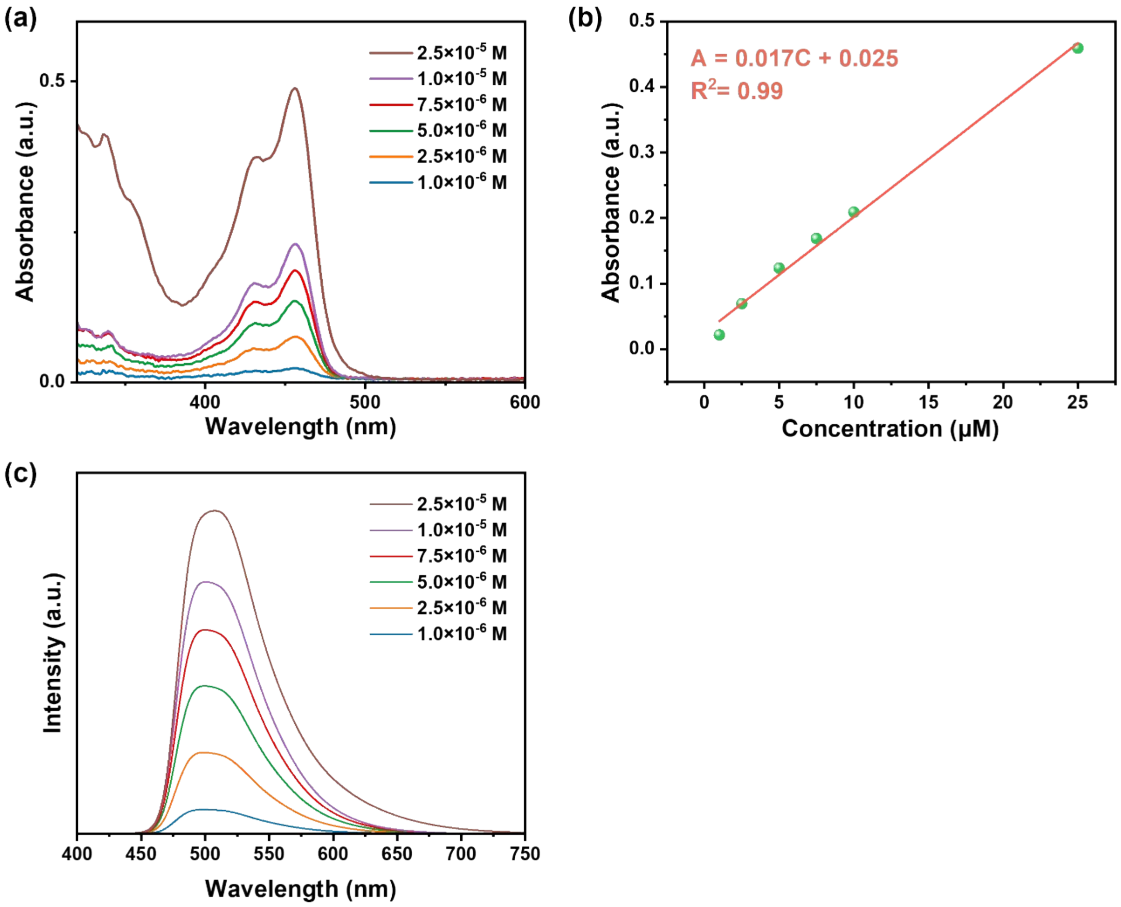

Figure S6. The UV-vis absorption spectra (a) of **BODIQU-CZ** in DCM with different concentrations varied from 1 μM to 0.1 mM. (b) Plots of the absorbance (A) recorded at 460 nm, against the compound concentration (*c*) (ε = 17000 L·mol^{-1}·cm^{-1}). The fluorescence emission spectra (c) of **BODIQU-CZ** in DCM at room temperature with different concentrations. The excitation wavelength was 380 nm.

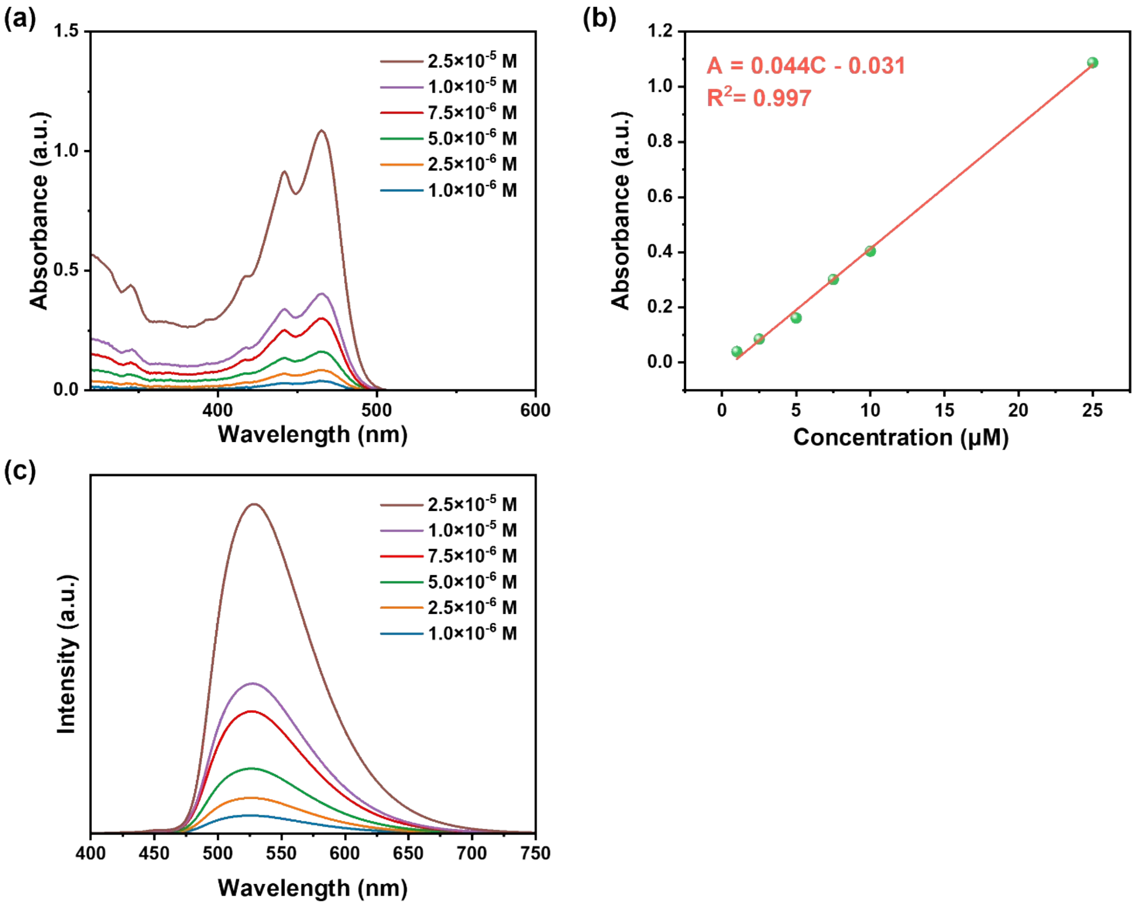

Figure S7. The UV-vis absorption spectra (a) of **BODIQU-tBuCZ** in DCM with different concentrations varied from 1 μM to 0.1 mM. (b) Plots of the absorbance (A) recorded at 460 nm, against the compound concentration (*c*) (ε = 44000 $L{\cdot}mol^{-1}{\cdot}cm^{-1}$). The fluorescence emission spectra (c) of **BODIQU-tBuCZ** in DCM at room temperature with different concentrations. The excitation wavelength was 380 nm.

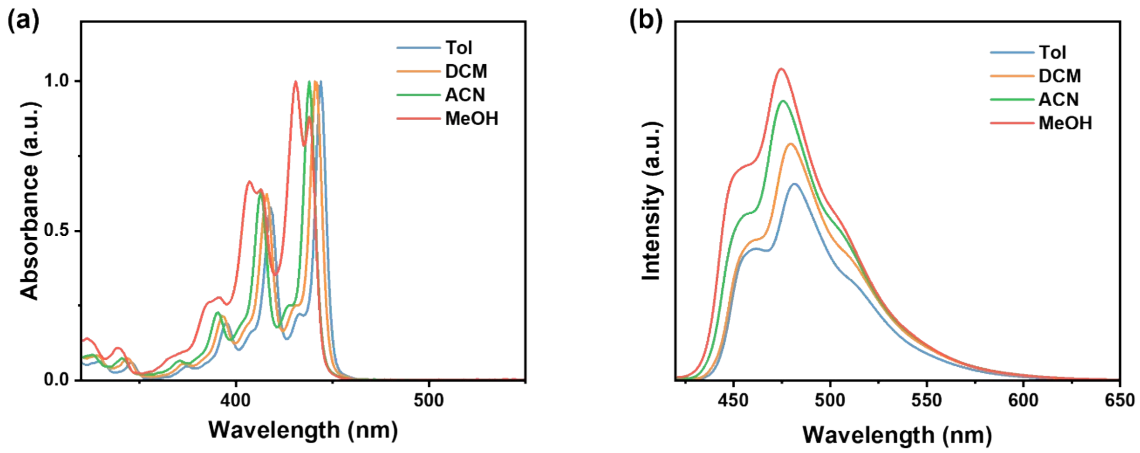

Figure S8. The UV- vis absorption spectra (a), fluorescence emission spectra (b) of **BODIQU-BZI** in different solvents. The concentrations were 5×10^{-5} M.

Figure S8 demonstrates that the electronic transition observed in **BODIQU-BZI** is attributed to a π-π* transition.

Notes**:** Tol: toluene; DCM: dichloromethane; ACN: Acetonitrile; MeOH: methanol.

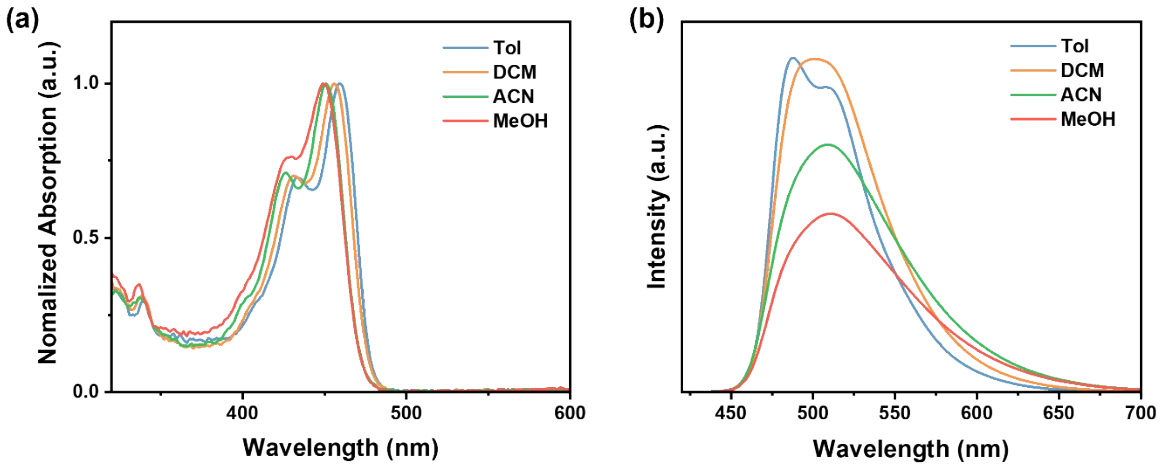

Figure S9. The UV-vis absorption spectra (a), fluorescence emission spectra (b) of **BODIQU-CZ** in different solvents. The concentrations were 5×10^{-5} M.

Figure S9 demonstrates that the electronic transitions in **BODIQU-CZ** involve a combination of π-π* and intramolecular charge transfer (ICT) processes.

Notes**:** Tol: toluene; DCM: dichloromethane; ACN: acetonitrile; MeOH: methanol.

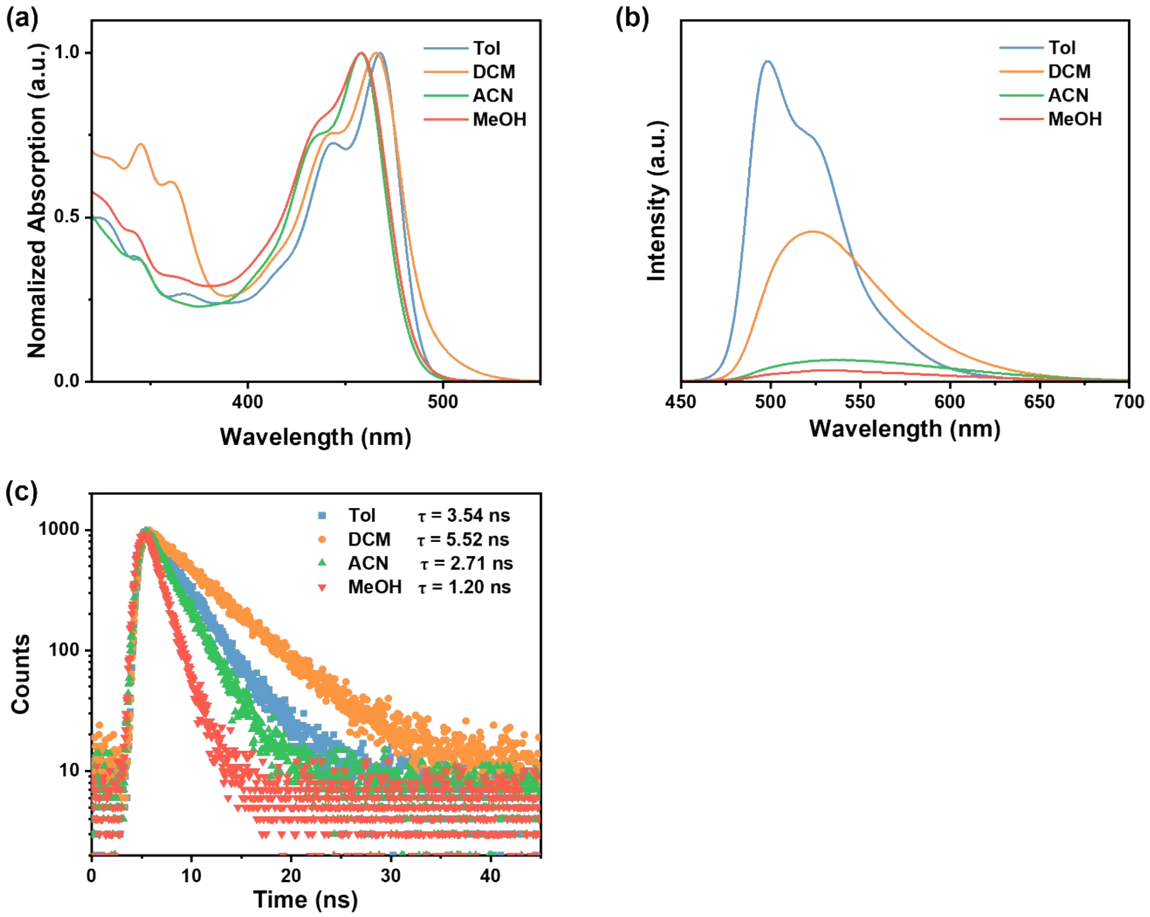

Figure S10. The UV-vis absorption spectra (a), fluorescence emission spectra (b), lifetime (c) of **BODIQU-tBuCZ** in different solvents. The concentrations were 5×10^{-5} M.

Figure S10 demonstrates that the electronic transitions in **BODIQU-tBuCZ** involve a combination of π-π* and intramolecular charge transfer (ICT) processes.

Notes**:** Tol: toluene; DCM: dichloromethane; ACN: acetonitrile; MeOH: methanol.

Table S2. Solvent-Dependent Linear Photophysical Parameters of **BODIQU-tBuCZ**

Solvent	E_T (30)[a]	λ_{abs}/nm	λ_{em}/nm	$\Phi_F{}^b$/%	$\tau_F{}^c$/ns
Tol	33.9	468	498	87.5	3.54
DCM	40.7	465	523	85.9	5.52
MeCN	45.6	458	540	15.5	2.71
MeOH	51.9	458	531	6.2	1.20

Notes**:** [a]Empirical parameters for solvent polarity; [b]fluorescence quantum yield; [c]fluorescence lifetime.

Table S3. Film-state PLQY of Reference Compounds

Compounds	*ΦF*/%
**BODIQU-tBuCZ**	11.1
**BODIQU-CZ**	8.05
**BODIQU-BZI**	5.2

5. Energy Level

All the calculations were carried out using the Gaussian 09 program package. The geometries of **BODIQU-BZI**, **BODIQU-CZ** and **BODIQU-tBuCZ** were fully optimized at the B3LYP/6-311G* level. Harmonic vibrational frequencies were calculated at the same level to check whether the obtained structure is a minimum. The frontier molecular orbitals were calculated for each of the compounds. In this work, all the calculations were performed in vacuum without considering the solvent.

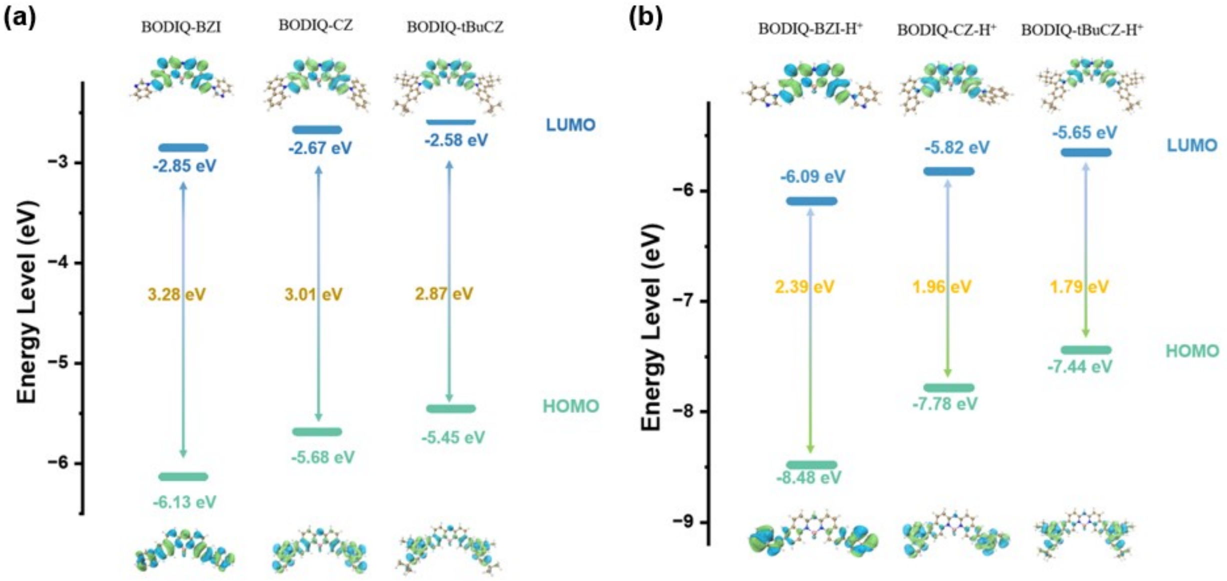

Figure S11. The HOMO and LUMO of **BODIQU** and **BODIQU-H$^+$**.

6. Response for BODIQU Derivatives to DCP Detection

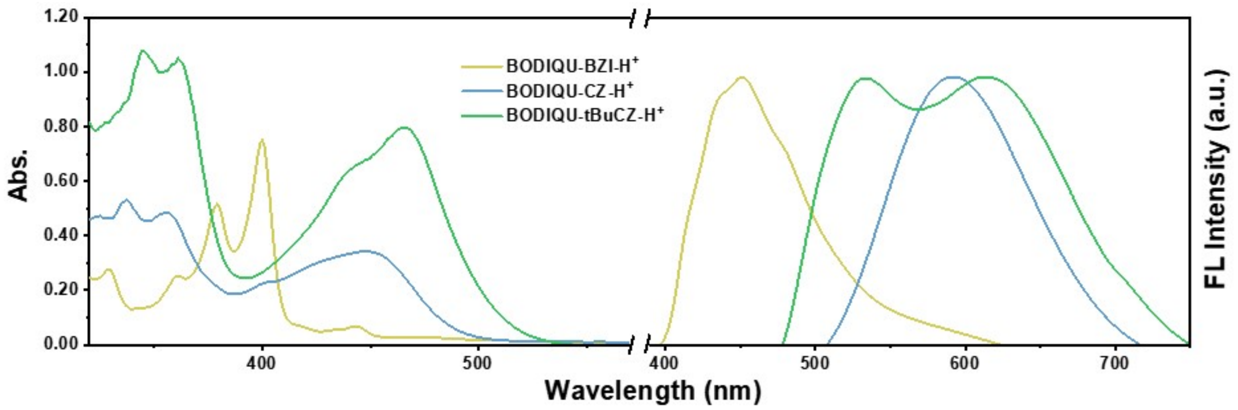

Figure S12. The UV-vis absorption and fluorescence emission spectra of **BODIQU-BZI**, **BODIQU-CZ**, **BODIQU-tBuCZ** to DCP detection in DCM.

Figure S12 demonstrates significant spectral changes in **BODIQU-BZI**, **BODIQU-CZ**, and **BODIQU-tBuCZ** upon protonation.

Table S4. Key Linear Photophysical Parameters of Reference Compounds

Compounds	λ_{abs}/nm	λ_{em}/nm	$\Phi_F{}^a$/%	$\tau_F{}^b$/ns
**BODIQU- BuCZ**	468	523	87.5	5.74
**BODIQU-CZ**	456	510	88.1	3.81
**BODIQU-BZI**	441	480	13.5	1.59
**BODIQU-tBuCZ-H$^+$**	345	613	22.6	4.23
**BODIQU-CZ-H$^+$**	338	592	31.8	10.61
**BODIQU-BZI-H$^+$**	400	451	3.0	2.05

Notes: [a]fluorescence quantum yield; [b]fluorescence lifetime.

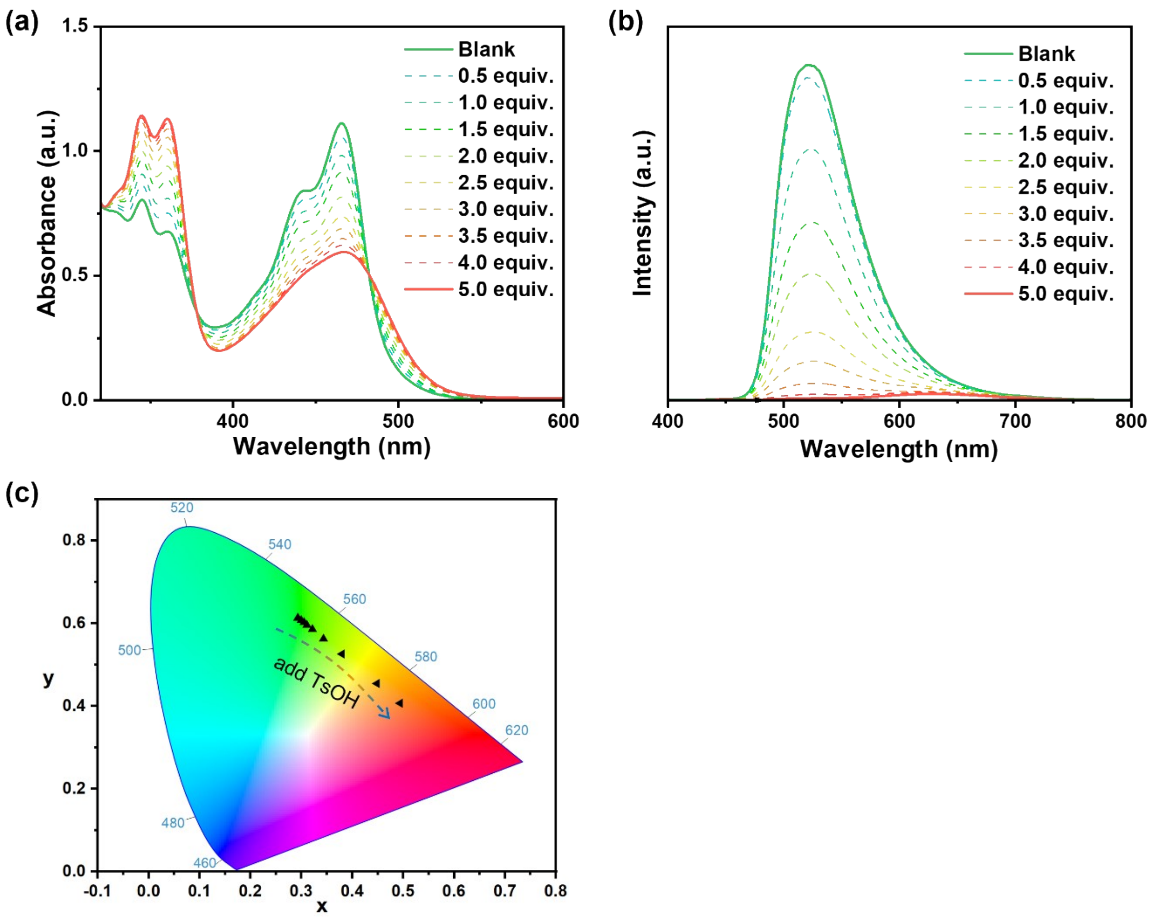

Figure S13. UV-vis absorption spectra (a), fluorescence emission spectra (b) and CIE (c) of **BODIQU-tBuCZ** titrated by p-toluenesulfonic acid in DCM.

Figure S13 demonstrates pronounced protonation characteristics in the UV-vis absorption spectrum, fluorescence emission spectrum of **BODIQU-tBuCZ** upon TsOH titration.

Notes: TsOH: p-toluenesulfonic acid.

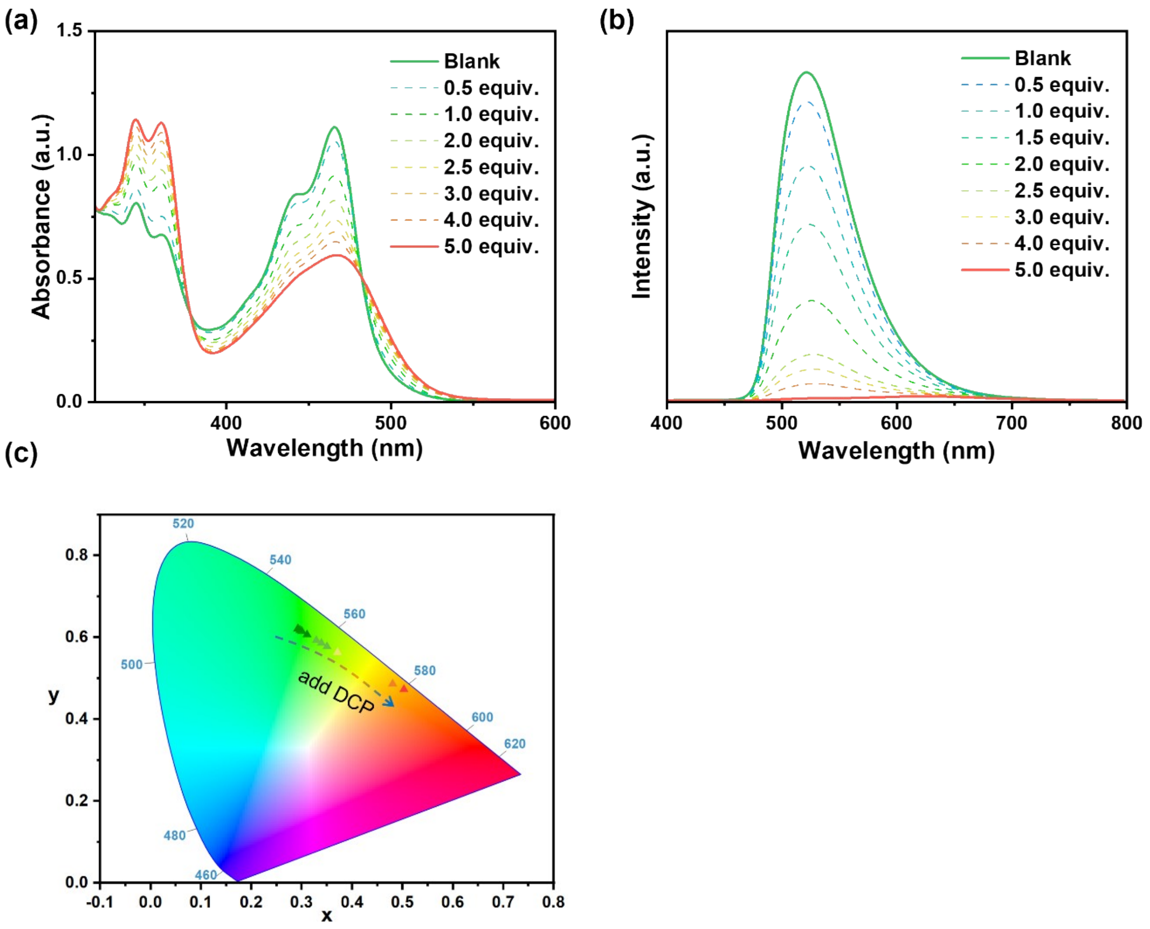

Figure S14. UV-vis absorption spectra (a), fluorescence emission spectra (b) and CIE (c) of **BODIQU-tBuCZ** titrated by DCP in DCM.

Figure S14 demonstrates that DCP titration induces UV-vis absorption spectrum and fluorescence emission spectrum changes in **BODIQU-tBuCZ** analogous to those observed in **Figure S13**, indicating a protonation reaction with DCP.

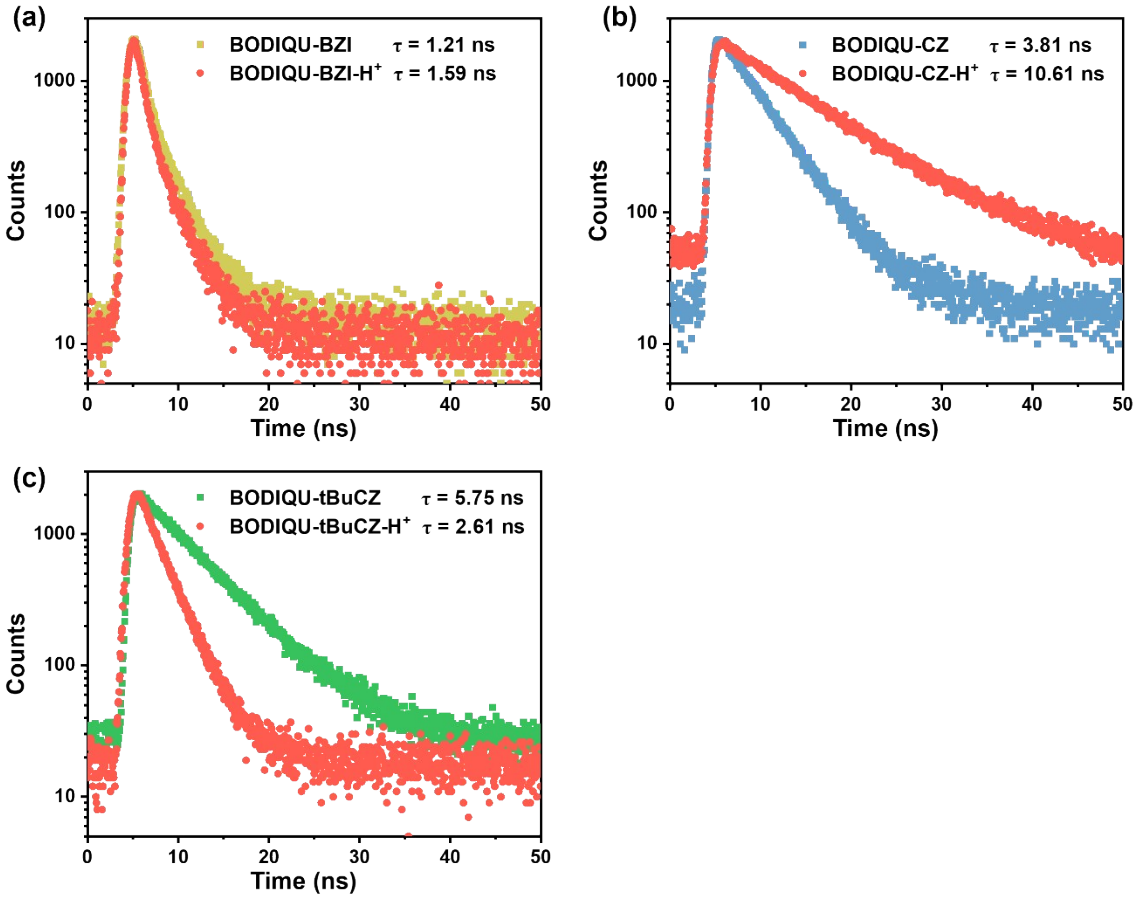

Figure S15. Fluorescence lifetime of **BODIQU-BZI** (a), **BODIQU-CZ** (b), **BODIQU-tBuCZ** (c) to DCP detection in DCM.

Figure S15 demonstrates significant changes in the fluorescence lifetimes of **BODIQU-BZI**, **BODIQU-CZ**, and **BODIQU-tBuCZ** before and after protonation.

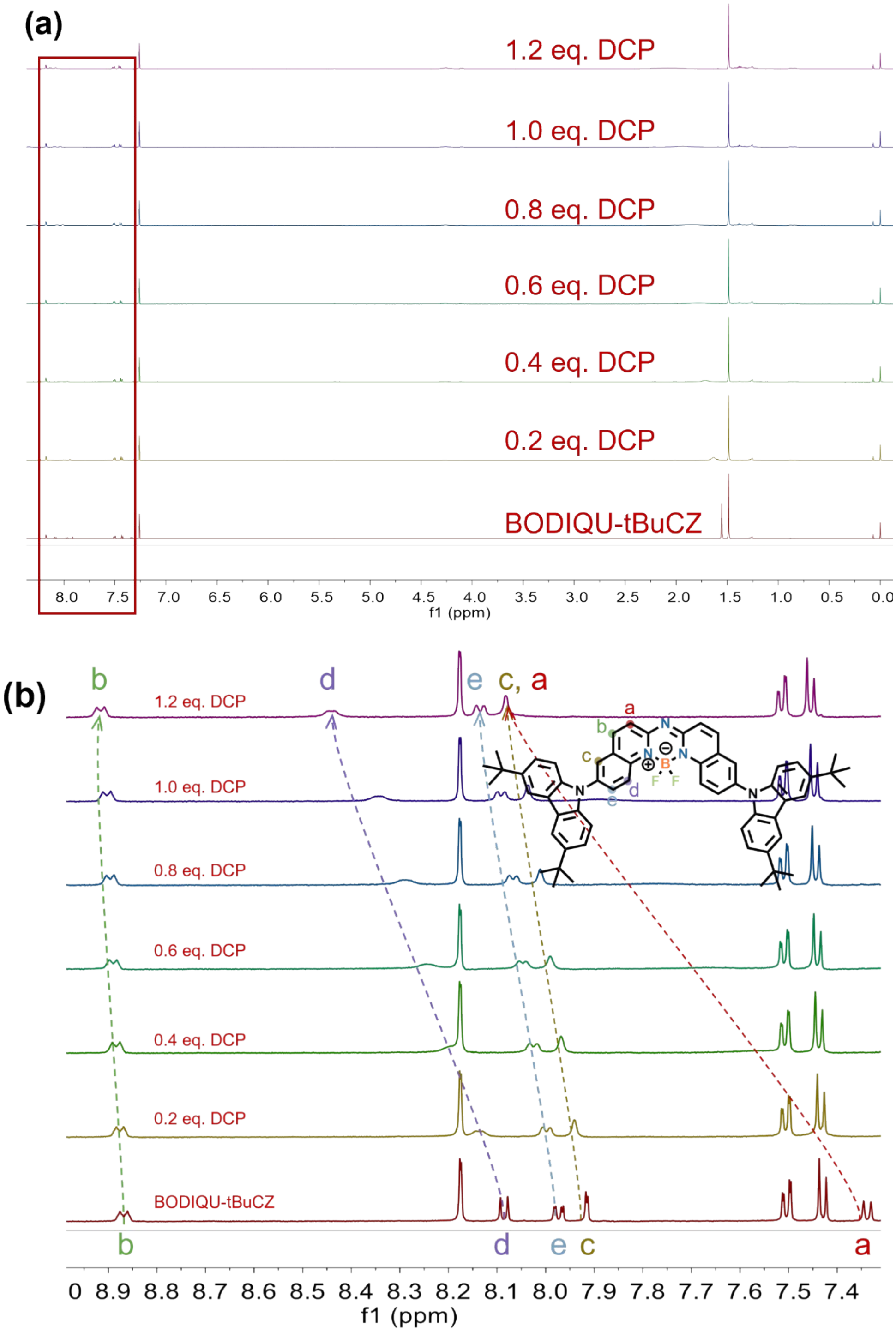

Figure S16. (a) ^{1}H NMR titration experiments with **BODIQU-tBuCZ** titrated with DCP. (b) Zoom-in of the selected region.

Figure S16 showed that the displacement of the hydrogen atoms on the benzene ring of the core unit BODIQU was significantly shifted in the low-field direction with increasing DCP titration equivalents, demonstrating that **BODIQU-tBuCZ** interacted significantly with DCP.

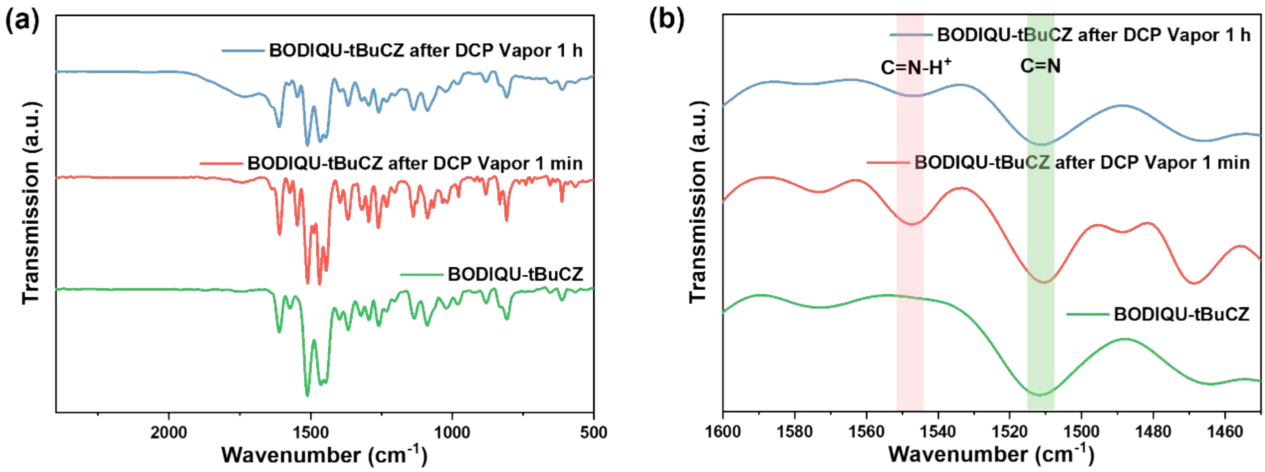

Figure S17. (a) FT-IR spectra of the **BODIQU-tBuCZ** before and after DCP treatment. (b) Partial enlarged view of the spectra.

Figure S17 demonstrates the emergence of the C=N-H$^+$ bond in **BODIQU-tBuCZ** thin films upon DCP vapor exposure, confirming protonation at the central nitrogen atom of the **BODIQU** core.

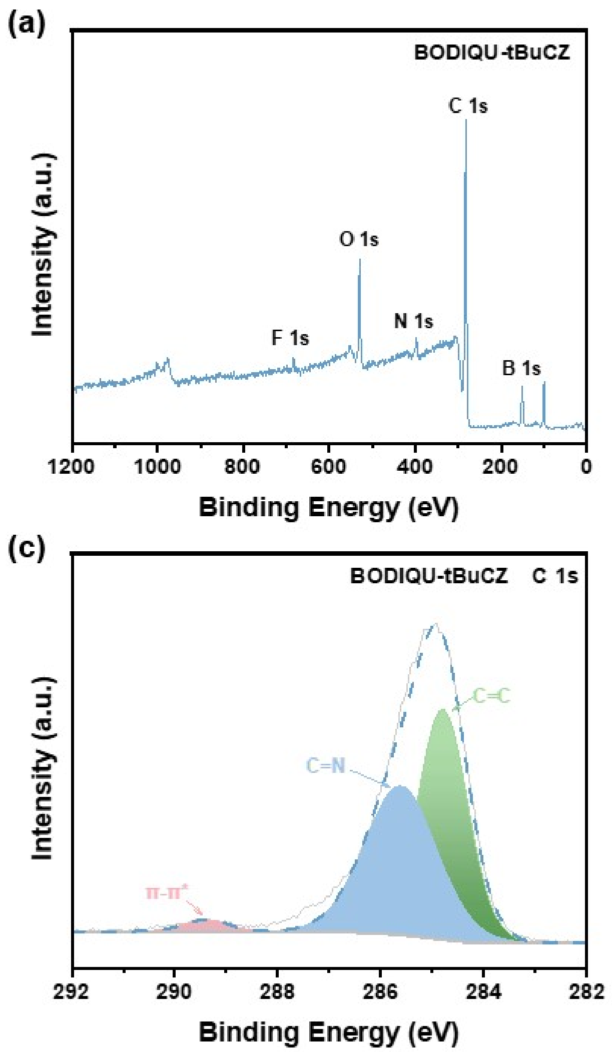

Figure S18. XPS full spectrum (a); high-resolution C 1s (b) and N 1s XPS spectra (c) of **BODIQU-tBuCZ**.

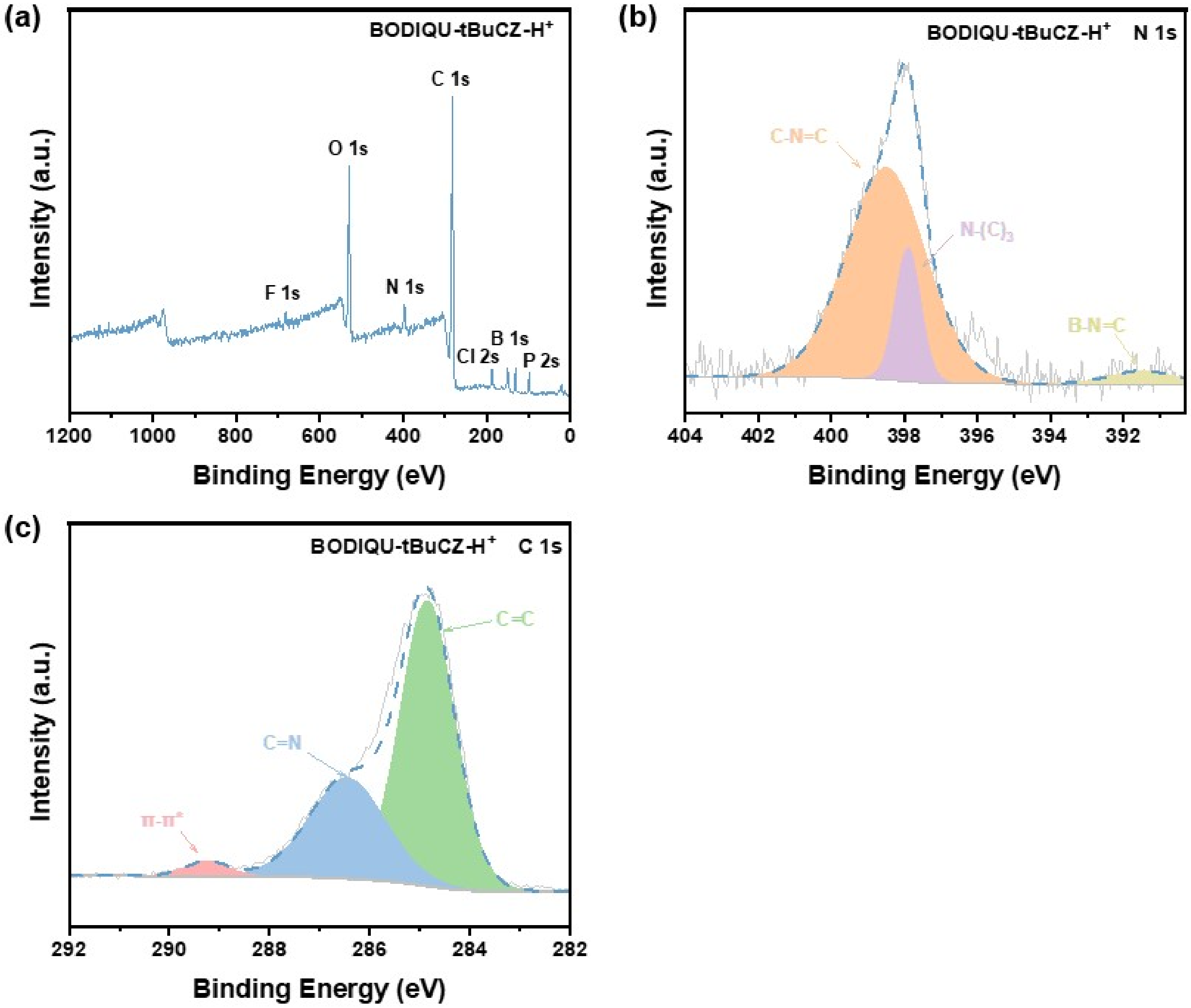

Figure S19. XPS full spectrum (a); high-resolution C 1s (b) and N 1s XPS spectra (c) of **BODIQU-tBuCZ-H**$^+$.

Figure S19 demonstrates the emergence of Cl 2s and P 2s peaks alongside a C=N bond shift in **BODIQU-tBuCZ** upon DCP-induced protonation.

7. Limit of Detection (LOD) Calculation

The detection limit was calculated using the standard 3σ/S method recommended by IUPAC:

$$LOD = \frac{3\sigma}{S}$$

where σ represents the standard deviation of baseline noise, and S is the slope of the calibration curve. Experimentally, we determined σ (1.11) by performing at least 10 independent measurements of the sensor's stable fluorescence baseline under analyte-free conditions (in air or carrier gas). The calibration curve was established by measuring fluorescence intensity changes at various DCP concentrations (0.01 ppt, 0.05 ppt, 0.1 ppt, etc.) near the expected detection limit, with the slope S (-1.181) derived from linear regression analysis.

8. Sensing Performance of BODIQU Film

The fluorescent sensor consists of a photodiode (peak wavelength: 365 nm; FWHM: 30 nm; optical density: 6), an integrated tubular flow cell containing a fluorescent sensing window, a solid-state photodetector (peak wavelength: 490 nm; FWHM: 40 nm; optical density: 6), and a readout unit. The fluorescent sensing window is created by removing the opaque polyimide coating from the outer surface of the capillary. The remainder of the capillary column facilitates analyte mixture separation. As each separated analyte passes through the sensing window, it induces a measurable change in the fluorescence signal, thereby enabling simultaneous separation and detection.

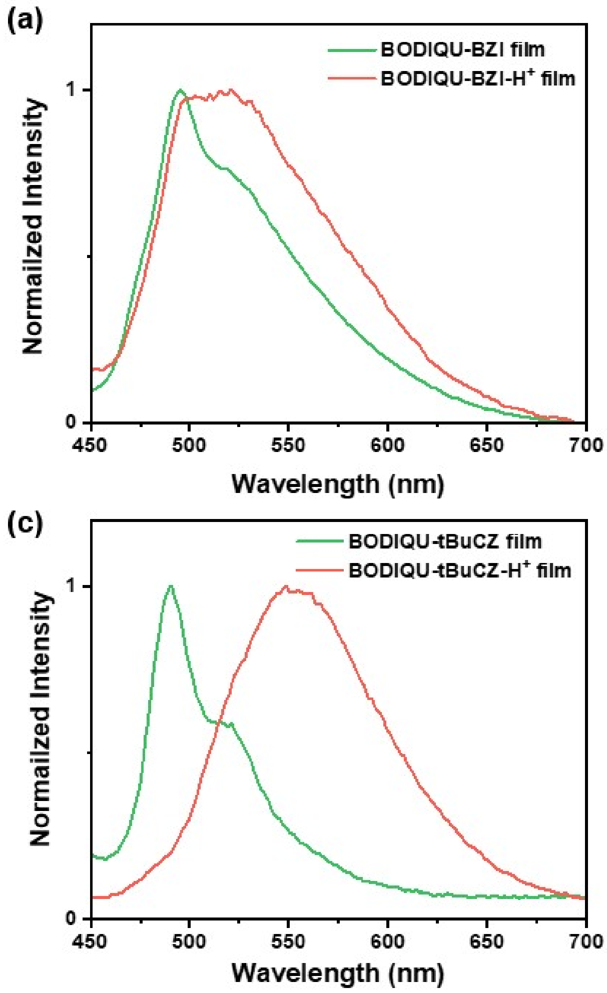

Figure S20. Emission spectra of **BODIQU-BZI** film (a), **BODIQU-CZ** film (b) and **BODIQU-tBuCZ** film (c) before and after DCP vapor exposure.

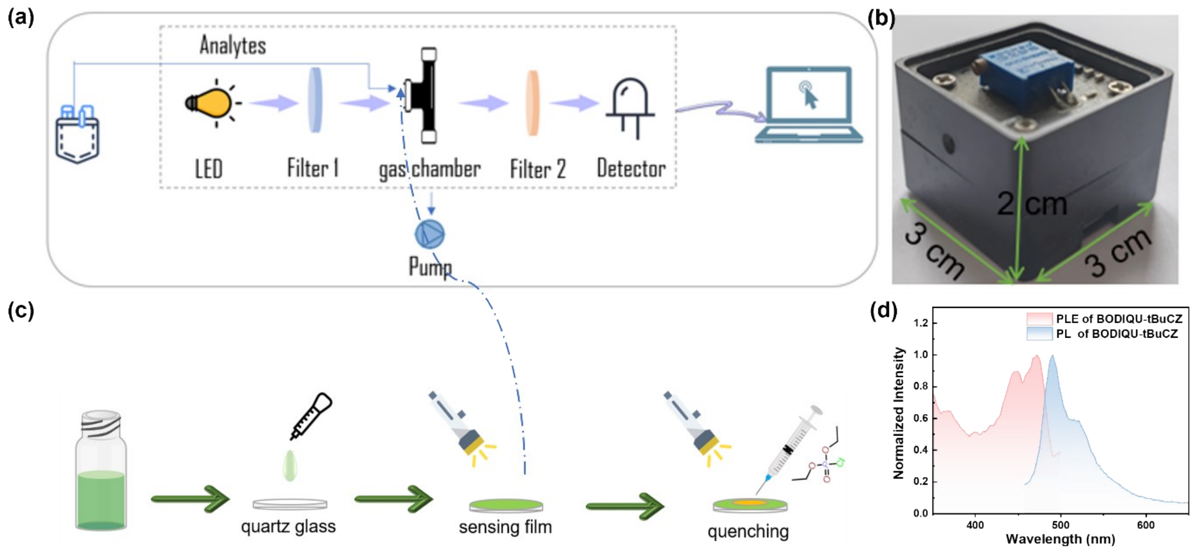

Figure S21. (a) Schematic representation of the homemade conceptual sensing platform. (b) Image of the assembled sensor prototype. (c) Process for the preparation of **BODIQU-tBuCZ** fluorescent sensing films. (d) Excitation and emission spectra of **BODIQU-tBuCZ**.

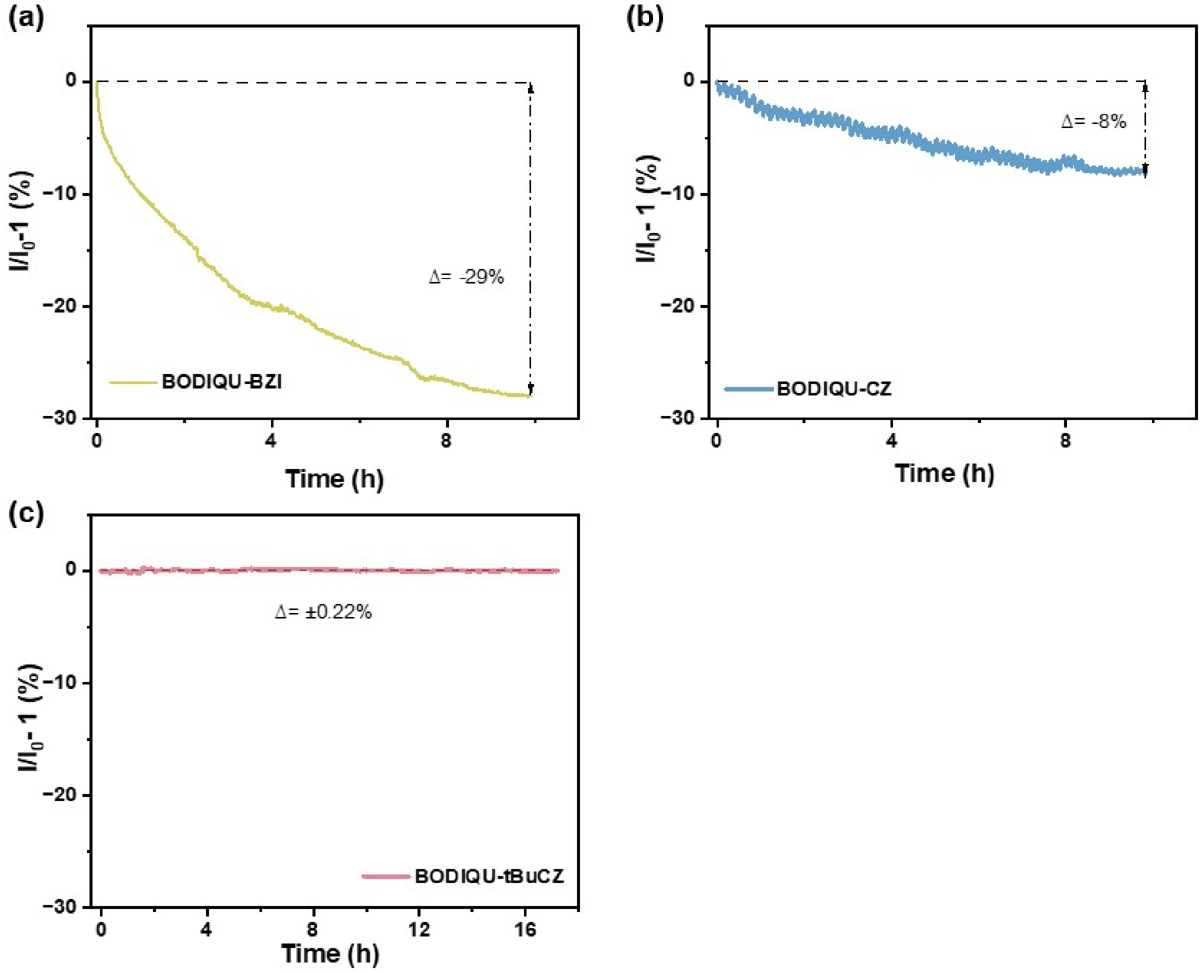

Figure S22. Photo-chemical stability of **BODIQU-BZI** (a), **BODIQU-CZ** (b), **BODIQU-tBuCZ** (c) in the film state monitored at 490 nm with 365 nm as the excitation wavelength.

Figure S22 demonstrates a significant enhancement in the stability of **BODIQU-BZI** film, **BODIQU-CZ** film, and **BODIQU-tBuCZ** film.

Notes: I_0 and I represent the fluorescence intensity of the sensing film with absence and presence of analytes, respectively.

Table S5. Comparison of the present work with the reported methods on the detection of DCP

Methods	DCP DL	Response time	Linear range	Reversibility	Portable device	Robust durability	References
**Fluorescence**	**0.001 ppt**	**< 3 s**	**0.001 ppt-1 ppm**	**Yes**	**Yes**	**50**	**This Work**
Fluorescence	0.01 ppt	< 2 s	0.01 ppt-100 ppm	No	Yes	3	Ref. 2
Fluorescence	0.1 ppt	< 2 s	0.1 ppt-1 ppm	Yes	Yes	30	Ref. 3
Fluorescence	26 ppb	< 5 s	26 ppb-830 ppb	Yes	Yes	100	Ref. 4
Fluorescence	2.5 ppt	< 5 s	2.5 ppb-10ppm	No	Yes	7	Ref. 5
Electrochemistry	28 ppb	< 1 min	28 ppb-2.3 ppm	No	Yes	/	Ref. 6
Colorimetry / Fluorescence	0.8 ppb	< 5 s	0.005 ppm-0.03 ppm	Yes	No	/	Ref. 7
Colorimetry / Fluorescence	0.43 ppb	< 5 s	0.01 ppm-0.03 ppm	Yes	Yes	/	Ref. 8

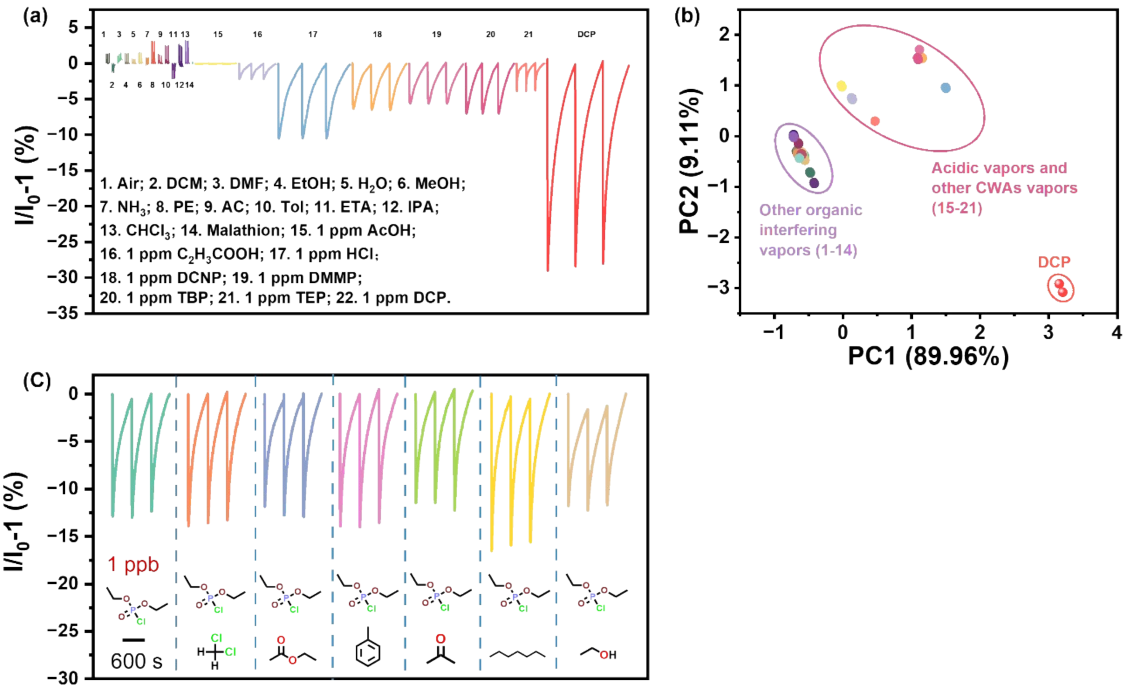

Figure S23. (a) Response intensity of the sensor to the presence of DCP and other vapors. (b) Two-dimensional PCA score plot based on fluorescence response intensity, recovery time, and response time for discrimination of chemical analytes (n = 6 replicates per analyte). (c) Interaction reaction of **BODIQU-tBuCZ** based films. Response of films to mixtures of DCP vapours (1 ppb) and other organic interfering vapours.

Figure S23 demonstrates that **BODIQU-tBuCZ** film exhibit remarkable selectivity for DCP over common interfering gases, while maintaining detectable responses to low-concentration DCP even in saturated interfering gas environments.

Notes: DCM: dichloromethane; EtOH: ethanol; MeOH: methanol; NH_3: ammonia; PE: petroleum ether; AC: acetone; Tol: toluene; ETA: ethanolamine; IPA: isopropyl alcohol; AcOH: acetic acid; HCl: hydrochloric acid; DCNP: diethyl cyanophosphonate; DMMP: dimethyl methylphosphonate;

TBP: Tri-n-butyl phosphate; TEP: triethyl phosphate.

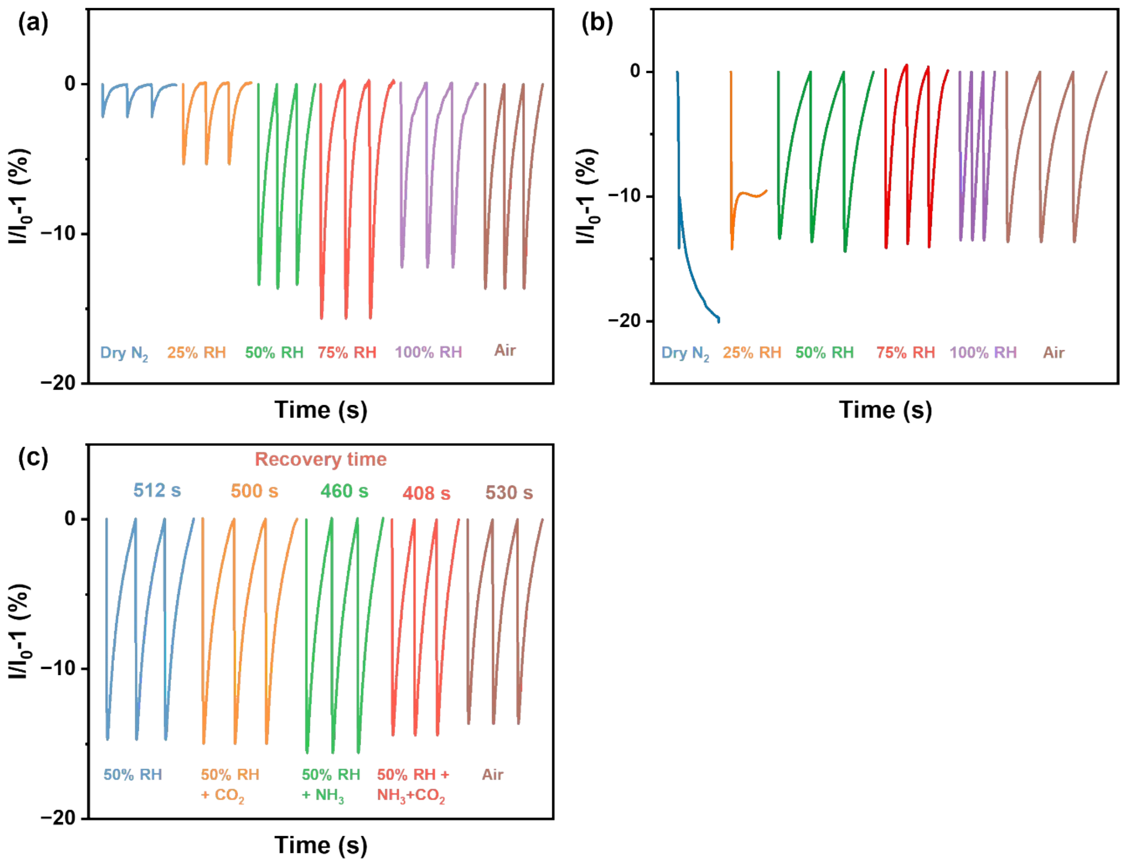

Finger S24. (a) Response of **BODIQU-tBuCZ** fluorescent sensing films to diluted DCP at different humidity levels. (b) Fluorescence recovery behavior of **BODIQU-tBuCZ** films under purging at varying humidity levels. (c) Fluorescence response of the **BODIQU-tBuCZ** film upon purging with trace basic substances (1 % CO_2 or NH_3).

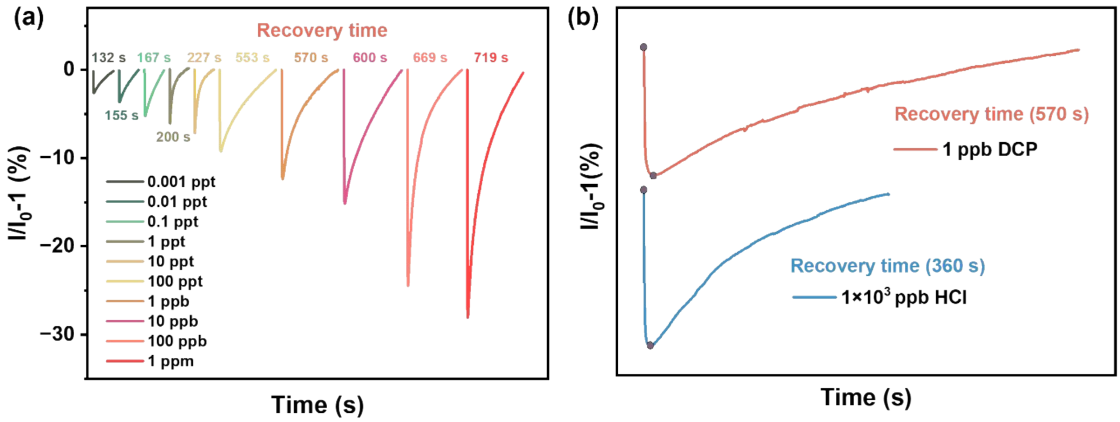

Figure S25. (a) Recovery time of **BODIQU-tBuCZ** films following exposure to different DCP concentrations. (b) Sensing kinetic curve of **BODIQU-tBuCZ** response to HCl, DCP. The recovery time is defined as the duration required for the system to regain 80% of its initial state.

Figure S25 demonstrates the discrimination capability of **BODIQU-tBuCZ** film between DCP and HCl through response kinetic analysis.

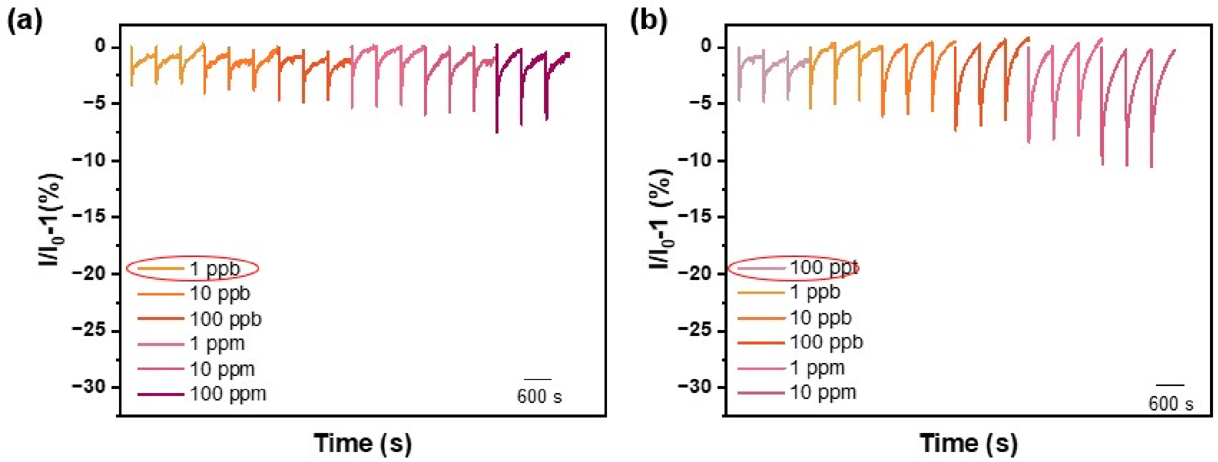

Figure S26. Response intensity of the **BODIQU-BZI** film (a), **BODIQU-CZ** film (b) to DCP vapor of different concentrations.

9. Morphology Data

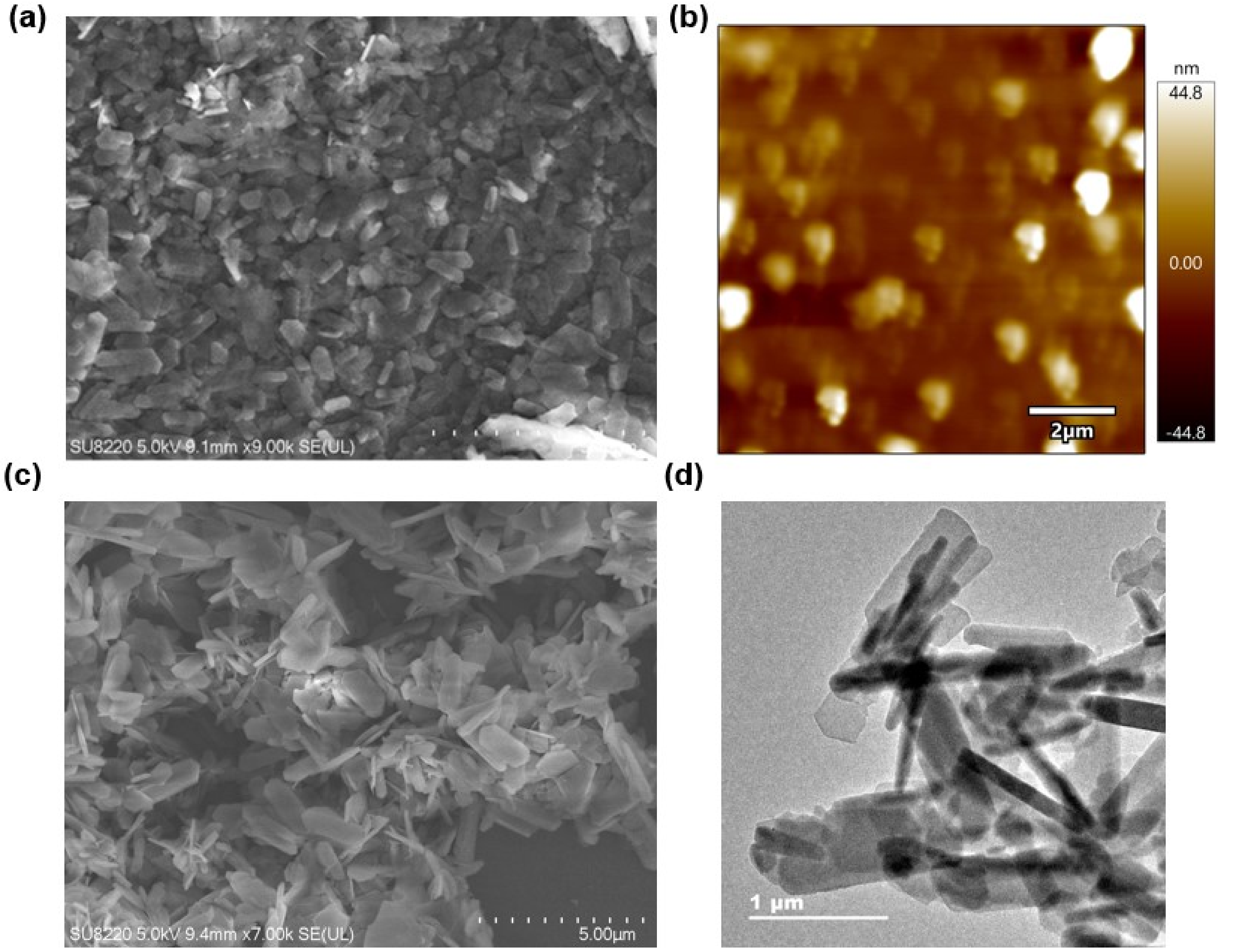

Figure S27. SEM (a), AFM (b), TEM (d) of the **BODIQU-BZI** film, SEM (c) of the **BODIQU-BZI-H**$^{+}$ film.

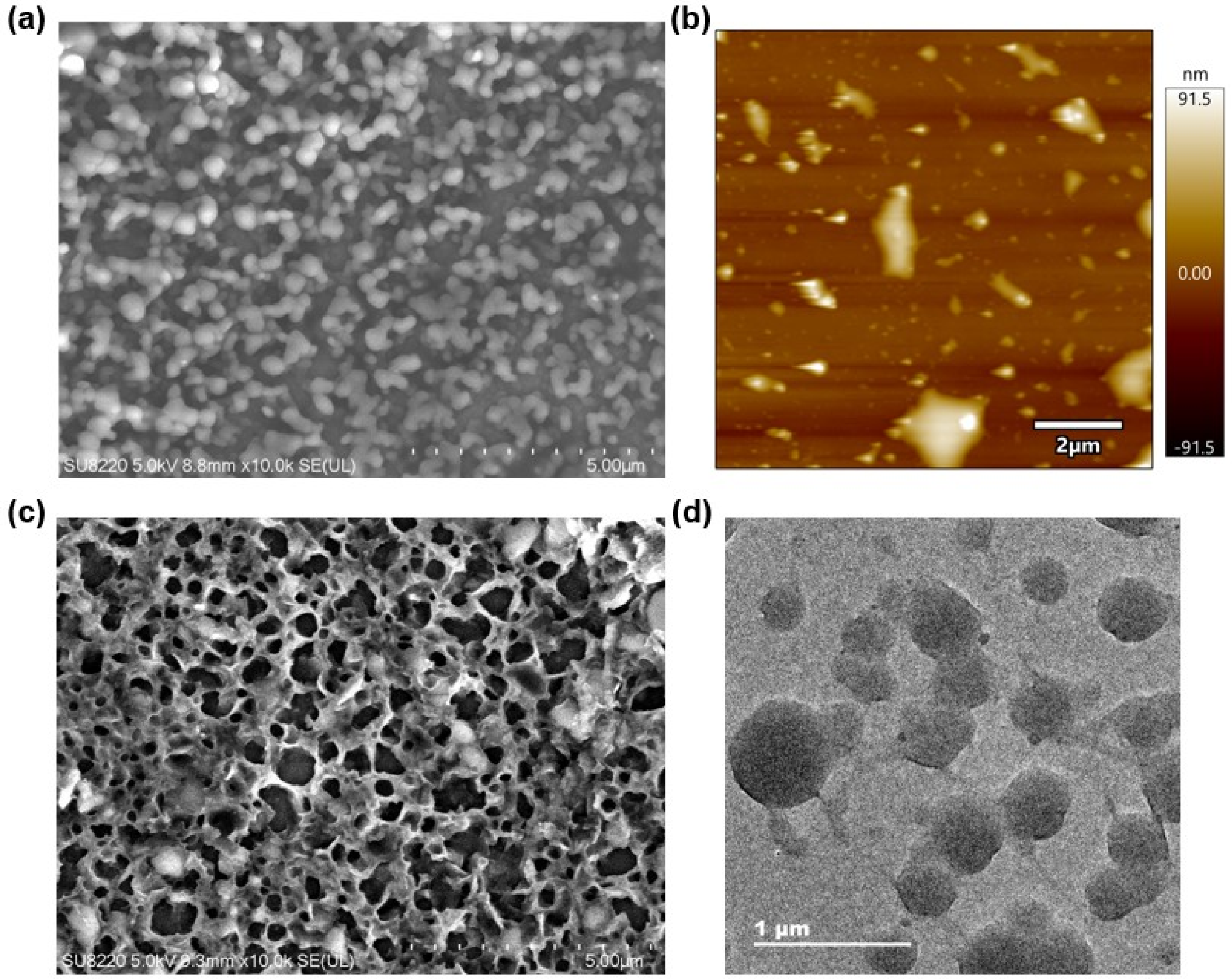

Figure S28. SEM (a), AFM (b), TEM (d) of the **BODIQU-CZ** film, SEM (c) of the **BODIQU-CZ⁺** film.

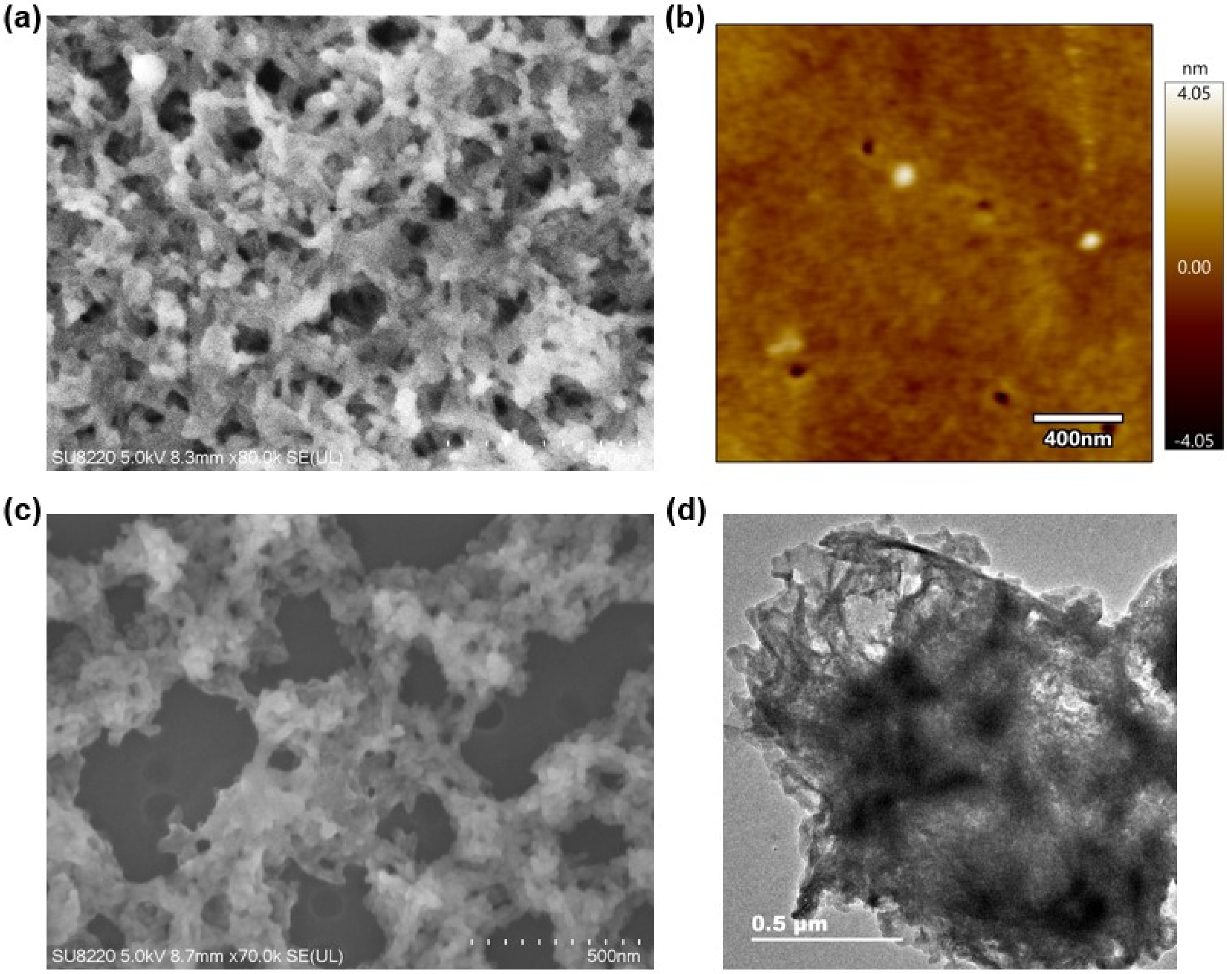

Figure S29. SEM (a), AFM (b), TEM (d) of the **BODIQU-tBuCZ** SEM (c) of the **BODIQU-tBuCZ-H$^+$** film.

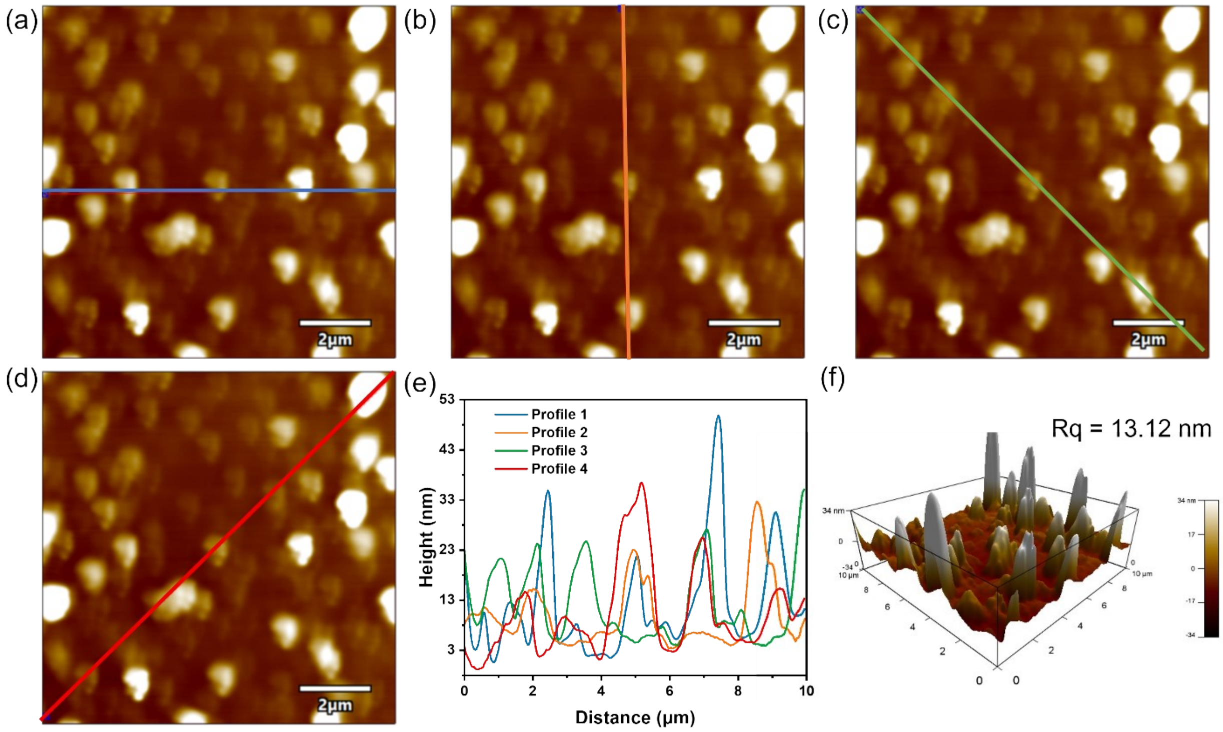

Finger S30. (a-d) AFM images (10 μm × 10 μm) of **BODIQU-BZI** films, (e) Height profile extracted from the topography scan, showing a maximum peak-to-valley difference of 51 nm. (f) 3D rendered view of the same region. The average surface roughness (Rq) is 13.12 nm.

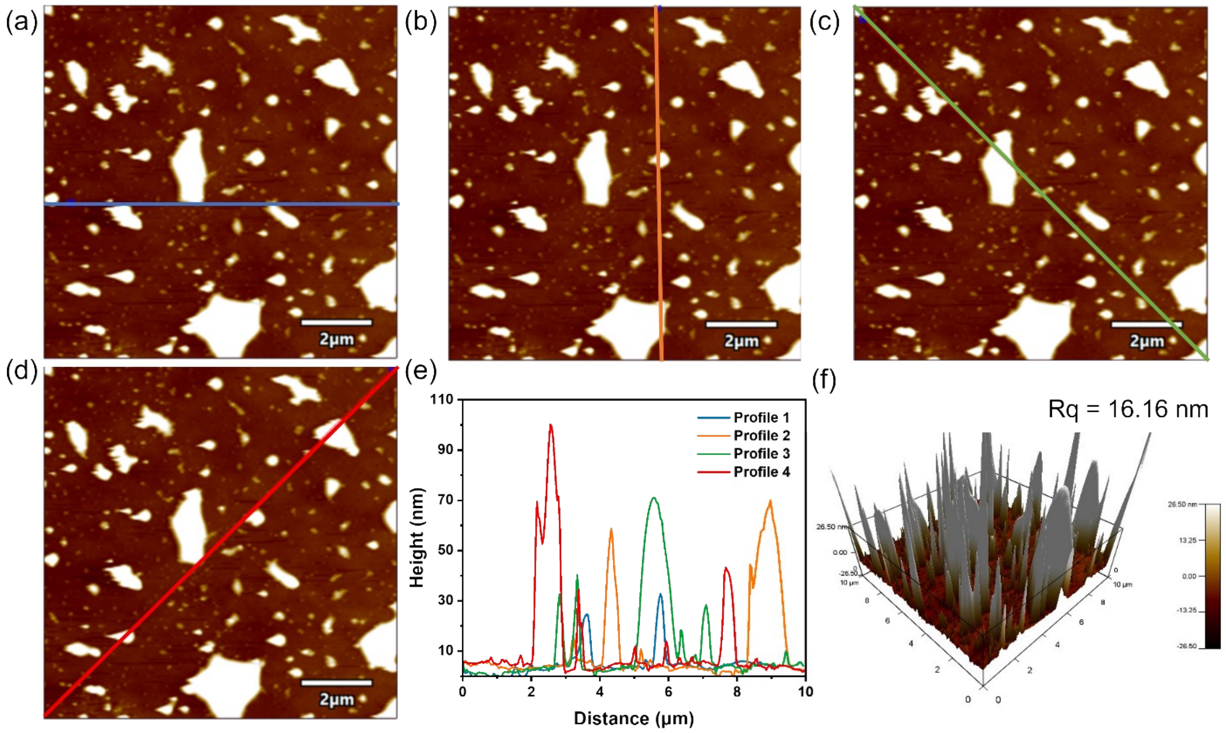

Finger S31. (a-d) AFM images (10 μm × 10 μm) of **BODIQU-CZ** films, (e) Height profile extracted from the topography scan, showing a maximum peak-to-valley difference of 100 nm. (f) 3D rendered view of the same region. The average surface roughness (Rq) is 16.16 nm.

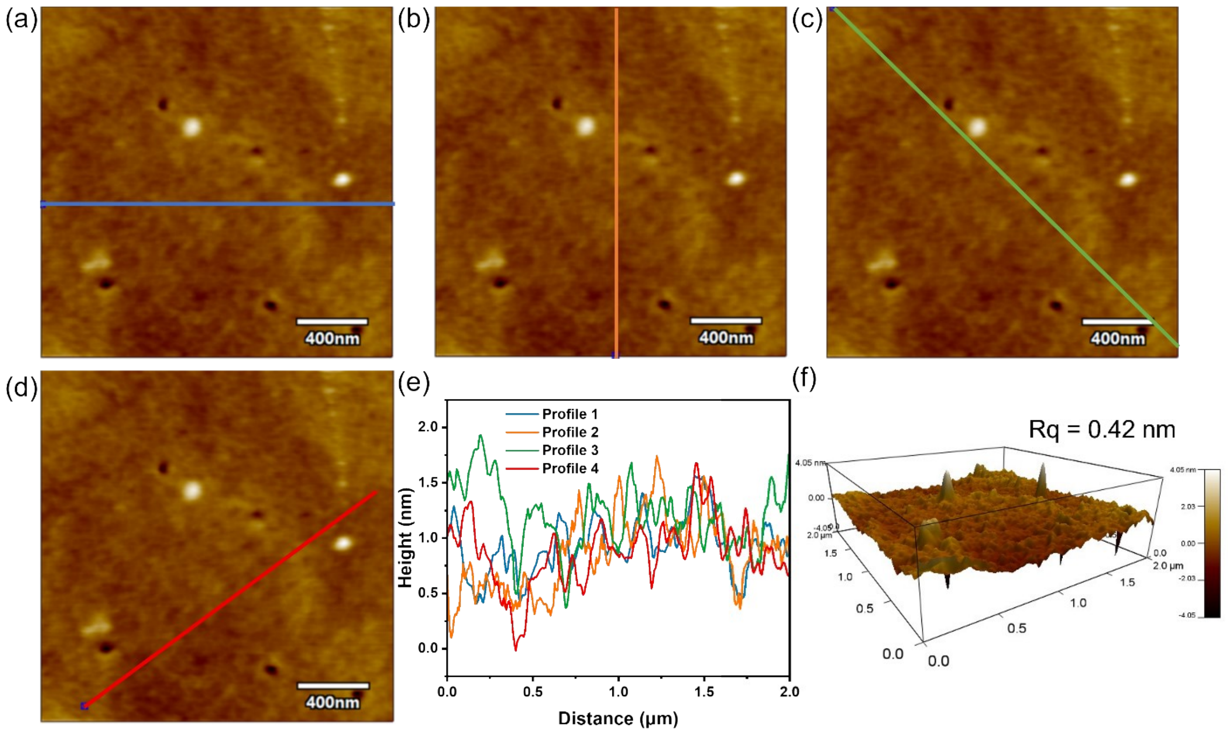

Finger S32. (a-d) AFM images (2 μm × 2 μm) of **BODIQU-tBuCZ** films, (e) Height profile extracted from the topography scan, showing a maximum peak-to-valley difference of 1.9 nm. (f) 3D rendered view of the same region. The average surface roughness (Rq) is 0.42 nm.

10.TA Data

Transient Absorption (TA) measurements were conducted using a pump-probe spectrometer setup. Initially, a Ti:sapphire amplifier generated a fundamental laser pulse with a wavelength of 800 nm, operating at a repetition rate of 1 kHz. This fundamental pulse was then split into two branches by a beam splitter. One branch was directed towards an optical parametric amplifier to generate the pump pulse at 380 nm. The pump pulse, modulated at a frequency of 500 Hz, underwent attenuation via neutral-density filter wheels. Simultaneously, the other branch of the fundamental pulse was focused into a sapphire crystal to produce a white-light continuum spanning from 350 nm to 1600 nm, utilized as the probe. Time delays between the pump and probe pulses were achieved using a motorized translation stage with a retro-reflecting mirror. The pump and probe beams were spatially overlapped on the sample surface, both being incident on the sample normally. The focused spot size at the sample position was approximately 200 μm for the probe beam and 600 μm for the pump beam.

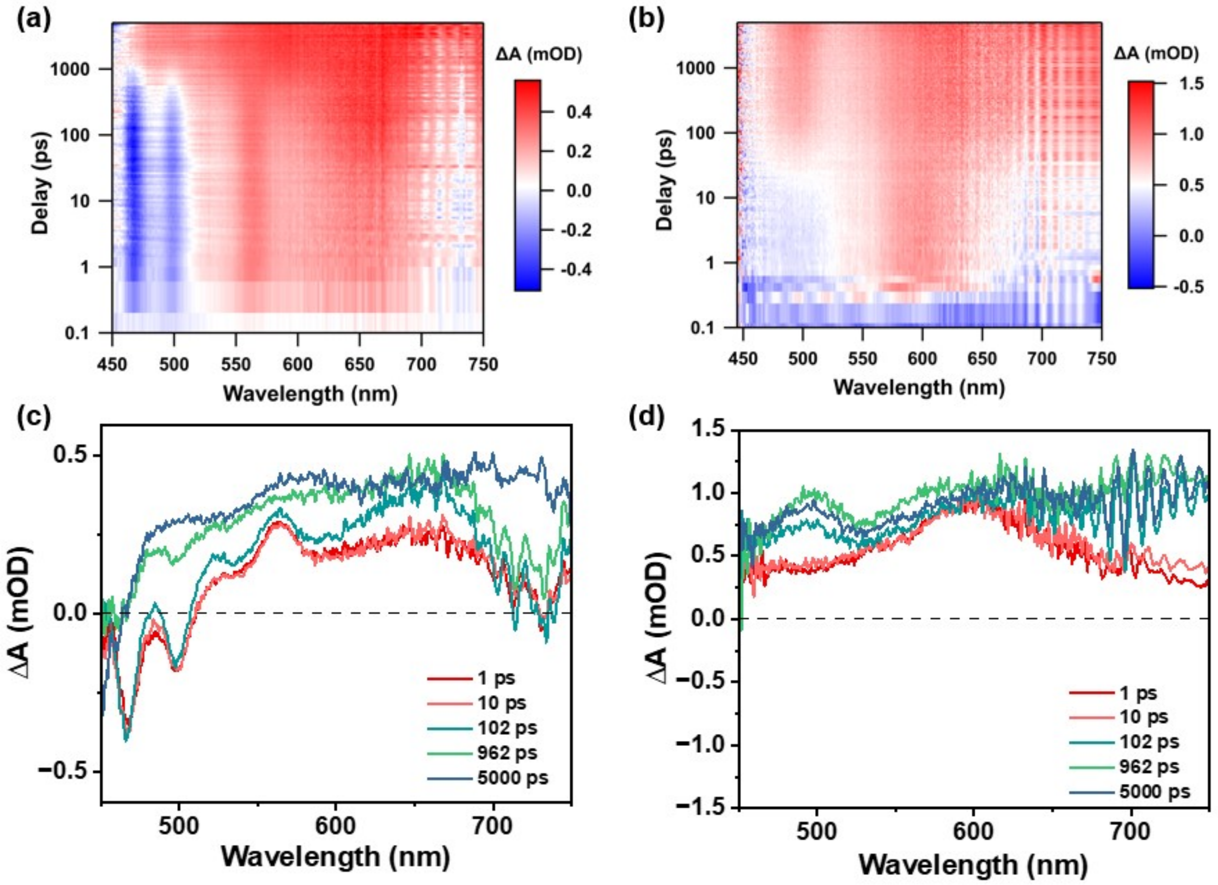

Figure S33. Time-resolved spectra of **BODIQU-BZI** before and after protonation (a) Pseudo color spectra of transient absorption (TA) of **BODIQU-BZI** and (b) **BODIQU-BZI-H$^+$**. (c) TA spectra of **BODIQU-BZI** and (d) **BODIQU-BZI-H$^+$** with different time delays.

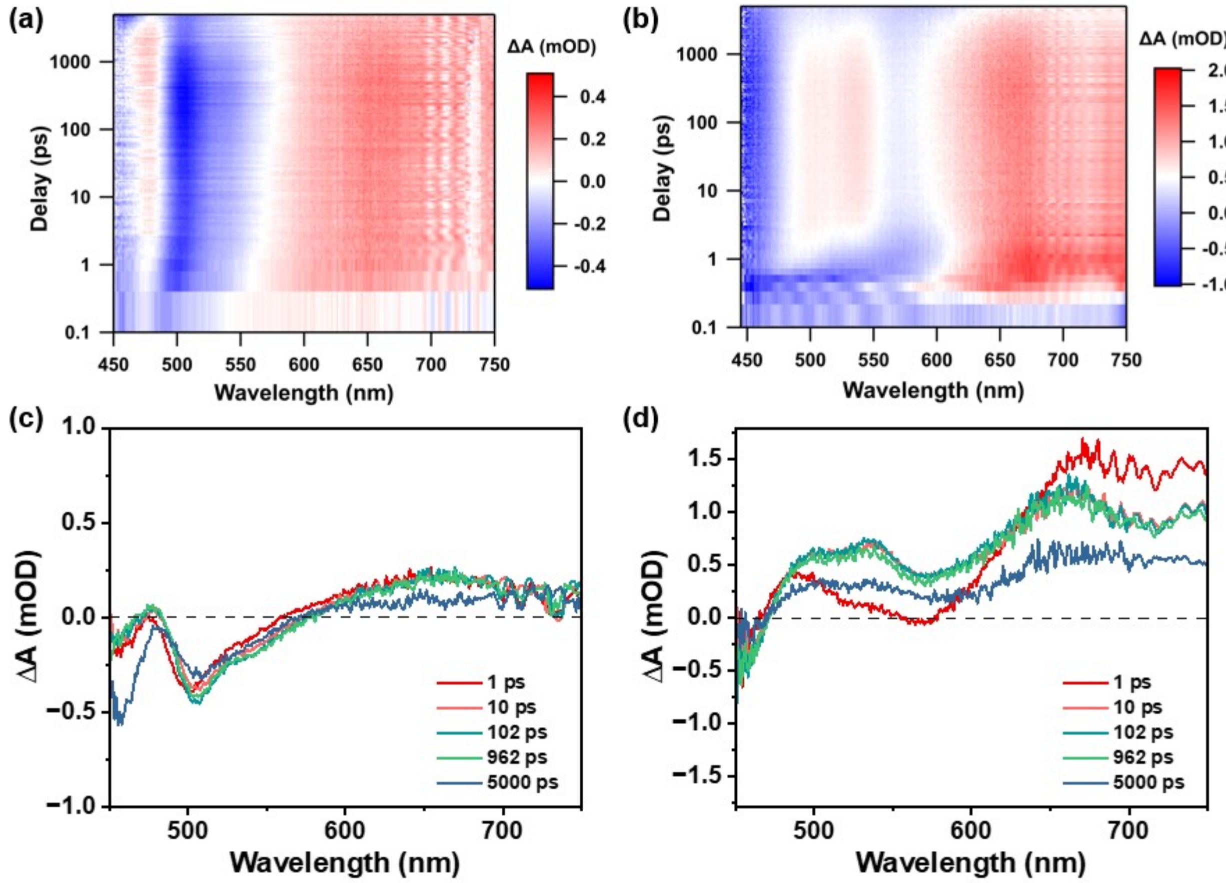

Figure S34. Time-resolved spectra of **BODIQU-CZ** before and after protonation (a) Pseudo color spectra of transient absorption (TA) of **BODIQU-CZ** and (b) **BODIQU-CZ-H**$^+$. (c) TA spectra of **BODIQU-CZ** and (d) **BODIQU-CZ-H**$^+$ with different time delays.

11.Temperature-Dependent Current-Voltage (I-V) Characterization

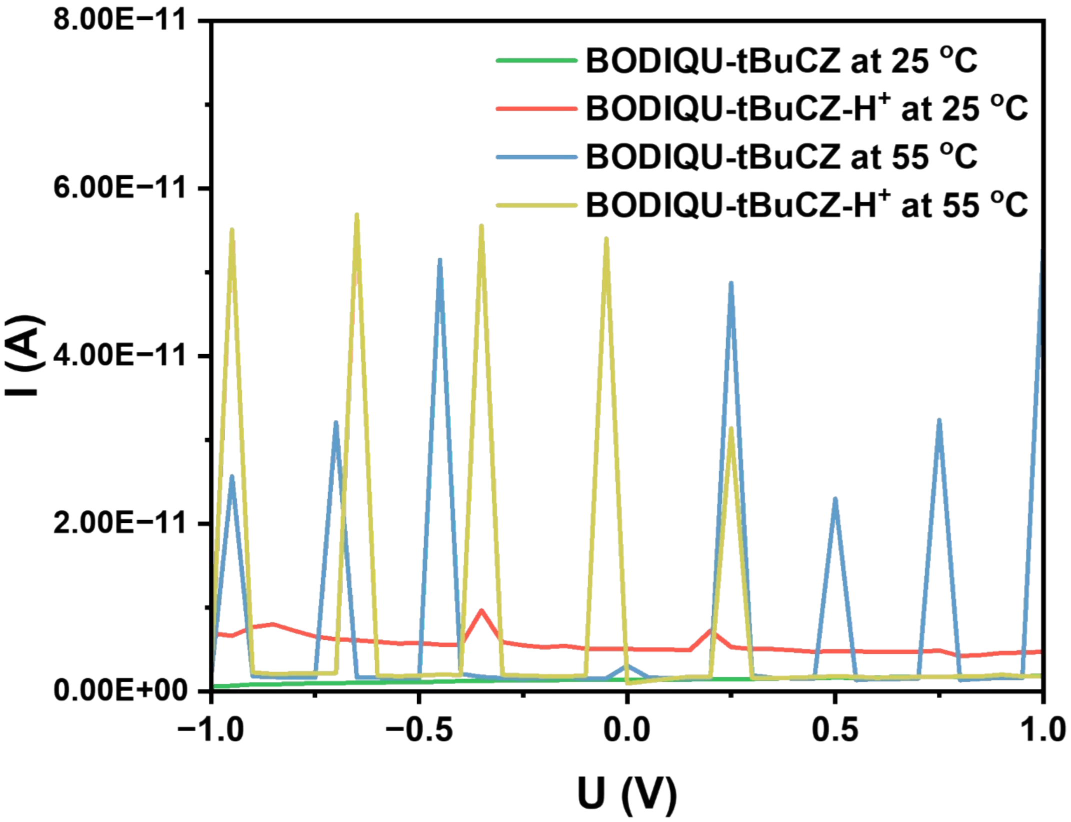

Finger S35. Temperature-dependent I-V tests of **BODIQU-tBuCZ** films before and after protonation.

Figure S35 demonstrates the materials exhibited extremely weak current responses regardless of DCP presence, demonstrating near-insulating behavior at 25 °C. However, upon heating to 55 °C, both systems showed pronounced thermally activated current peaks, suggesting a strongly temperature-dependent charge transport process potentially mediated by resonant tunneling mechanisms. Notably, at 55 °C, the current peak intensity significantly increased upon DCP addition, clearly indicating that the analyte facilitates charge transport at elevated temperatures.

12.NMR and MS Characterization Data

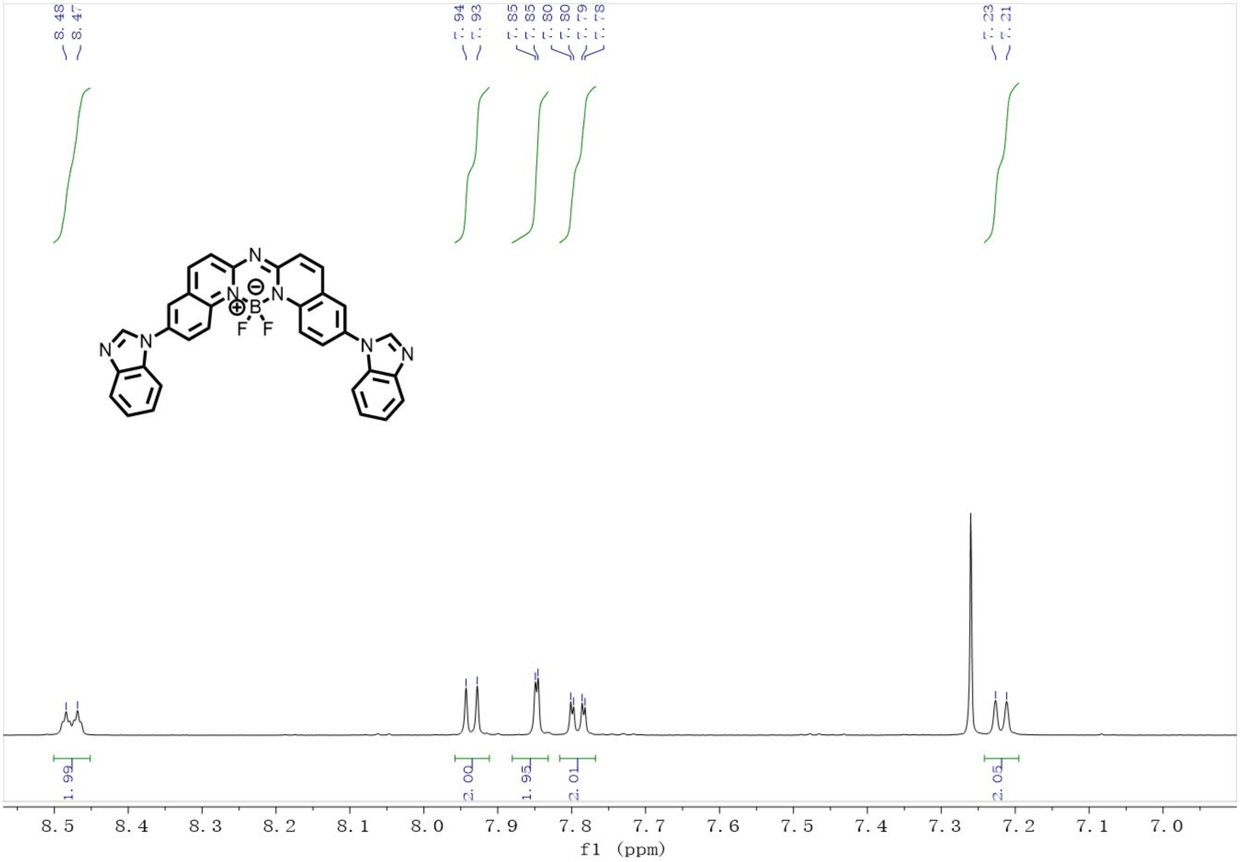

Figure S36. ^{1}H NMR spectrum of **BODIQU-BZI** in $CDCl_3$.

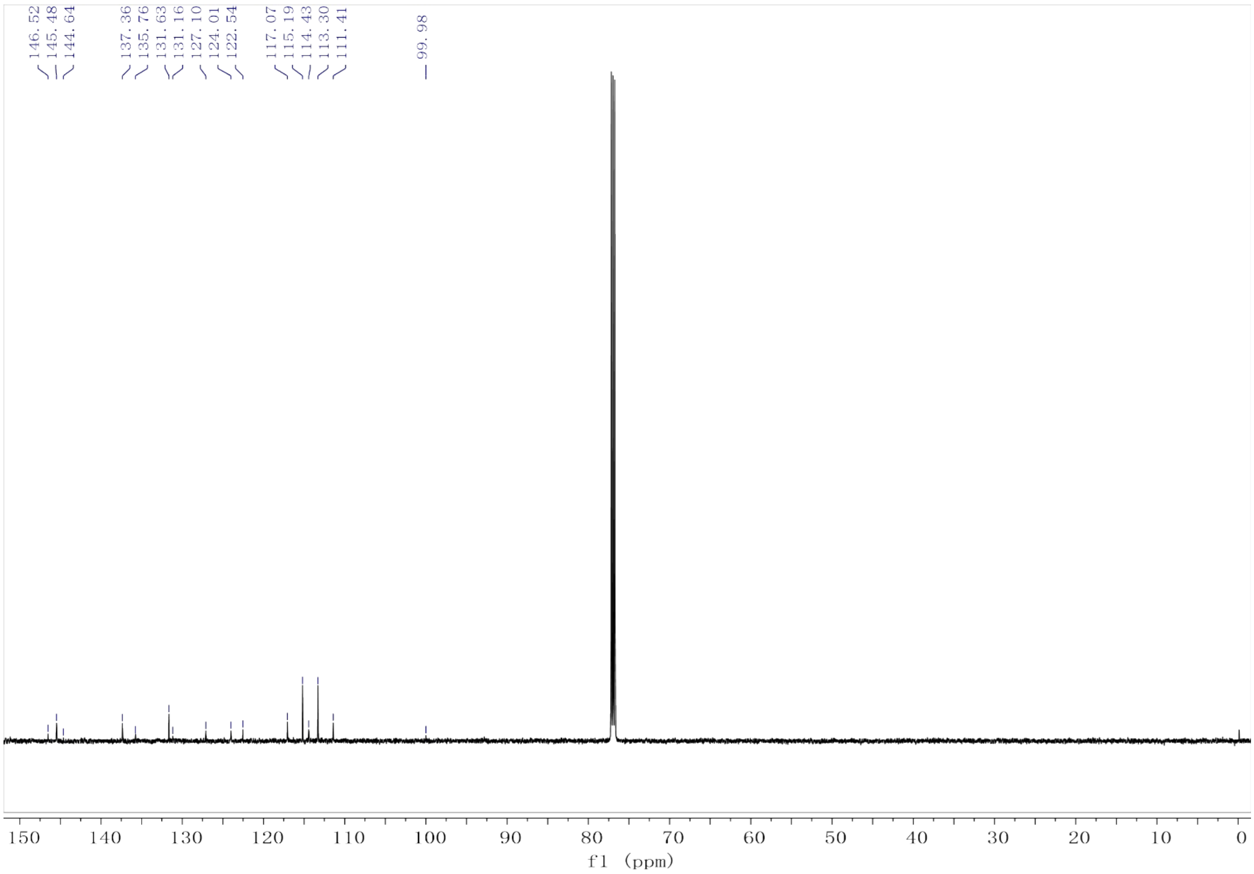

Finger S37. ^{13}C NMR spectrum of **BODIQU-BZI** in $CDCl_3$.

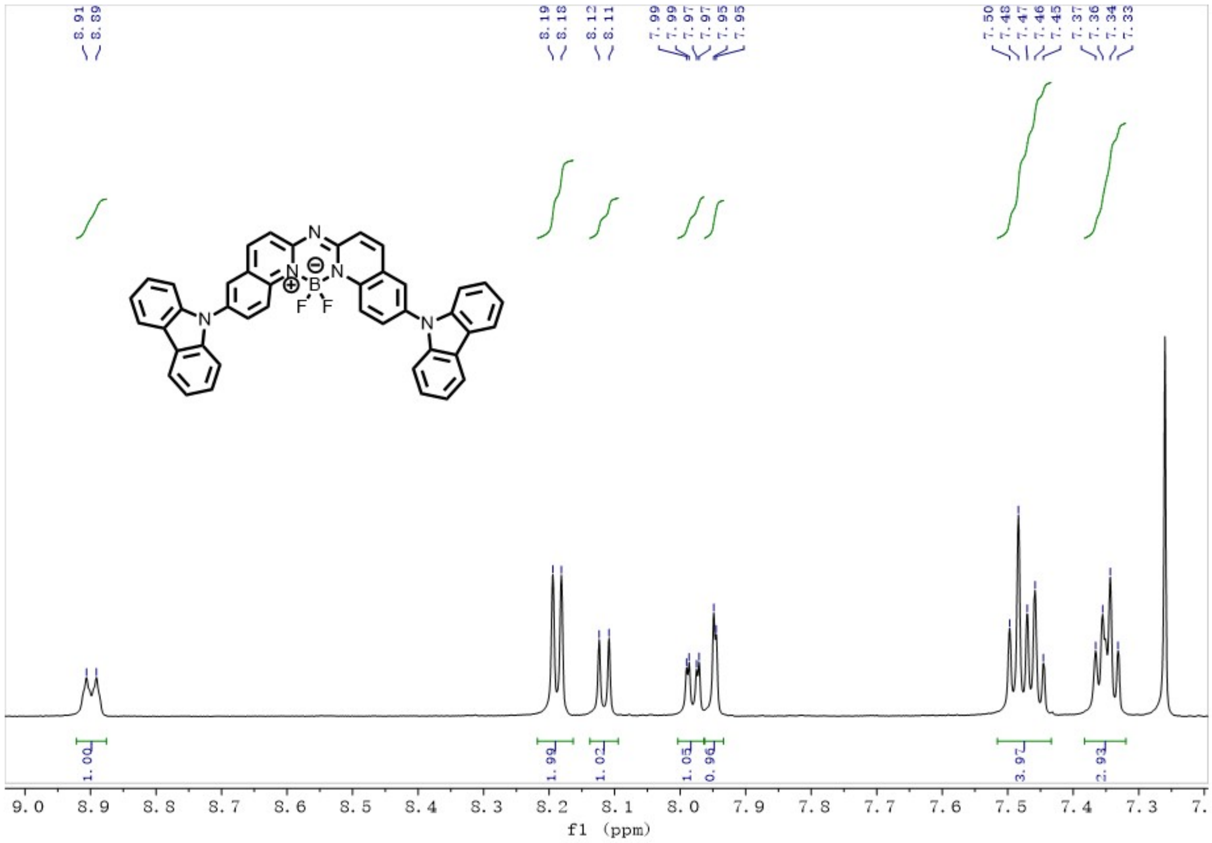

Figure S38. ^{1}H NMR spectrum of **BODIQU-CZ** in $CDCl_3$.

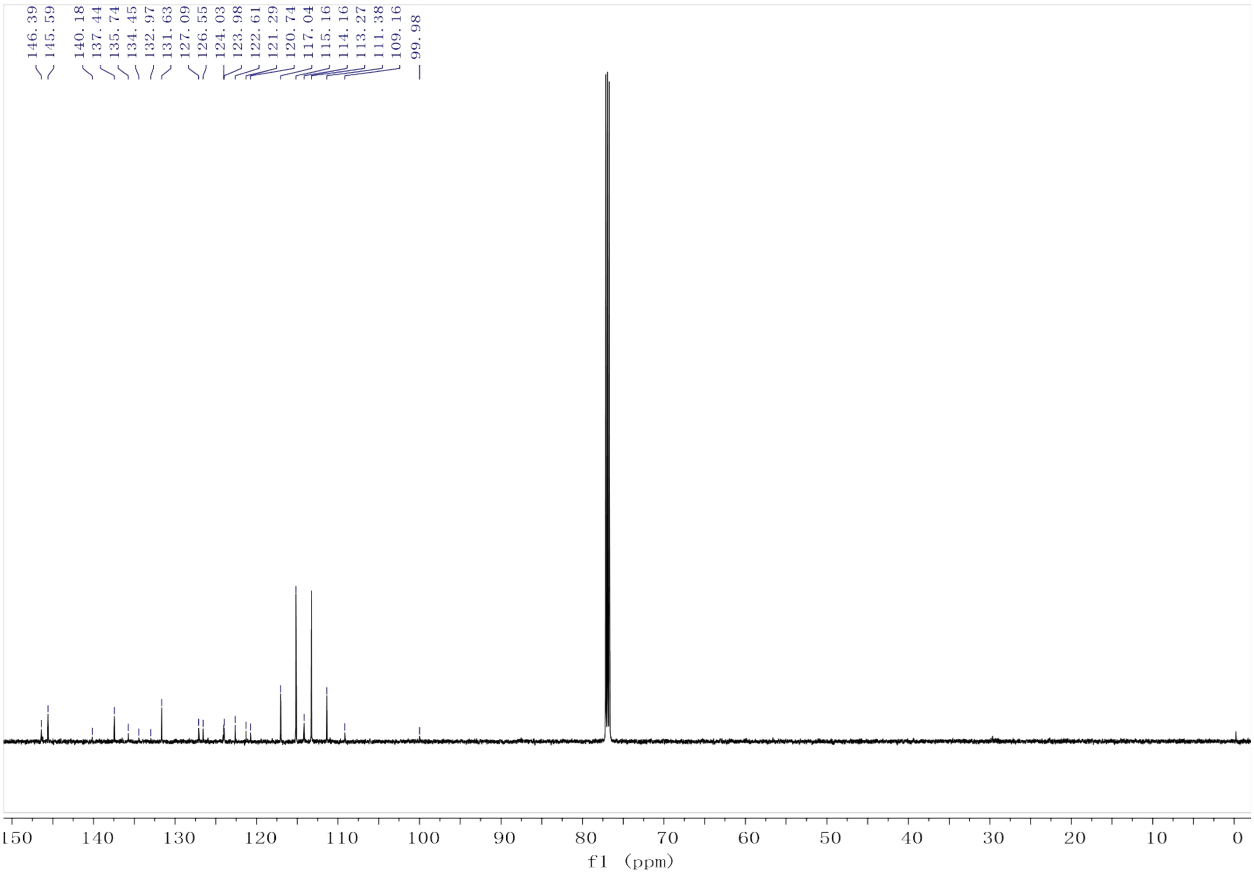

Finger S39. ^{13}C NMR spectrum of **BODIQU-CZ** in $CDCl_3$.

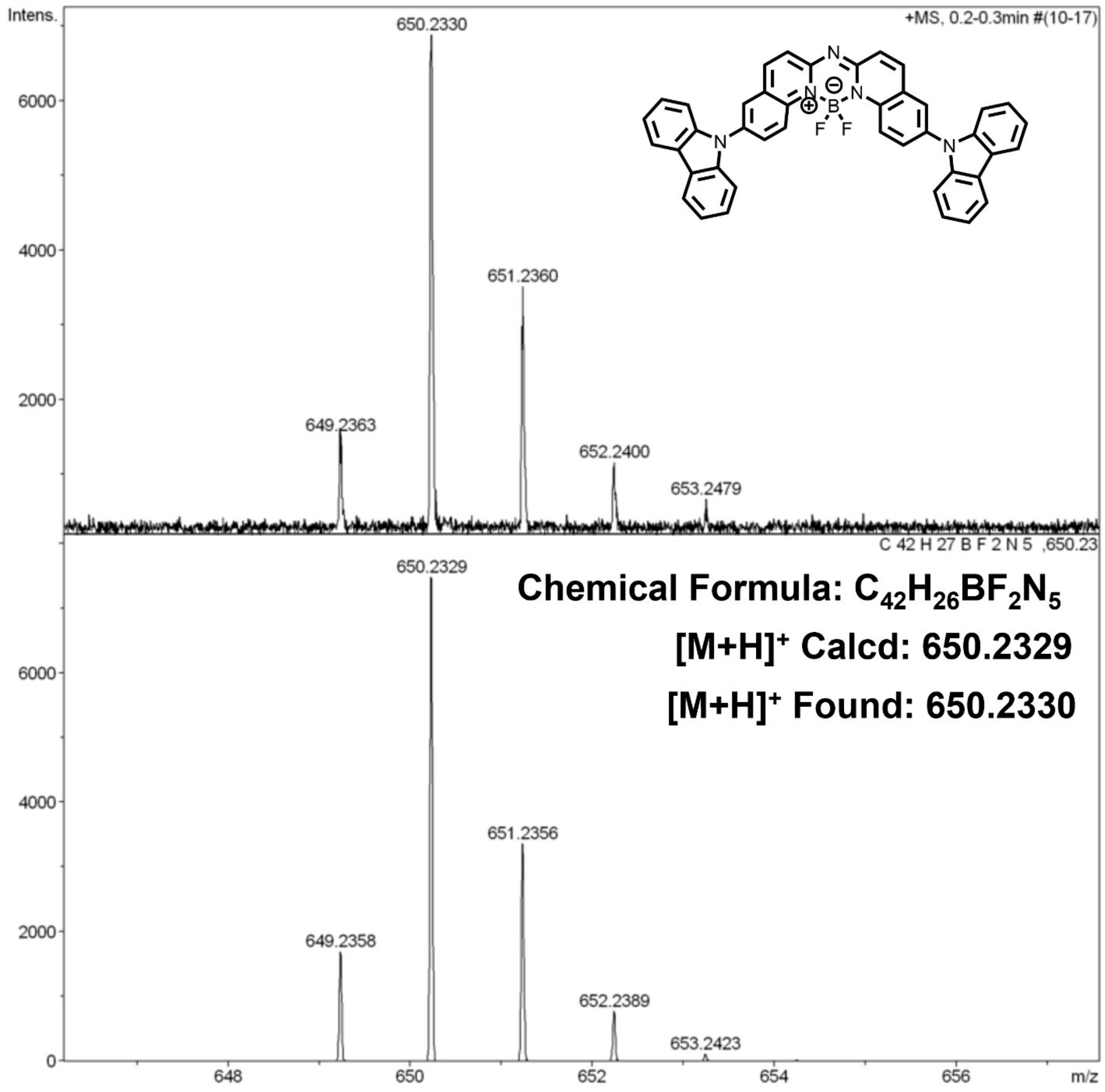

Finger S40. HRMS spectrum of **BODIQU-CZ**.

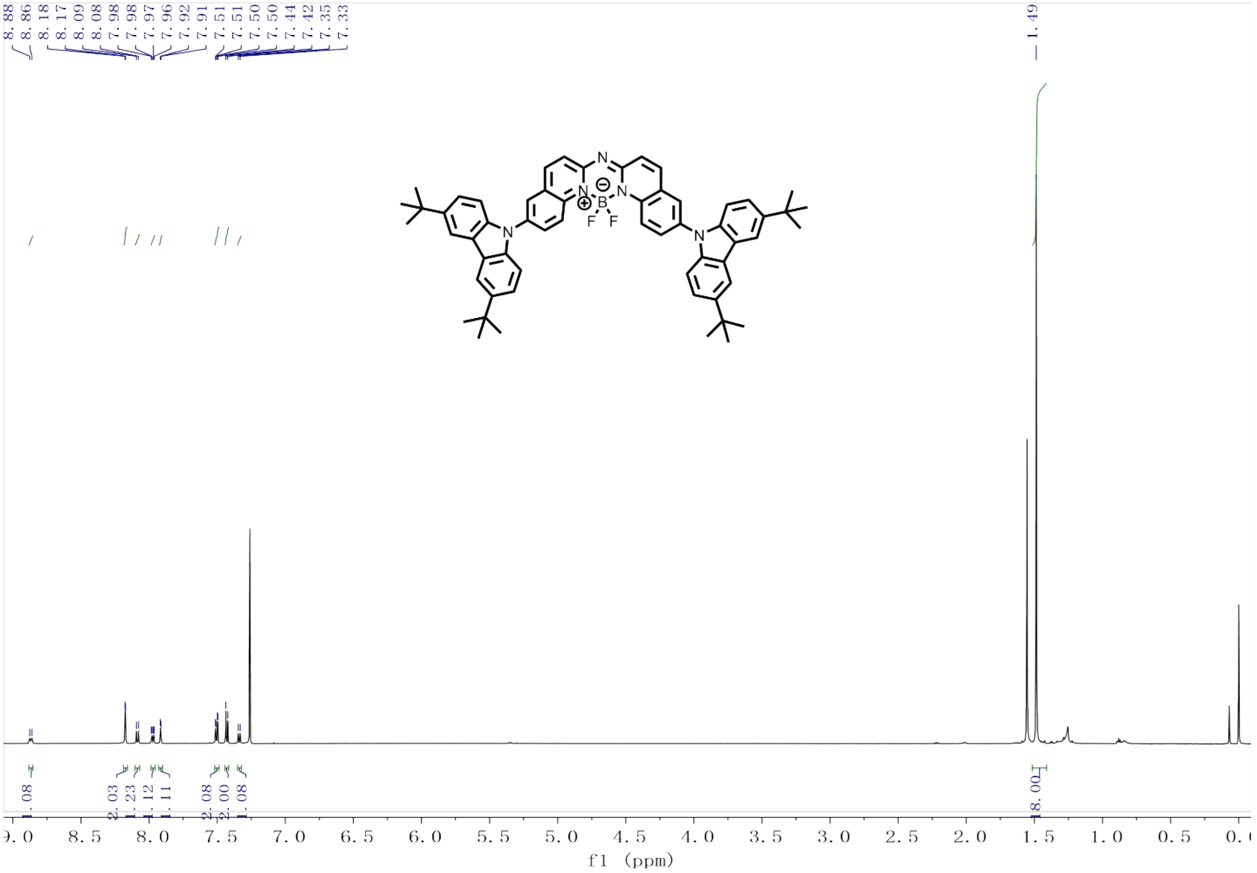

Figure S41. ^{1}H NMR spectrum of **BODIQU-tBuCZ** in $CDCl_3$.

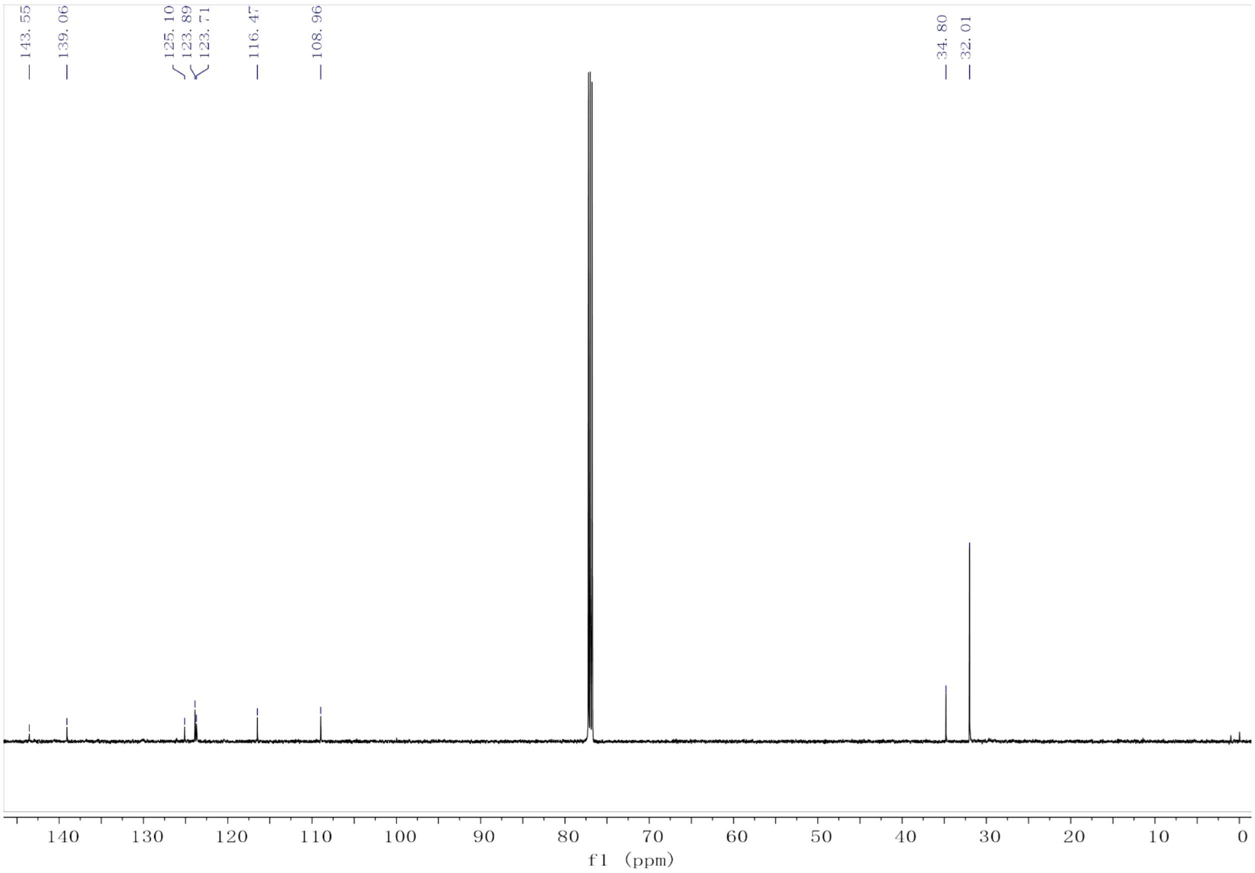

Finger S42. ^{13}C NMR spectrum of **BODIQU-tBuCZ** in $CDCl_3$.

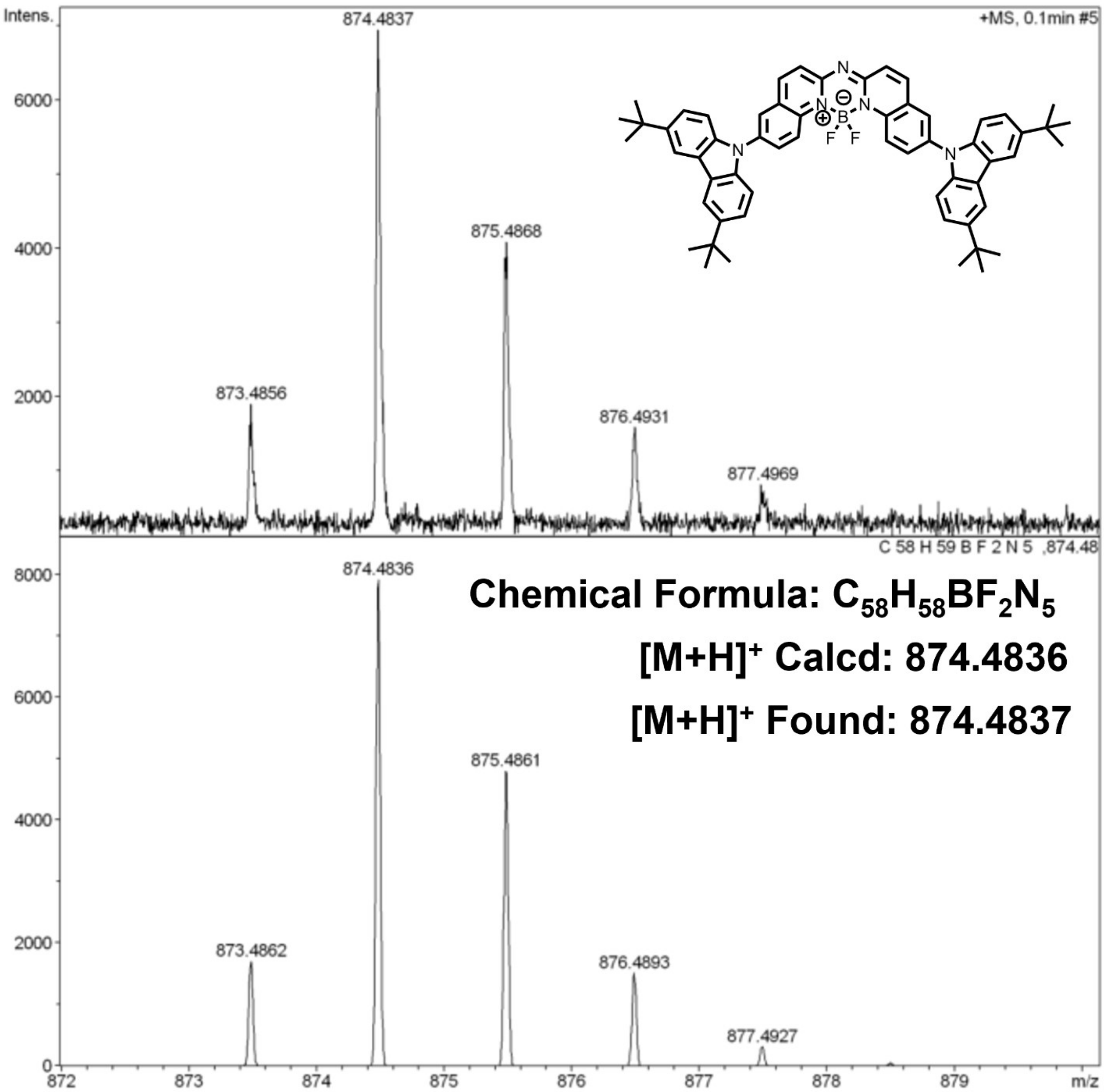

Finger S43. HRMS spectrum of **BODIQU-tBuCZ**.

13.References

1. D. Wang, R. Liu, C. Chen, S. Wang, J. Chang, C. Wu, H. Zhu and E. R. Waclawik, Synthesis, photophysical and electrochemical properties of aza-boron-diquinomethene complexes. *Dyes Pigm.*, **2013**, *99*, 240-249.
2. Z. Zhou, L. Zhang, L. Peng, Y. Li, X. Zhu, Y. Wu, Z. Qiu, G. He, M. Qin, H. Peng and Y. Fang, Dynamic response and discrimination of gaseous sarin using a boron-difluoride complex film-based fluorescence sensor. *Aggregate*, **2024**, e629.
3. Z. Qiu, Y. Xiao, L. Zhang, Y. Miao, B. Zhang, X. Zhu, L. Ding, H. Peng and Y. Fang, Highly sensitive and selective detection of DCP vapors using pyridine-based fluorescent nanofilms. *Chem. Commun.*, **2024**, *60*, 9773-9776.
4. K. Liu, M. Qin, Q. Shi, G. Wang, J. Zhang, N. Ding, H. Xi, T. Liu, J. Kong and Y. Fang, Fast and selective detection of trace chemical warfare agents enabled by an ESIPT-based fluorescent film sensor. *Anal. Chem.*, **2022**, *94*, 11151-11158.
5. W. Mo1, Z. Zhu, F. Kong, X. Li, Y. Chen, H. Liu, Z. Cheng, H. Ma and B. Li, Controllable synthesis of conjugated microporous polymer films for ultrasensitive detection of chemical warfare agents. *Nat. Commun.*, **2022**, *13*, 5189.
6. R. Zhu, J. M. Azzarelli and T. M. Swager, Wireless hazard badges to detect nerve-agent simulants. *Angew. Chem. Int. Ed.*, **2016**, *55*, 9662-9666.
7. P. Zheng, A. Abdurahman, Z. Zhang, Y. Feng, Y. Zhang, X. Ai, F. Li and M. Zhang, A simple organic multi-analyte fluorescent prober: One molecule realizes the detection to DNT, TATP and Sarin substitute gas. *J. Hazard. Mater.* **2021**, *409*, 124500.
8. P. Zheng, Z. Cui, H. Liu, W. Cao, F. Li and M. Zhang, Ultrafast-response, highly-sensitive and recyclable colorimetric/fluorometric dual-channel chemical warfare agent probes. *J. Hazard. Mater.* **2021**, *415*, 125619.
